# Complete chloroplast genome of *Triticum aestivum* cultivar ‘Keumkang’ from Korea (Poaceae) and comparative chloroplast genomes of the members of the *Triticum* genus

**DOI:** 10.1002/jsfa.70489

**Published:** 2026-02-11

**Authors:** Kang‐Rae Kim, Changhyun Choi, Jung Sun Kim, Myung‐Hee Kim, Hwa Jin Jung, DaHye Jeon, Jin‐Hyun Kim

**Affiliations:** ^1^ Southeast Sea Fisheries Research Institute National Institute of Fisheries Science Namhae Republic of Korea; ^2^ Winter Crop Research Division National Institute of Crop Science, RDA Wanju Republic of Korea; ^3^ Digital Breeding Convergence Division National Institute of Agricultural Sciences, RDA Jeonju Republic of Korea

**Keywords:** next‐generation sequencing, DNA barcode, specific barcode, chloroplast genome, *Triticum aestivum* cultivar Keumkang

## Abstract

**BACKGROUND:**

Bread wheat (*Triticum aestivum* L.) is a major global food crop, and understanding its maternal lineage and genetic diversity is essential for breeding, authentication, and evolutionary studies. Chloroplast genomes provide valuable markers for phylogenetic inference and cultivar discrimination; however, conventional plant DNA barcodes often lack sufficient resolution within the genus *Triticum*. This study aimed to characterize the complete chloroplast genome of the Korean wheat cultivar ‘Keumkang’ and to develop effective chloroplast‐based barcode markers for improved identification of *Triticum* species and cultivars.

**RESULTS:**

The complete chloroplast genome of *T. aestivum* cv. Keumkang was assembled using PacBio HiFi reads and determined to be 135 909 bp in length, exhibiting a typical quadripartite structure. Comparative analyses of 44 *Triticum* and related *Aegilops* chloroplast genomes revealed that Keumkang shared an identical chloroplast genome structure with several Korean cultivars, indicating a common maternal origin. Phylogenomic analysis placed *T. aestivum* in close association with *T. turgidum* subsp. *durum*, supporting its maternal derivation from the AABB genome lineage. Nucleotide diversity analysis identified six coding sequences and 11 intergenic regions with relatively high polymorphism. Based on these regions, 17 chloroplast‐specific barcode markers were developed and experimentally validated. While conventional barcodes (matK, rbcL, trnL–F) achieved only approximately 18% cultivar discrimination, the combined use of the 17 newly developed markers improved identification accuracy to 50% among the examined accessions.

**CONCLUSION:**

The complete chloroplast genome of *T. aestivum* cv. Keumkang provides new insights into the maternal lineage and chloroplast diversity of wheat. The newly developed set of 17 chloroplast barcode markers substantially enhances cultivar‐level discrimination within the genus *Triticum* and represents a useful molecular tool for wheat breeding, germplasm authentication, and evolutionary studies. © 2026 The Author(s). *Journal of the Science of Food and Agriculture* published by John Wiley & Sons Ltd on behalf of Society of Chemical Industry.

## INTRODUCTION

Wheat (*Triticum* spp. L.) is a staple crop that plays a critical role in global nutrition.^1^ Approximately 770 million metric tons (Mt) of wheat are produced annually worldwide, reflecting its importance as a versatile ingredient in a wide range of products.^1^ The genus *Triticum* represents an allopolyploid complex of significant agricultural importance, consisting of two diploid, two tetraploid, and two hexaploid species.^1^ Among these, one diploid and both tetraploid species were domesticated, while the two hexaploid species emerged under cultivation in Eurasia around 10 000 years ago.^2^

Hexaploid common wheat, or bread wheat (*T. aestivum*, AABBDD genomes), originated in the Caspian Sea region through hybridization between cultivated tetraploid *T. turgidum* (AABB genomes) and diploid *Aegilops tauschii* (DD genome).^3^, ^4^ In recent decades, researchers have increasingly focused on wheat's genetic diversity, particularly its chloroplast genome, which provides essential insights into species identification and phylogenetic studies.^3^, ^5^ Chloroplast genomes are valuable due to their small size, high copy number, uniparental inheritance, and low recombination and mutation rates.^6^ These properties make them particularly suitable for resolving complex phylogenetic relationships in angiosperms and understanding evolutionary history across various plant families.^7^, ^8^

Keumkang, a Korean winter wheat cultivar, was bred for desirable phenotypic traits, including rapid heading and maturation.^9^ This variety was developed to fit the farming practices of early wheat planting before rice cultivation on the Korean Peninsula.^9^ Additionally, Keumkang offers superior moisture and cold resistance compared to conventional wheat varieties, with faster planting and maturation times.^9^

Artificial hybridization is the primary method used in wheat breeding to enhance resistance to biotic and abiotic stresses, improving yield and adaptation to agricultural practices.^1^ *Triticum aestivum* cv. Keumkang was specifically selected for traits that accelerate heading and maturation.^9^ Despite the importance of understanding both paternal and maternal lineages in breeding, information on these lines remains limited.^9^

In plants, the maternal lineage is primarily represented in the chloroplast genome.^10^ This genome plays a critical role in the evolutionary tree due to its low intraspecies variation but significant interspecies variation.^11^ Consequently, tracing maternal chloroplasts is crucial for wheat breeding, as photosynthetic traits are entirely maternally inherited.^4^ The wheat genome has a complex evolutionary history, encompassing diploid, tetraploid, and hexaploid species.^4^ Cultivated wheat was domesticated from wild relatives, and various species have dominated agricultural history.^4^ Previous studies have clarified the relationship between chloroplast SSRs and wheat's complex chloroplast genome. Hexaploid wheat's AABBDD genome consists of the A genome from *T. urartu*, the B genome (derived from the S genome of *Aegilops speltoides*), and the D genome from *A. tauschii*.^4^ Hybridization between the AABB genome of *T. turgidum* subsp. *durum* and the D genome contributed to the origin of modern *T. aestivum*.^4^

DNA sequence analysis has emerged as a modern approach for studying evolutionary relationships and biodiversity.^12^, ^13^ DNA barcoding, which utilizes short, high‐resolution sequences, is critical for identifying plant species with complex taxonomic identities and investigating biodiversity.^14^, ^15^ Key loci proposed for plant DNA barcoding include maturase K (*matK*), internal transcribed spacer (ITS), and ribulose‐bisphosphate carboxylase (*rbcL*).^16^, ^17^ Additionally, combinations such as (*rpoC1 + rpoB + matK*), (*rpoC1 + matK + trnH‐psbA*), (*rbcL + trnH‐psbA*), and (*atpF‐H + psbK‐I + matK*) have been proposed.^18^, ^19^, ^20^ Among these, *matK* has been suggested as a standard DNA barcode.^21^ Specific barcode markers, which allow precise identification of varieties, are essential tools for studying the *Triticum* genus and its evolutionary relationships.^22^ However, existing studies have largely focused on SSR markers and the ndhF region of the chloroplast genome.^3^, ^4^, ^23^, ^24^, ^25^, ^26^, ^27^, ^28^, ^29^, ^30^, ^31^

In this study, the complete chloroplast genome of *Triticum aestivum* cv. Keumkang was sequenced and analyzed, revealing significant genetic variation among 44 *Triticum* species and cultivars. This analysis uncovered notable differences from *T. aestivum* cv. Chinese Spring and identified six coding sequences (CDS) and 11 intergenic regions with high polymorphism. Traditional barcoding markers, such as *matK*, *rbcL*, and *trnL‐F*, achieved low identification accuracy (~18%) for *Triticum* varieties using maximum likelihood (ML) tree analysis. However, a newly developed set of 17 combination markers nearly doubled the accuracy, providing an effective tool for species identification and genetic studies within the *Triticum* genus. These markers were successfully applied to *T. aestivum* cv. Keumkang, offering valuable resources for advancing genetic research and breeding programs in wheat and related species.

## MATERIALS AND METHODS

### Plant sampling and sequencing

**Figure 1:**
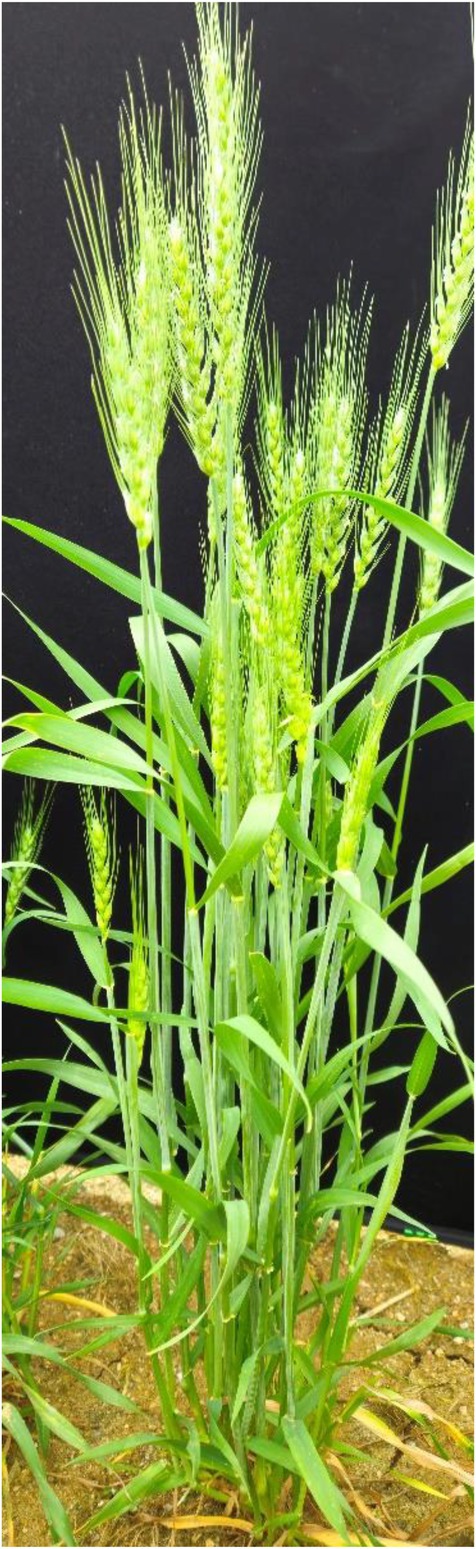
Photo of a specimen of *Triticum aestivum* cv. Keumkang.

*Triticum aestivum* cv. Keumkang leaves were collected from Jeonju, Korea (35° 49′ 51″ N, 127°03′48″ E, Fig. 1). The sample, assigned voucher number IT 213100, was deposited in the Genebank (https://genebank.rda.go.kr) of the National Institute of Agricultural Sciences. Keumkang is a registered bread wheat cultivar, and because bread wheat is predominantly self‐pollinating the Genebank (https://genebank.rda.go.kr) accession is maintained as a genetically stable line rather than a hybrid population; sequencing was performed using a single Genebank (https://genebank.rda.go.kr) accession to minimize within‐sample heterogeneity. Genomic DNA was extracted from the leaves using the CTAB method according to the manufacturer's instructions.^32^, ^33^ A PacBio HiFi sequencing library was prepared following the standard protocol, and sequencing was performed using a 20 kb library.

### Chloroplast genome assembly and annotation

The *T. aestivum* cv. Keumkang chloroplast genome was assembled from raw long reads using OATK version 1.0 (https://github.com/c-zhou/oatk). The complete genome was annotated with CPGAVAS2,^34^ based on *T. aestivum* cv. Keumkang (MW889058). Annotation errors were manually corrected using Geneious version 11.0.1 (https://www.geneious.com/). The finalized chloroplast genome sequence was submitted to GenBank under accession number PP829256 using NCBI BankIt. An illustration of the *T. aestivum* cv. Keumkang chloroplast genome was created with OrganellarGenomeDRAW.^35^

### Simple sequence repeat analysis

Simple sequence repeat (SSR) regions in the chloroplast genome were identified using the MIcroSAtellite (MISA) tool.^36^ The SSR motif thresholds were set to 25, 15, 2, 2, 2, and 2 for mono‐, di‐, tri‐, tetra‐, penta‐, and hexa‐nucleotides, respectively. Duplicates and errors in the identified SSR regions were manually reviewed and corrected.

Long repeat sequences were analyzed using the Repeat Finder function in Geneious version 11.0.1 (https://www.geneious.com/). The identification parameters were set to a minimum repeat size of 30 bp with 100% sequence identity.

### Phylogenetic analysis

Phylogenetic trees were inferred using ML and Bayesian inference (BI). For ML, the best‐fit substitution model was selected using jModelTest 2.1.7 (TPM1uf + G + I),^37^ and the ML tree was reconstructed in PhyML 3.0^38^ with 1000 bootstrap replicates. All trees were rooted using a non‐Triticeae Poaceae outgroup included in the dataset (Supporting Information, Table S1) to ensure consistent rooting across analyses, and branch lengths were retained to represent substitutions per site. In addition, to generate a combined barcode dataset, sequences for each of the 17 loci were aligned separately across all accessions, trimmed to retain only homologous positions, and then concatenated in a fixed locus order to produce a single combined alignment for each accession. This concatenation increases the number of informative sites relative to any single locus and thereby improves cultivar‐level discrimination in phylogenetic identification.

For the BI analysis, MrBayes version 3.2.7^39^ was employed under the GTR substitution model (lset nst = 6). The analysis utilized a Markov chain Monte Carlo (MCMC) algorithm, running for 10 million generations with sampling every 100 generations. The first 25% of samples were discarded as burn‐in. Posterior probabilities for each node were calculated based on the consensus of multiple trees.

The phylogenetic trees produced by both methods were combined into consensus trees, with support values represented as bootstrap values for the ML tree and posterior probabilities for the BI tree. This comprehensive approach ensured robust phylogenetic reconstruction and reliable node support.

### Comparative chloroplast genome analysis, hotspot identification, and marker development

Chloroplast genome sequences of *Triticum* species and cultivars were obtained from NCBI (Supporting Information, Table S1) and aligned using MAFFT version 7.490.^40^ From this MAFFT alignment, CDS and intergenic regions were extracted using Geneious version 11.0.1 (https://www.geneious.com/), and nucleotide diversity (Pi) was calculated using DnaSP version 6.12^41^ to identify candidate hotspot loci for subsequent primer design and polymerase chain reaction (PCR) validation (see the following subsection).

Primers for effective molecular markers were designed based on identifiable intergenic regions of *Triticum* species and cultivars. Using the MAFFT multiple alignment described above (Supporting Information, Table S1), hotspot loci were selected and primers were designed to amplify these regions for downstream PCR validation.

From the DNA barcode regions (coding sequences and intergenic regions), six CDS (Pi ≥0.00015) and 11 intergenic regions (Pi ≥ 0.0001) showing relatively high interspecific nucleotide diversity were identified as candidate authentication hotspots. Primers were anchored in conserved flanking segments to support robust amplification across taxa, while the internal segments were selected to capture species‐informative variation, producing PCR products ranging from 250 to 1500 bp.

To assess the effectiveness of species identification using the specific barcode markers, two methods were employed: a phylogenetic tree‐based method and a sequence similarity‐based method. For the phylogenetic tree‐based approach, ML trees were constructed using the 17 specific barcode region sequences and compared with a tree derived from the full chloroplast genome to evaluate consistency.

Amplification of 17 specific barcoding markers was performed using a T100 Thermal Cycler (Bio‐Rad, Hercules, CA, USA). PCR was performed in a total volume of 20 μL using Solg Taq DNA polymerase (SolGent, Daejeon, Korea). Specific barcoding marker was forward primer 1 μL (1.0 μmol L^−1^), reverse primer 1 μL (1.0 μmol L^−1^), Taq DNA polymerase 0.12 μL (5 U μL^−1^), 10× Taq reaction buffer 2.5 μL (25 mmol L^−1^ MgCl_2_ mixed), 10 mmol L^−1^ dNTP mix 1 μL, template DNA 10 ng μL^−1^, and 3DW 13 μL. PCR conditions were 94 °C for 5 min, 94 °C for 30 s, 54 and 58 °C for 30 s, and 72 °C for 30 s, 34 cycles. Final elongation was at 72 °C for 7 min. The final PCR product was electrophoresed on a 1.5% agarose gel, and a single band was identified using UVP GelSolo (UVP GelSolo, Analtytik Yena, Jena, Germany).

In July 2024, 18 cultivars of *Triticum* and *Aegilops* were planted and sprouted, and genomic DNA extracted from leaves as described above was used to verify the 17 specific barcoding markers (Supporting Information, Table S2).

## RESULTS

### Chloroplast genome characterization for *T. aestivum* cv. Keumkang

**Figure 2:**
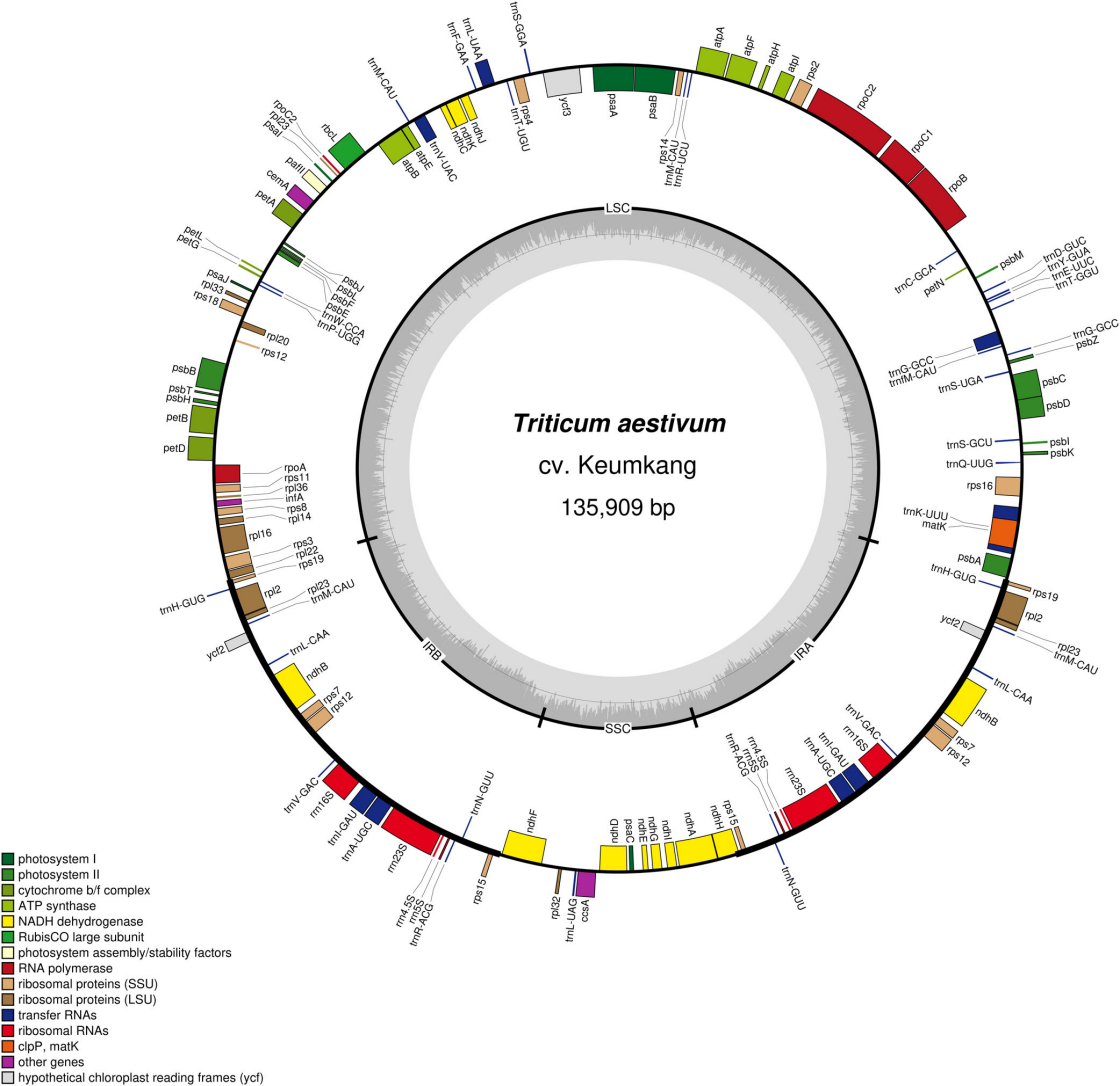
Structure of the complete chloroplast genome of *T. aestivum* cv. Keumkang from Korea.

Whole‐genome sequencing of *T. aestivum* cv. Keumkang using the PacBio HiFi platform produced 958 million reads, yielding a total of 144 GB of data. The complete chloroplast (cp) genome of *T. aestivum* cv. Keumkang was assembled using the reference genome of *T. aestivum* cv. Keumkang (NCBI Accession No. MW889058). The cp genome was 135 909 bp in length and exhibited a typical quadripartite structure (Fig. 2), consisting of a large single‐copy (LSC) region (80 014 bp), a small single‐copy (SSC) region (12 791 bp), and two inverted repeat (IR) regions (each 21 552 bp; IRa and IRb) (Table 1).

**Table 1:** Chloroplast genome information of *Triticum aestivum* cv. Keumkang

Species	*T. aestivum* cv. Keumkang
Chloroplast genome length (bp)	135 909
Large single‐copy length (bp)	80 014
Small single‐copy length (bp)	12 791
Inverted repeat length (bp)	21 552
Total number of genes	132

**Figure 3:**
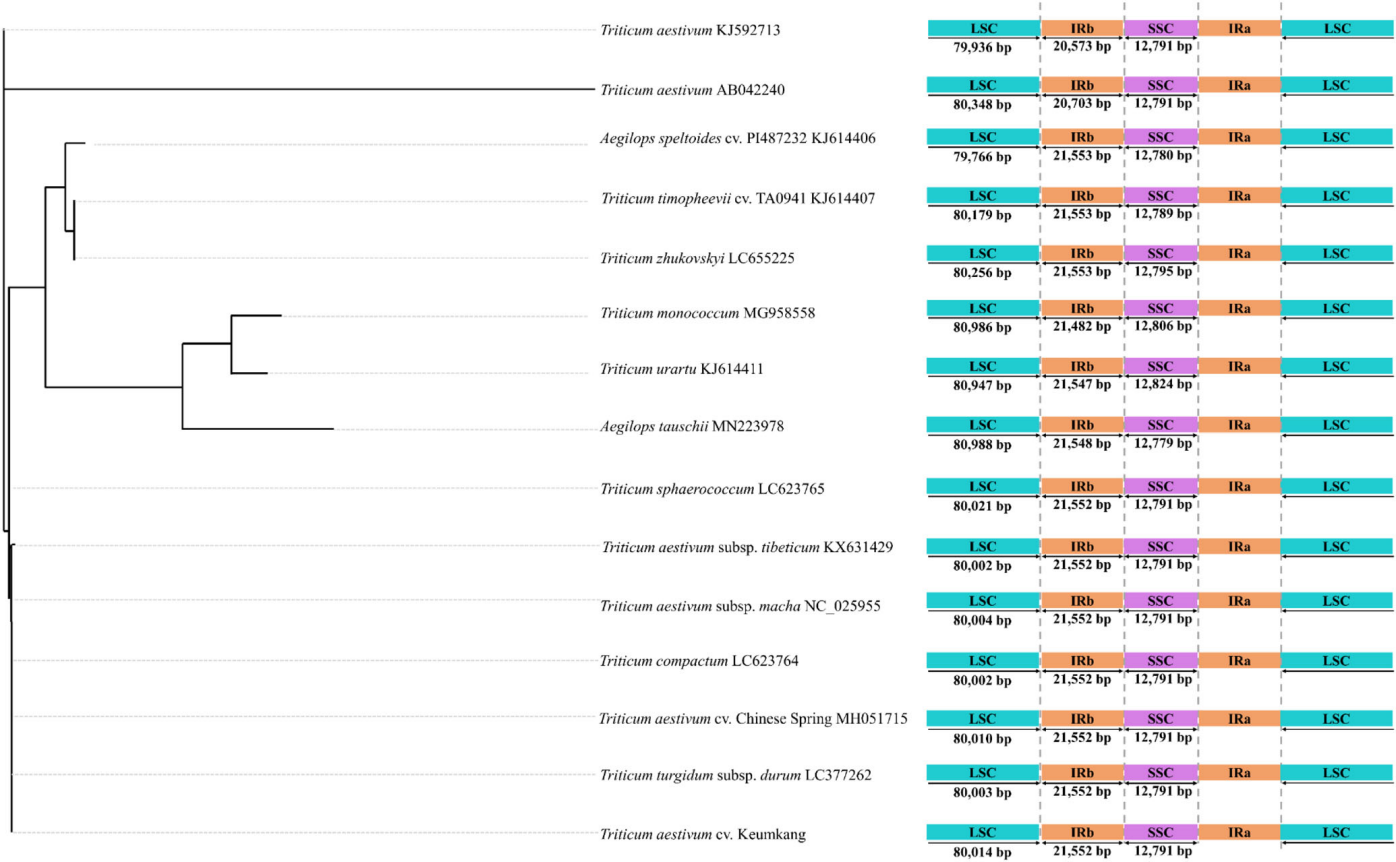
Descriptive ordering of selected *Triticum* and *Aegilops* accessions for visualization of chloroplast genome region lengths (LSC, large single‐copy; IR, inverted repeat; SSC, small single‐copy) and junction‐associated variation. The left‐hand panel is not a rooted phylogeny and is not used to infer evolutionary relationships; it serves only to align accessions with the structural summaries shown on the right.

The structural features of the chloroplast genomes of 44 accessions within the *Triticum* and *Aegilops* genera, including genome length, LSC, SSC, and IR region sizes, as well as the total number of genes, are summarized in Supporting Information, Table S3. In *Triticum* species, variations in chloroplast genome length reflected small differences in the lengths of the LSC, SSC, and IR regions (Fig. 3), which are consistent with short indels and minor shifts at junction positions reported among closely related chloroplast genomes. For instance, although *T. aestivum* accessions KJ592713 and AB042240 belong to the same species, only small length differences were observed (412 bp in the LSC region and 130 bp in the IR region), which are better described as short indel variation rather than genome expansion. Within the genus *Triticum*, species such as *T. aestivum* subsp. *tibeticum*, *T. sphaerococcum*, *T. compactum*, *T. aestivum* cv. Chinese Spring, *T. aestivum* subsp. *macha*, *T. aestivum* cv. Keumkang, and *T. turgidum* subsp. *durum* exhibited identical lengths for the IR and SSC regions, with differences only in the LSC region. Among these, *A. tauschii* (D genome) had the shortest SSC region, while *A. speltoides* (S genome) had the shortest LSC region. Comparatively, *T. zhukovskyi* displayed minor length differences in the LSC, IR, and SSC regions when compared to *T. timopheevii* and *T. monococcum*.

*Triticum aestivum* cultivars Keumkang, Saekeumkang, Sooan, Baegjoong, Goso, and Jokyoung shared identical genome sizes for their LSC, IR, and SSC regions (Supporting Information, Table S3). However, two *T. aestivum* accessions annotated as cv. Chinese Spring showed a 6 bp difference in the LSC region, despite belonging to the same species (*T. aestivum*). Additionally, *T. aestivum* var. *vavilovii* exhibited a 48 bp difference in the LSC region.

Several publicly available chloroplast genome records, including *T. aestivum* (KC912694), *T. monococcum* (KY636171), *T. urartu* (NC_021762), *A. cylindrica* (NC_023096), *A. geniculata* (NC_023097), *A. speltoides* (JQ740834), and *A. tauschii* (NC_022133), did not explicitly represent the canonical quadripartite structure (LSC/IR/SSC/IR) in their deposited sequence features. Accordingly, gene counts inferred directly from these records' annotations appeared lower than those from chloroplast genomes with clearly defined quadripartite annotations, and this should not be interpreted as experimentally validated evidence for a physically IR‐lacking chloroplast genome topology.

Across the remaining *Triticum* and *Aegilops* chloroplast genomes with explicit quadripartite annotations, 132 genes were consistently identified. Overall, these results indicate that differences in database representation and annotation of chloroplast genome structure, particularly IR representation, can influence apparent gene content estimates within these genera.

### SSR analysis for chloroplast genomes

#### Long repeats analysis

**Figure 4:**
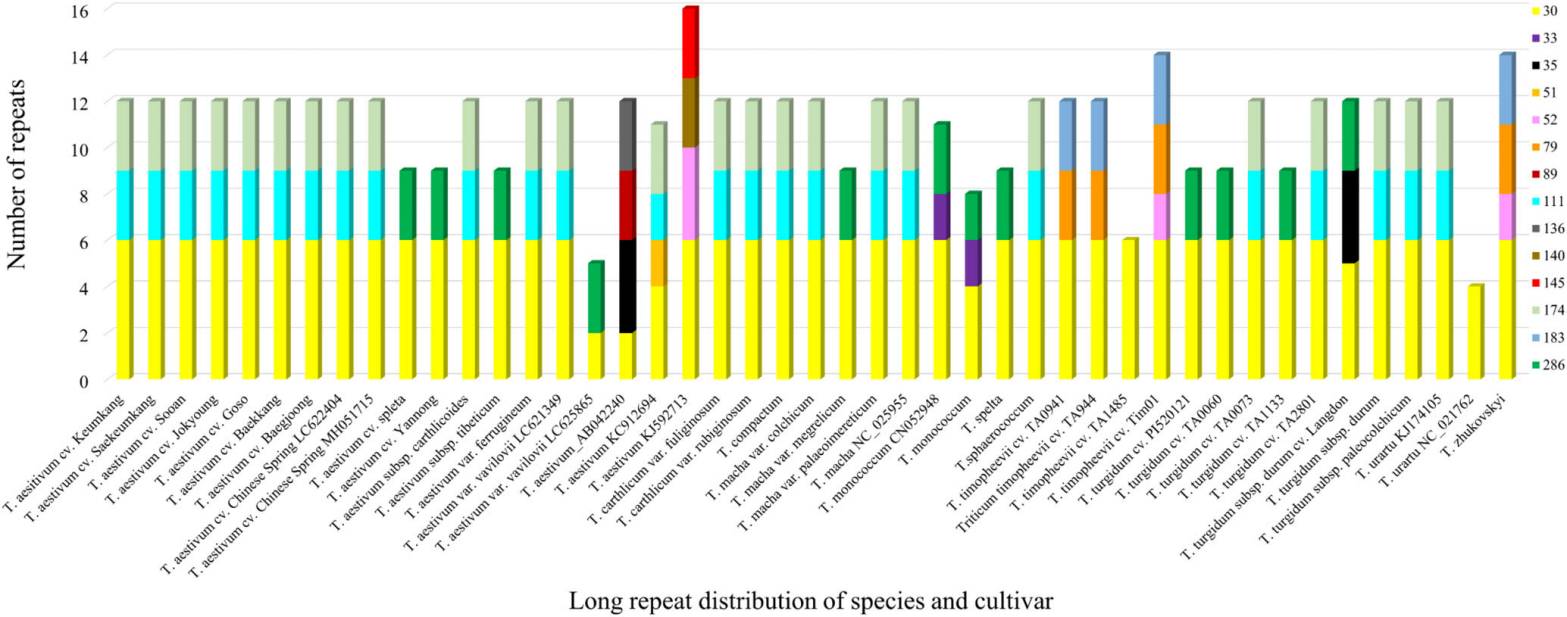
Repeat information based on distribution of the number of long repeats of species.

Detailed distribution data of long repeats by cultivar and species are provided in Supporting Information, Table S4. While *T. aestivum* is the same species, differences in the distribution of long repeats were observed among accessions AB042240, KC912694, and KJ592713 (Fig. 4). Similarly, even within *T. aestivum* var. *vavilovii*, variations in long repeat distribution were detected, including a difference of four repeats for 30 bp long repeats and three repeats for 286 bp long repeats (Table S4).

Differences in the distribution of long repeats were also evident between cultivars of *T. aestivum*, such as Chinese Spring and spleta. In contrast, Yannong and *T. aestivum* subsp. *tibeticum* displayed the same repeat distribution as Yannong and spleta cultivars. These findings highlight the variation in repeat patterns even among closely related cultivars and subspecies.

#### SSR analysis

The motif distribution across species consisted of 71.98% trinucleotides, 14.97% tetranucleotides, 8.00% pentanucleotides, and 5.05% hexanucleotides (Supporting Information, Table S5). *Triticum aestivum* cultivars Keumkang, Saekeumkang, Sooan, Goso, and Baegjoong each contained 33 SSRs in the LSC region, while Jokyoung and Baekkang cultivars exhibited 32 SSRs, indicating a variation in the number of SSRs in the LSC region.

The number of tri‐, tetra‐, penta‐, and hexanucleotide repeats in the LSC, SSC, and IR regions varied across chloroplast genomes of *Triticum* species. For example, *T. aestivum* var. *vavilovii* (LC621349, LC625865) showed a difference of two SSR repeats between the same cultivars, with 32 and 34 SSRs in the LSC region, respectively. Similarly, *T. aestivum* accessions AB042240 and KJ592713 differed in trinucleotide motifs, with 33 and 32 repeats in the LSC region and 7 and 8 repeats in the IR region, respectively (Supporting Information, Table S5).

In *T. turgidum* cultivars, the SSR distribution was consistent across PI520121, TA0060, TA0073, and TA2801. However, TA1133 displayed one additional tetranucleotide repeat and one additional hexanucleotide repeat, highlighting differences in the LSC region. *Triticum turgidum* subsp. *durum* cv. Langdon had the lowest number of trinucleotide motifs among the *Triticum* species, with only five trinucleotide SSRs in the IR region (Supporting Information, Table S6).

These findings demonstrate the variability in SSR motifs among cultivars and subspecies, reflecting differences in chloroplast genome structures.

#### Phylogenetic analysis

**Figure 5:**
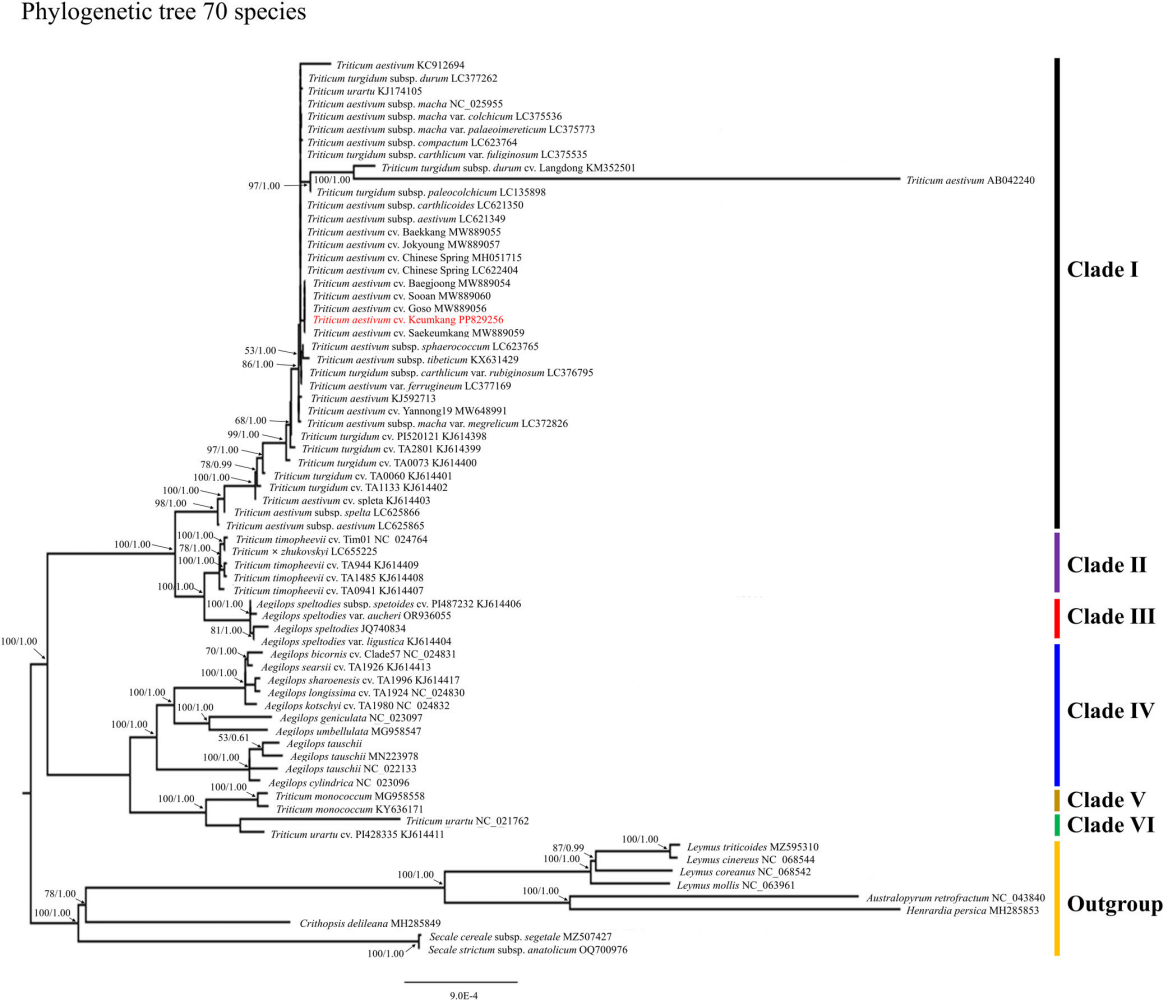
Phylogenetic trees comprising 70 species and cultivars in the family Poaceae using the maximum likelihood and Bayesian inference methods, based on the chloroplast genome sequence. The numbers above the nodes represent the bootstrap support value for each branch.

The dataset comprised chloroplast genomes from 70 Poaceae taxa (Fig. 5), and both ML and BI analyses recovered *Triticum* as a strongly supported monophyletic group. Trees were rooted with a non‐Triticeae Poaceae outgroup (Supporting Information, Table S1), and node support (ML bootstrap and BI posterior probability) together with branch lengths (substitutions per site) are shown in the phylograms. Notably, *T. aestivum* accession AB042240 displayed an extended terminal branch relative to other *T. aestivum* accessions, which is consistent with its accession‐level sequence differences reported in this study, and therefore is interpreted as within‐species divergence in variable regions rather than evidence of misalignment or taxonomic misidentification. Bootstrap values within Clade I showed strong support, exceeding 99% (Fig. 5).

Clade I included *T. aestivum* species and cultivars together with *T. turgidum* subsp. *durum* (AABB genome), reflecting their close chloroplast affinities. Clade II consisted of *T. timopheevii* and *Triticum* × *zhukovskyi*. Clade III formed a distinct lineage represented by *Aegilops speltoides*. Clade IV comprised *Aegilops* lineages including the D‐genome donor *A. tauschii*. Clade V contained the diploid A‐genome lineage *T. monococcum*, and Clade VI represented the A‐genome lineage *T. urartu*. In this chloroplast‐based tree, the diploid A‐genome *Triticum* lineages (*T. monococcum* and *T. urartu*) were placed closer to certain *Aegilops* lineages than to other *Triticum* groups.

These clades reflect the evolutionary relationships among *Triticum* and related *Aegilops* species, offering insights into the genomic structures and origins of these taxa.

### Chloroplast genome divergence regions and specific barcoding region screening

We multiple‐aligned CDS and intergenic sequences in the genus *Triticum* and screened protein‐coding regions and intergenic regions. Analyzing the nucleotide diversity of the CDS and intergenic regions, the nucleotide diversity (Pi) values of the CDS ranged from 0.00004 (*psaA*) to 0.00148 (*petG*). The most polymorphic genes were *rpl32*, *atpB*, *atpI*, *psbJ*, *ccsA*, *ndhA*, *rps14*, *matK*, *rpl22*, and *petG* (Pi ≥ 0.0005). The least polymorphic gene was *psaA*. The nucleotide diversity of the intergenic region ranged from 0.00000 (*psbC‐trnS‐UGA*) to 0.01613 (*rpl23‐psaI*).

**Figure 6:**
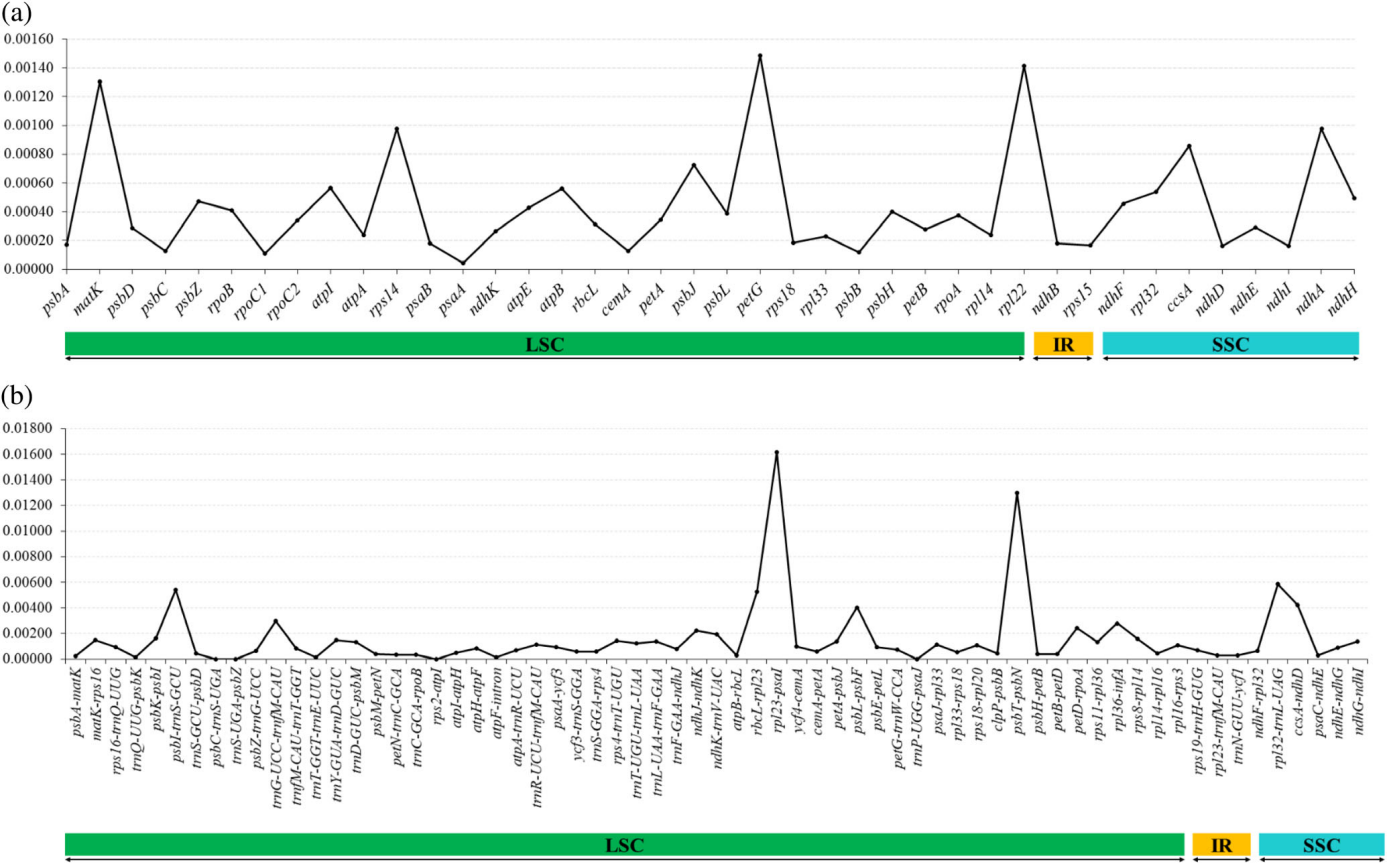
Nucleotide diversity in the entire chloroplast genome of 44 species and cultivars: (a) coding sequence; (b) intergenic region. LSC, large single‐copy; IR, inverted repeat; SSC, small single‐copy.

The regions with the highest nucleotide diversity in the intergenic region were *rps16‐trnQ‐UUG, psaA‐ycf3*, *psbE‐petL*, *ycf4‐cemA*, *rpl16‐rps3*, *rps18‐rpl20*, *trnR‐UCU‐trnfM‐CAU*, *psaJ‐rpl33, trnT‐UGU‐trnL‐UAA*, *rps11‐rpl36*, *trnD‐GUC‐psbM*, *trnL‐UAA‐trnF‐GAA*, *ndhG‐ndhI*, *petA‐psbJ*, *rps4‐trnT‐UGU*, *trnY‐GUA‐trnD‐GUC*, *matK‐rps16*, *rps8‐rpl14*, *psbK ‐psbI*, *ndhK‐trnV‐UAC*, *ndhJ‐ndhK*, *petD‐rpoA*, *rpl36‐infA*, *trnG‐UCC‐trnfM‐CAU*, *psbL‐psbF*, *ccsA‐ndhD*, *rbcL‐rpl23*, *psbI‐trnS‐GCU*, *rpl32‐trnL ‐UAG*, *psbT‐psbN*, and *rpl23‐psaI*. (Pi ≥ 0.0009; Fig. 6).

Comparison of nucleotide diversity showed that intergenic regions of 44 *Triticum* species were more variable than coding regions (Fig. 6). Specific hotspots within these variable regions were used to design primers to distinguish *Triticum* species and cultivars, with primer sites placed in conserved flanks. Candidate hotspot regions were defined based on nucleotide‐diversity thresholds (CDS Pi ≥0.00017 and intergenic Pi ≥0.00012), while also prioritizing loci that showed practical discriminatory variation among taxa. Because multiple accessions were not available for all species, we do not claim universal within‐species invariance across the entire genus; instead, we report the species‐informative variation profiles of the selected loci (Fig. 6) and validate their discriminatory performance using phylogenetic identification.

### Validation of specific barcoding regions for chloroplast genomes and marker amplification test

The primer sequences and PCR amplification conditions for the 17 developed chloroplast markers are presented in Table 2. These markers, including *ccsA*, *matK*, and *psbI‐trnS‐GCU*, were successfully amplified and utilized for tree‐based species identification within the genus *Triticum*. The BI and ML trees for each of the 17 markers are shown in Supporting Information, Fig. S1.

**Table 2:** Sequence and tree‐based species identification rate of existing specific barcode markers in the genus *Triticum*

No.	Region	Forward primer (5′–3′)	Reverse primer (5′–3′)	Product size range (bp)	Annealing temperature (°C)
1	*ccsA*	CGAGTGGCGGCATTCTTGAAA	CCTATCCGTTGACAGGGTAGA	1200–1300	54
2	*atpI*	GTTGCTGAGTTGAGAAAGAGATG	GTCAATTCAATGATGACCCTCCAT	800–850	58
3	*matK*	CCCTTTCCTGTTTCTTAATTTCG	CTGACCATATTGCACTATGTATCATC	1600–1650	54
4	*ndhH*	GTTTCTACCTCTACCCTATCTA	CAAATACCACAAAGAGCCAGCTAT	900–950	54
5	*psbA*	GGAACTTCAACAGCAGCTAAGTC	GCTTGGGAGTCCTTGCAATTTGA	1000–1100	58
6	*rpoA*	GATGCTTCTCTAGAGTGTCCCA	TAGTCTATTATGGTTCGAGAGG	900–1000	54
7	*matK‐rps16*	CTCGGTTTATCGAATGATGATAC	GCGTTGTTTATCTACATCTATCCC	1400–1500	58
8	*psbI‐trnS‐GCU*	CTAATGACCCAGGACGGAATC	CTTTCGCTTTGGAACGTGGA	250–300	58
9	*atpF‐intron*	TTTGGAAAGGGAGTGTGTGCGA	CCGCTTCTAGTTCGACTTTCTG	900–1000	58
10	*psaA‐ycf3*	GGTTCCGGCGAACGAATAATC	CACAGAACTGGTTGAAGATTACGA	700–800	58
11	*trnT‐UGU‐trnL‐UAA*	GGCTTACATAACGGAAATAGTG	CGTCTACCGATTTCGCCATATC	600–700	58
12	*trnL‐UAA‐trnF‐GAA*	GGGAAATGGGGATATGGCGAA	CTCTGCCAGGAACCAGATTTGAA	1100–1200	58
13	*petA‐psbJ*	CGCATCCGTTATTTTGGCACA	CATATTCTGGATTGGGTTCATC	900–950	58
14	*psbE‐petL*	CGTGCTTCCAGACATGCTG	GGCGTATCTTGTTCAAGCCAATA	1200–1300	58
15	*rpl16‐rps3*	GGTTCTCTTTCTTAGTTCCATCTC	CTTCGGATTCTGTCCATTGATAG	1100–1200	58
16	*rpl32‐trnL‐UAG*	CGGTAATGAGCATCCAAAACCAAA	TTTCAAGAATGCCGCCACTCG	800–900	58
17	*ccsA‐ndhD*	GAAGGGTACGAATTCCGCACT	CCTATCCGTTGACAGGGTAGA	300–350	54

The identification rate of cultivars using 17 single markers through phylogenetic tree analysis ranged from 9.09% to 22.73%. Specific useful barcode regions were established based on phylogenetic tree‐based variety identification rates, with the markers *psbI‐trnS‐GCU*, *matK‐rps16*, and *psaA‐ycf3* achieving species identification rates of 22.73% and 20.46%, respectively. However, the overall identification rate was relatively low compared to markers used for other species, as these markers targeted intraspecies variations, resulting in lower identification rates for cultivars.

To evaluate the discriminatory power of existing plant DNA barcodes, we analyzed the species identification rates of *matK*, *rbcL*, and the *trnL‐UAA‐trnF‐GAA* intergenic spacer (Table 3). The results showed that these universal markers provided a low identification rate of approximately 11.36% individually, and even when combined (*matK* + *rbcL* + *trnL‐F*), the total identification rate remained only 18.18%. This highlights the limitations of traditional barcoding markers in resolving closely related species and cultivars within the *Triticum* genus.

**Table 3:** Sequence matching of the hotspot region markers and tree‐based species identification

No.	Region name	Pi	Alignment length (bp)	Identification of ML tree (%)
1	*ccsA*	0.00086	1268	11.36
2	*atpI*	0.00057	825	9.09
3	*matK*	0.00131	1647	11.36
4	*ndhH*	0.00049	936	9.09
5	*psbA*	0.00017	1087	6.82
6	*rpoA*	0.00038	991	6.82
7	*matK‐rps16*	0.00148	1503	20.46
8	*psbI‐trnS‐GCU*	0.00539	256	22.73
9	*atpF‐intron*	0.00012	973	11.36
10	*psaA‐ycf3*	0.00092	759	20.46
11	*trnT‐UGU‐trnL‐UAA*	0.00121	640	9.09
12	*trnL‐UAA‐trnF‐GAA*	0.00135	1110	11.36
13	*petA‐psbJ*	0.00137	933	20.46
14	*psbE‐petL*	0.00095	1271	15.91
15	*rpl16‐rps3*	0.00107	1177	18.18
16	*rpl32‐trnL‐UAG*	0.00586	865	13.64
17	*ccsA‐ndhD*	0.00423	344	9.09
Total combination	*ccsA* (1)*~ccsA‐ndhD* (17)		16 585	50.00

Pi, nucleotide diversity; ML, maximum likelihood.

**Figure 7:**
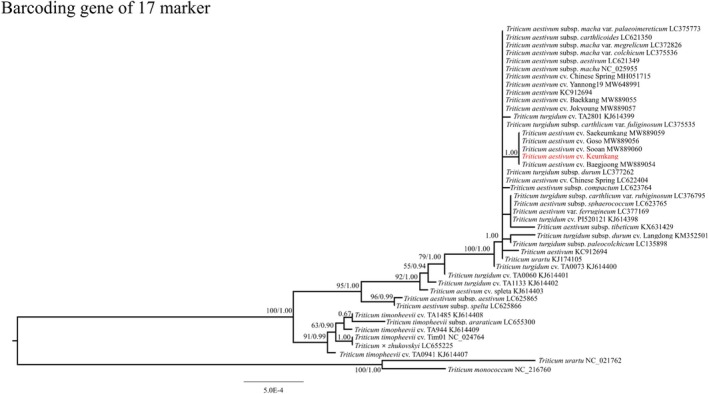
Phylogenetic tree for barcoding region sequence combinations of coding sequences and intergenic region for *Triticum* species.

**Figure 8:**
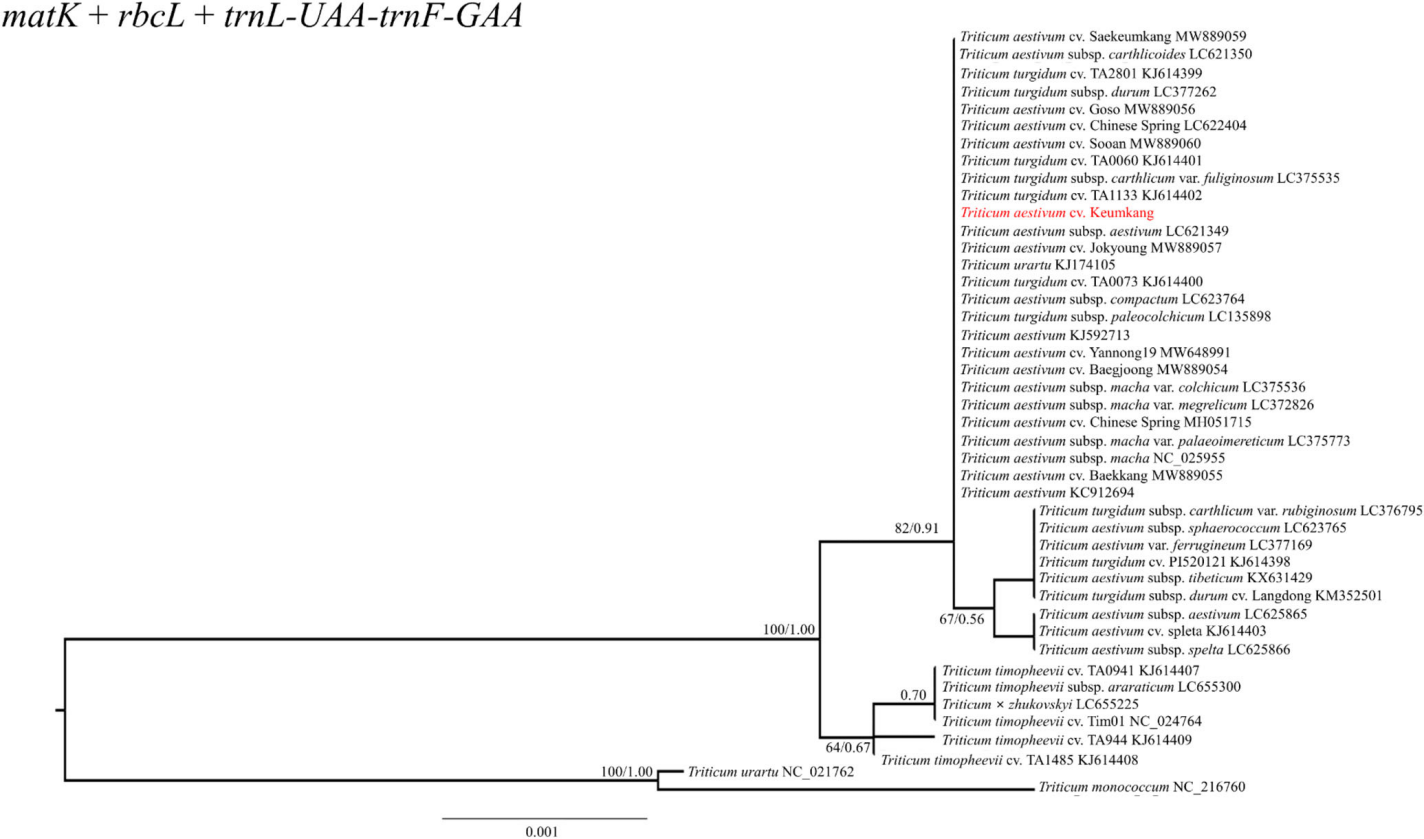
Phylogenetic tree for barcoding region sequence combinations of common barcoding genes of plants for *Triticum* species.

To improve identification, we generated a multi‐locus composite barcode by concatenating the 17 marker alignments. Briefly, each marker was aligned separately across all accessions using MAFFT, the alignments were trimmed to retain only homologous regions and to remove primer flanking segments, and the resulting 17 curated alignments were concatenated in a fixed genomic order to produce a single supermatrix per accession. This concatenation increases the number of informative sites and reduces stochastic error from any single locus, thereby improving cultivar‐level discrimination in phylogenetic reconstruction. The species identification accuracy was significantly enhanced when using the combined dataset. The phylogenetic tree constructed using the concatenated sequences of the 17 newly developed specific barcode markers (CDS and intergenic regions) achieved an identification rate of 50.00%, successfully resolving 22 out of 44 accessions (Fig. 7; Table 3). In contrast, a phylogenetic tree based on the combination of traditional universal barcodes (*matK* + *rbcL* + *trnL‐F*) yielded a much lower identification rate of 18.18%, failing to distinguish most closely related *Triticum* cultivars (Fig. 8; Table 4). These 17 markers were amplified at annealing temperatures between 54 and 58 °C (Supporting Information, Fig. S2).

**Table 4:** Sequence and tree‐based species identification rate of existing barcode markers in the genus *Triticum*

No.	Region name	Length (bp)	Identification of ML tree (%)
1	*matK*	1539	11.36
2	*rbcL*	1434	4.55
3	*trnL‐UAA‐trnF‐GAA*	386	11.36
Total combination	*matK + rbcL + trnL‐UAA‐trnF‐GAA*	3359	18.18

ML, maximum likelihood.

All 17 specific barcode markers were successfully amplified across the tested accessions, and the corresponding PCR products were visualized via gel electrophoresis (Supporting Information, Figs S3–S19). Gel electrophoresis showed a single clear band at the expected size for 16 of the 17 loci across all tested accessions, indicating that the primer binding sites are conserved and that amplification is robust across *Triticum* and *Aegilops*. For *matK‐rps16*, additional faint bands were observed in cultivars 2, 3, 12, 15, and 18, but the expected target band was consistently present and was used for downstream sequence‐based analyses. Importantly, for most loci the amplicon sizes were identical across multiple species, which indicates that large length polymorphisms are uncommon in these intervals and that discriminatory information is primarily derived from nucleotide substitutions and small indels that are not resolvable by agarose gel size separation. In contrast, the *psbE‐petL* intergenic region showed a visibly shorter amplicon in cultivar 11 (*Aegilops uniaristata*), suggesting the presence of a species‐informative length polymorphism in this locus. Overall, therefore, PCR band patterns in the supplementary figures primarily validate amplification specificity and primer universality, whereas species or cultivar discrimination is supported by the sequence alignments and phylogenetic identification rather than by fragment‐length differences alone.

Except for *matK‐rps16*, all 17 markers were amplified as single bands. For *matK‐rps16*, single bands were obtained for most cultivars, but multiple bands were observed in cultivars 2, 3, 12, 15, and 18. Despite this, the target bands for these cultivars were successfully amplified. Additionally, *psbE‐petL* produced a smaller band in cultivar 11 (*Aegilops uniaristata*) compared to other cultivars. Overall, all 17 markers were successfully amplified.

## DISCUSSION

*Triticum aestivum*, a species within the genus *Triticum*, holds significant value as a food crop.^1^ The chloroplast genome plays a crucial role in tracing the origins of crossbreeding, improving breeding crops, and understanding the evolutionary history of food crops.^1^ In this study, the chloroplast genome of *T. aestivum* cv. Keumkang was found to be 135 909 bp in length. When compared to other *T. aestivum* cultivars, including Saekeumkang, Sooan, Baegjoong, Goso, and Jokyoung, the genomes had the same length, suggesting a shared origin of their chloroplast genomes. The chloroplast genome is predominantly maternally inherited,^42^, ^43^, ^44^, ^45^ and thus chloroplast haplotypes primarily reflect maternal lineages. Because chloroplast genomes primarily reflect maternal lineages, chloroplast genome‐based clustering may differ from classifications based on nuclear genomes, particularly in Triticeae, where complex evolutionary histories have been reported.^42^ Although artificial hybridization is widely used in wheat breeding, the small indels and region‐length differences observed among independent GenBank accessions cannot be directly attributed to hybridization without pedigree‐level evidence and are therefore discussed here as accession‐level chloroplast polymorphisms.^46^, ^47^

Despite being the same species, *T. aestivum* accessions KJ592713 and AB042240 exhibited expansion and contraction in the LSC region, along with minor length differences in the IR region. Such variations are likely attributable to minor shifts at the LSC‐IR and SSC‐IR junctions – a common phenomenon leading to modest IR expansion or contraction in closely related chloroplast genomes.^6^ This highlights intraspecific variation in chloroplast genome size within *Triticum*. Given that the breeding history and maternal lineage information are not available for these public accessions, the observed length differences are more conservatively interpreted as standing chloroplast genetic diversity among accessions rather than as hybridization‐induced changes.^48^ Such variations were also found to affect the distribution of long repeats, which may contribute to expansion and contraction dynamics at LSC, SSC, and IR boundaries.^46^, ^47^, ^49^ Notably, for several publicly available accessions in which the canonical quadripartite structure is not explicitly represented in the deposited features, lower gene counts should be interpreted as an artifact of database representation and annotation rather than as evidence of true IR loss or gene copy‐number reduction.^50^

The long repeat distribution in *T. aestivum* cv. Keumkang was similar to that of other *T. aestivum* cultivars, despite minor differences in motif patterns and repeat numbers. Notably, the long repeat distribution patterns of *T. aestivum* cv. Keumkang (AABBDD genome) were consistent with *T. turgidum* subsp. *durum* (AABB genome), suggesting that the chloroplast genome of *T. aestivum* cv. Keumkang may have originated from *T. turgidum* subsp. *durum*.^3^, ^4^, ^42^, ^51^ This is supported by previous studies indicating that *T. aestivum* evolved through hybridization between *T. turgidum* subsp. *durum* and the D genome donor, *A. tauschii*.^48^, ^52^

Common wheat (*T. aestivum*) is a heterohexaploid species with an AABBDD genome.^9^, ^53^, ^54^, ^55^ Genomic studies have identified the AA genome as *T. urartu*, the S genome (ancestral to the B genome) as *A. speltoides*, and the DD genome as *A. tauschii*, while the AABB genome corresponds to *T. turgidum* subsp. *durum*.^3^, ^4^, ^25^, ^30^, ^42^, ^51^ Although the origins of these genomes are well studied, the chloroplast genome's role in this evolutionary history has not been extensively explored.^48^, ^53^ The availability of full chloroplast genome sequences in databases, including both cultivated and wild species, now enables more precise tracing of their maternal lineages and evolutionary history.^25^, ^31^, ^53^, ^54^

In this study, phylogenetic analysis of 70 Poaceae species and cultivars based on their chloroplast genomes positioned *T. aestivum* cv. Keumkang closely with *T. aestivum*, *T. turgidum* subsp. *durum*, and *T. aestivum* cv. Chinese Spring. Keumkang was grouped in the same clade as Saekeumkang, Sooan, Baegjoong, and Goso cultivars, further supporting their shared chloroplast genome origin. Interestingly, *T. urartu* (KJ174105) was misclassified within the *T. aestivum* clade due to morphological identification errors, whereas *T. urartu* (KJ614411) was reliably grouped with *T. monococcum*, consistent with previous findings.^51^ Unlike earlier studies where the S genome (*A. speltoides*) was closely associated with *T. aestivum* based on the *ndhF* gene, our analysis showed that *A. speltoides* formed a distinct clade.^25^, ^30^ This discrepancy may stem from differing evolutionary rates between specific genes and the entire genome.^22^ To accurately trace chloroplast origins, it is essential to use genome‐wide approaches or markers with varying evolutionary rates across different regions.^56^, ^57^

Specific barcode markers are valuable for resolving evolutionary relationships among closely related plants with high resolution.^56^, ^58^, ^59^, ^60^, ^61^ As mutations in intergenic regions occur faster than in coding sequences (CDS),^62^ barcode markers are often developed in intergenic regions. In this study, we selected 17 barcode markers, primarily from intergenic regions, that provided higher resolution for distinguishing the *Triticum* taxa examined here and performed comparably to full chloroplast genome analyses within our dataset. Traditional barcode markers (*matK*, *rbcL*, and *trnL‐UAA‐trnF‐GAA*) were ineffective for discriminating *Triticum* varieties due to their low resolution.

The primers for these 17 specific barcode markers were designed in conserved flanking regions to support broad PCR amplifiability across *Triticum*, whereas the amplified intervals were chosen to contain species‐informative substitutions and indels identified from comparative alignments. However, the discriminatory accuracy for additional cultivars or species not included in this study should be considered provisional and requires empirical validation using expanded reference panels. In particular, because chloroplast markers represent maternal lineages, newly introduced cultivars that share the same chloroplast haplotype may not be fully separable by chloroplast loci alone.^63^, ^64^ Therefore, practical deployment for breeding and authentication should incorporate periodic updating of reference sequences and, when needed, complementary nuclear markers.

## CONCLUSIONS

We successfully determined and characterized the complete chloroplast genome of *T. aestivum* cv. Keumkang. In addition, the complete chloroplast genome sequences of 44 *Triticum* species and cultivars were analyzed and compared. Significant genetic mutations were observed across the *Triticum* genus, with *T. aestivum* cv. Keumkang being distinguishable from the commonly studied *T. aestivum* cv. Chinese Spring. Nucleotide analysis identified six coding sequences (CDS) and 11 intergenic regions with high polymorphism. Traditional barcoding markers (*matK*, *rbcL*, and *trnL‐F*) showed a low identification rate of approximately 18% when analyzed using an ML tree. However, the newly developed combination of 17 markers demonstrated an identification rate nearly twice as high, highlighting their effectiveness as barcoding tools. Primers for the 17 combined markers were designed from conserved regions to ensure amplification across the *Triticum* genus; however, their cultivar or species discrimination performance for newly added germplasm should be verified by expanding the reference dataset and, if necessary, integrating complementary nuclear markers. PCR amplification of these markers in *T. aestivum* cv. Keumkang was successful for all 17 markers, confirming their utility for species identification and genetic studies within the genus.

## AUTHOR CONTRIBUTIONS

Conceptualization: Kang‐Rae Kim, Jin‐Hyun Kim, Changhyun Choi. Methodology: Kang‐Rae Kim. Formal analysis and investigation: Kang‐Rae Kim, Hwa Jin Jung, Jung Sun Kim. Writing – original draft preparation: Kang‐Rae Kim. Writing – review and editing: Kang‐Rae Kim, Jin‐Hyun Kim, Changhyun Choi, Jung Sun Kim, Myung‐Hee Kim, DaHye Jeon. Funding acquisition: Jin‐Hyun Kim. Resources: Jin‐Hyun Kim, Hwa Jin Jung. Supervision: Jin‐Hyun Kim.

## FUNDING INFORMATION

This work was carried out with the support of ‘Cooperative Research Program for Agriculture Science and Technology Development (Project No. PJ017444)’ Rural Development Administration, Republic of Korea.

## CONFLICT OF INTEREST

The authors declare no conflict of interest. The funders had no role in the design of the study; in the collection, analyses, or interpretation of data; in the writing of the manuscript; or in the decision to publish the results.

## Supporting information


**Table S1.** List of species information used for comparative chloroplast genome of *Triticum* and Poaceae
Table S2. Detailed information of the 18 *Triticum* and *Aegilops* accessions used for PCR validation, including IT numbers and origins
Table S3. List of genes in *Triticum aestivum* cv. Keumkang
Table S4. Comparative information summary of the cp genome of the species *Triticum* and *Aegilops* genus
Table S5. Distribution information summary by species according to the long repeat of *Triticum*
Table S6. SSR region information of the species cp genome of the genus *Triticum*
Figure S1. Bayesian inference (BI) and maximum likelihood (ML) phylogenetic tree of 17 specific barcode regions markers.
Figure S2. PCR amplification products of specific barcoding markers 1–17. M: DNA ladder, 1: *ccsA*, 2: *atpI*, 3: *matK*, 4: *ndhH*, 5: *psbA*, 6: *rpoA*, 7: *matK‐rps16*, 8: *psbI‐trnS‐GCU*, 9: *atpF‐intron*, 10: *psaA‐ycf3*, 11: *trnT‐UGU‐trnL‐UAA*, 12: *trnL‐UAA‐trnF‐GAA*, 13: *petA‐psbJ*, 14: *psbE‐petL*, 15: *rpl16‐rps3*, 16: *rpl32‐trnL‐UAG*, 17: *ccsA‐ndhD*.
Figure S3. PCR amplification products of cultivar 1–18 for the specific barcoding marker *ccsA* gene.
Figure S4. PCR amplification products of cultivar 1–18 for the specific barcoding marker *atpI* gene.
Figure S5. PCR amplification products of cultivar 1–18 for the specific barcoding marker *matK* gene.
Figure S6. PCR amplification products of cultivar 1–18 for the specific barcoding marker *ndhH* gene.
Figure S7. PCR amplification products of cultivar 1–18 for the specific barcoding marker *psbA* gene.
Figure S8. PCR amplification products of cultivar 1–18 for the specific barcoding marker *rpoA* gene.
Figure S9. PCR amplification products of cultivar 1–18 for the specific barcoding marker *matK‐rps16*.
Figure S10. PCR amplification products of cultivar 1–18 for the specific barcoding marker *psbl‐trnS‐GCU*.
Figure S11. PCR amplification products of cultivar 1–18 for the specific barcoding marker *atpF‐intron*.
Figure S12. PCR amplification products of cultivar 1–18 for the specific barcoding marker *psaA‐ycf3*.
Figure S13. PCR amplification products of cultivar 1–18 for the specific barcoding marker *trnT‐UGU‐trnL‐UAA*.
Figure S14. PCR amplification products of cultivar 1–18 for the specific barcoding marker *trnL‐UAA‐trnF‐GAA*.
Figure S15. PCR amplification products of cultivar 1–18 for the specific barcoding marker *petA‐psbJ*.
Figure S16. PCR amplification products of cultivar 1–18 for the specific barcoding marker *psbE‐petL*.
Figure S17. PCR amplification products of cultivar 1–18 for the specific barcoding marker *rpl16‐rps3*.
Figure S18. PCR amplification products of cultivar 1–18 for the specific barcoding marker *rpl32‐trnL‐UAG*.
Figure S19. PCR amplification products of cultivar 1–18 for the specific barcoding marker *ccsA‐ndhD*.

## Data Availability

The chloroplast genome sequence data that support the findings of this study are openly available from NCBI GenBank (https://www.ncbi.nlm.nih.gov/) under the accession number PP829256. The data that support the findings of this study are available on request from the author.
