# Supplementary material for: Complete chloroplast genome of Triticum aestivum cultivar ‘Keumkang’ from Korea (Poaceae) and comparative chloroplast genomes of the members of the Triticum genus
Source: J Sci Food Agric. 2026 Feb 11;106(6):3732–44. doi: 10.1002/jsfa.70489 (PMC12988713; doi:10.1002/jsfa.70489)
Supplement: Supplementary file 1 — Table S1. List of species information used for comparative chloroplast genome of Triticum and Poaceae Table S2. Detailed information of the 18 Triticum and Aegilops accessions used for PCR validation, including IT numbers and origins Table S3. List of genes in Triticum aestivum cv. Keumkang Table S4. Comparative information summary of the cp genome of the species Triticum and Aegilops genus Table S5. Distribution information summary by species according to the long repeat of Triticum Table S6. SSR region information of the species cp genome of the genus Triticum Figure S1. Bayesian inference (BI) and maximum likelihood (ML) phylogenetic tree of 17 specific barcode regions markers. Figure S2. PCR amplification products of specific barcoding markers 1–17. M: DNA ladder, 1: ccsA, 2: atpI, 3: matK, 4: ndhH, 5: psbA, 6: rpoA, 7: matK‐rps16, 8: psbI‐trnS‐GCU, 9: atpF‐intron, 10: psaA‐ycf3, 11: trnT‐UGU‐trnL‐UAA, 12: trnL‐UAA‐trnF‐GAA, 13: petA‐psbJ, 14: psbE‐petL, 15: rpl16‐rps3, 16: rpl32‐trnL‐UAG, 17: ccsA‐ndhD. Figure S3. PCR amplification products of cultivar 1–18 for the specific barcoding marker ccsA gene. Figure S4. PCR amplification products of cultivar 1–18 for the specific barcoding marker atpI gene. Figure S5. PCR amplification products of cultivar 1–18 for the specific barcoding marker matK gene. Figure S6. PCR amplification products of cultivar 1–18 for the specific barcoding marker ndhH gene. Figure S7. PCR amplification products of cultivar 1–18 for the specific barcoding marker psbA gene. Figure S8. PCR amplification products of cultivar 1–18 for the specific barcoding marker rpoA gene. Figure S9. PCR amplification products of cultivar 1–18 for the specific barcoding marker matK‐rps16. Figure S10. PCR amplification products of cultivar 1–18 for the specific barcoding marker psbl‐trnS‐GCU. Figure S11. PCR amplification products of cultivar 1–18 for the specific barcoding marker atpF‐intron. Figure S12. PCR amplification products of cultivar 1–18 for the spe [file JSFA-106-3732-s001.docx]

**Table S1.** List of species information used for comparative chloroplast genome of *Triticum* and Poaceae

| **No.** | **Science name** | **GenBank accession number** |
| --- | --- | --- |
| 1 | *Triticum aestivum* cv. Keumkang | PP829256 |
| 2 | *Triticum aestivum* cv. Saekeumkang | MW889059 |
| 3 | *Triticum aestivum* cv. Sooan | MW889060 |
| 4 | *Triticum aestivum* cv. Baegjoong | MW889054 |
| 5 | *Triticum aestivum* cv. Baekkang | MW889055 |
| 6 | *Triticum aestivum* cv. Goso | MW889056 |
| 7 | *Triticum aestivum* cv. Jokyoung | MW889057 |
| 8 | *Triticum aestivum* cv. Chinese Spring | LC622404 |
| 9 | *Triticum aestivum* cv. Chinese Spring | MH051715 |
| 10 | *Triticum aestivum* subsp. *carthlicoides* | LC621350 |
| 11 | *Triticum aestivum* subsp. *tibeticum* | KX631429 |
| 12 | *Triticum aestivum* cv. spleta | KJ614403 |
| 13 | *Triticum aestivum* cv. Yannong19 | MW648991 |
| 14 | *Triticum aestivum* var. *ferrugineum* | LC377169 |
| 15 | *Triticum aestivum* subsp. *aestivum* var. *vavilovii* | LC621349 |
| 16 | *Triticum aestivum* subsp. *aestivum* var. *vavilovii* | LC625865 |
| 17 | *Triticum aestivum* | AB042240 |
| 18 | *Triticum aestivum* | KC912694 |
| 19 | *Triticum aestivum* | KJ592713 |
| 20 | *Triticum turgidum* subsp. *carthlicum* var. *fuliginosum* | LC375535 |
| 21 | *Triticum turgidum* subsp. *carthlicum* var. *rubiginosum* | LC376795 |
| 22 | *Triticum aestivum* subsp. *compactum* | LC623764 |
| 23 | *Triticum aestivum* subsp. *macha* var. *colchicum* | LC375536 |
| 24 | *Triticum aestivum* subsp. *macha* var. *megrelicum* | LC372826 |
| 25 | *Triticum aestivum* subsp. *macha* var. *palaeoimereticum* | LC375773 |
| 26 | *Triticum aestivum* subsp. *macha* | NC_025955 |
| 27 | *Triticum monococcum* | KY636171 |
| 28 | *Triticum monococcum* | MG958558 |
| 29 | *Triticum aestivum* subsp. *spelta* | LC625866 |
| 30 | *Triticum aestivum* subsp. *sphaerococcum* | LC623765 |
| 31 | *Triticum timopheevii* cv. TA0941 | KJ614407 |
| 32 | *Triticum timopheevii* cv. TA944 | KJ614409 |
| 33 | *Triticum timopheevii* cv. TA1485 | KJ614408 |
| 34 | *Triticum timopheevii* cv. Tim01 | NC_024764 |
| 35 | *Triticum turgidum* cv. PI520121 | KJ614398 |
| 36 | *Triticum turgidum* cv. TA0060 | KJ614401 |
| 37 | *Triticum turgidum* cv. TA0073 | KJ614400 |
| 38 | *Triticum turgidum* cv. TA1133 | KJ614402 |
| 39 | *Triticum turgidum* cv. TA2801 | KJ614399 |
| 40 | *Triticum turgidum* subsp. *durum* cv. Langdon | KM352501 |
| 41 | *Triticum turgidum* subsp. *durum* | LC377262 |
| 42 | *Triticum turgidum* subsp. *paleocolchicum* | LC135898 |
| 43 | *Triticum urartu* | KJ174105 |
| 44 | *Triticum urartu* | NC_021762 |
| 45 | *Triticum urartu* | KJ614411 |
| 46 | *Triticum × zhukovskyi* | LC655225 |
| 47 | *Aegilops bicornis* cv. Clae57 | NC_024831 |
| 48 | *Aegilops cylindrica* | NC_023096 |
| 49 | *Aegilops geniculata* | NC_023097 |
| 50 | *Aegilops kotschyi* cv. TA1980 | NC_024832 |
| 51 | *Aegilops longissima* cv. TA1924 | NC_024830 |
| 52 | *Aegilops searsii* cv. TA1926 | KJ614413 |
| 53 | *Aegilops sharonensis* cv. TA1996 | KJ614417 |
| 54 | *Aegilops speltoides* var. *aucheri* | OR936055 |
| 55 | *Aegilops speltoides* var. *ligustica* cv. AE918 | KJ614404 |
| 56 | *Aegilops speltoides* var. *speltoides* cv. PI487232 | KJ614406 |
| 57 | *Aegilops speltoides* | JQ740834 |
| 58 | *Aegilops tauschii* | NC_022133 |
| 59 | *Aegilops tauschii* | MN223978 |
| 60 | *Aegilops umbellulata* | MG958547 |
| 61 | *Australopyrum retrofractum* | NC_043840 |
| 62 | *Crithopsis delileana* | MH285849 |
| 63 | *Henrardia persica* | MH285853 |
| 64 | *Leymus cinereus* | NC_068544 |
| 65 | *Leymus coreanus* | NC_068542 |
| 66 | *Leymus mollis* | NC_063961 |
| 67 | *Leymus triticoides* | MZ595310 |
| 68 | *Secale cereale* subsp. *segetale* | MZ507427 |
| 69 | *Secale strictum* subsp. *anatolicum* | OQ700976 |
| 70 | *Aegilops tauschii* | SRR17194743 |

**Table S2.** Detailed information of the 18 *Triticum* and *Aegilops* accessions used for PCR validation, including IT numbers and origins.

| No. | Scientific name | IT number | Resource | Common name | Resource name |
| --- | --- | --- | --- | --- | --- |
| 1 | *Triticum aestivum* subsp. *aestivum* | 172226 | UNK | Wheat | Chinese spring |
| 2 | *Triticum urartu* | 177184 | TUR | Red wild einkorn | WIR58504 |
| 3 | *Triticum urartu* | 177185 | ARM | Red wild einkorn | WIR33871 |
| 4 | *Triticum aestivum* subsp. *aestivum* | 185166 | UNK | Wheat | Chinese spring |
| 5 | *Triticum urartu* | 202089 | UNK | Red wild einkorn | EC374947 |
| 6 | *Aegilops biuncialis* | 204618 | TUR | Wheat | CWI49020 |
| 7 | *Triticum aestivum* subsp. *aestivum* | 267172 | USA | Wheat | MASSEY |
| 8 | *Triticum turgidum* subsp. *durum* | 269514 | MNG | Durum wheat | HB616 |
| 9 | *Triticum turgidum* subsp. *durum* | 269515 | MNG | Durum wheat | HB757 |
| 10 | *Triticum aestivum* subsp. *aestivum* | 293620 | CHN | Wheat | yv 99-67 |
| 11 | *Aegilops uniaristata* | 302223 | GRC | Wheat | KU-5820 |
| 12 | *Aegilops ventricosa* | 302224 | FRA | Wheat | AE1197 |
| 13 | *Triticum aestivum* subsp. *aestivum* | 324662 | TUR | Wheat | PI 537976 |
| 14 | *Triticum turgidum* subsp. *durum* | 330427 | CAN | Durum wheat | Wakooma |
| 15 | *Triticum urartu* | 330603 | SYR | Red wild einkorn | PI 487268 |
| 16 | *Triticum turgidum* subsp. *durum* | 340493 | CAN | Durum wheat | DT367 |
| 17 | *Triticum aestivum* subsp. aestivum | 341831 | KOR | Wheat | 326-6 ("02-"03 HR) |
| 18 | *Aegilops cylindrica* | 352075 | TJK | Wheat | TJK 32-5 |

**Table S3**. List of genes in the *Triticum* *aestivum* cv. Keumkang

| Gene category |  | Gene name |
| --- | --- | --- |
| Photosynthesis | Photosystem Ⅰ | *psaA*, *B*, *C*, *I*, *J* |
|  | Photosystem Ⅱ | *psbA*, *B*, *C*, *D*, *E*, *F*, *H*, *I*, *J*, *K*, *L*, *M*, *N*, *T*, *Z* |
|  | Cytochrome b/f complex | *petA*, *B*, *D*, *G*, *L*, *N* |
|  | ATP synthase | *atpA*, *B*, *E*, *F*, *H*, *I* |
|  | NADH dehydrogenase | *ndhA*, *B* (×2), *C*, *D*, *E*, *F*, *G*, *H*, *I*, *J*, *K* |
|  | Rubisco | *rbcL* |
| Self-replication | Transfer RNAs | *trnA-UGC* (×2), *C-GCA*, *D-GUC*, *E-UUC*, *F-GAA*, *trnfM-CAU*, *G-GCC* (×2), *G-UCC*, *H-GUG* (×2), *I-GAU* (×2), *K-UUU*, *L-CAA* (×2), *L-UAA*, *L-UAG*, *M-CAU* (×4), *N-GUU* (×2), *P-UGG*, *Q-UGG*, *R-ACG* (×2), *R-UCU*, *S-GCU*, *S-GGA*, *S-UGA*, *T-GGU*, *T-UGU*, *V-GAC* (×2), *V-UAC*, *W-CCA*, *Y-GUA* |
|  | Ribosomal RNAs | *rrn 16* (×2), *23* (×2), *4.5* (×2), *5* (×2) |
|  | RNA polymerase subunits | *rpoA*, *B*, *C1*, *C2* |
|  | Large subunit ribosomal proteins | *rpl2* (×2), *14*, *16*, *20*, *22*, *23* (×3), *32*, *33*, *36* |
|  | Small subunit ribosomal proteins | *rps2*, *3*, *4*, *7* (×2), *8*, *11*, *12* (×2), *14*, *15* (×2), *16*, *18*, *19* (×2) |
| Other genes | Maturase | *matK* |
|  | Envelope membrane protein | *cemA* |
|  | Proteins of unknown function | *ycf2* (×2), *ycf3*, *ycf4* |
|  | c-type cytochrome synthesis gene | *ccsA* |
|  | Pseudogenes | *infA* |
|  |  |  |

**Table S4.** Comparative information summary of the cp genome of the species *Triticum* and *Aegilops* genus

| Feature | NCBI accession no. | Total genome size (bp) | Large single copy (bp) | Small single copy (bp) | Inverted repeat region (bp) | Total number of genes | Protein-coding genes | tRNA genes | rRNA genes |
| --- | --- | --- | --- | --- | --- | --- | --- | --- | --- |
| *Triticum aestivum* cv. Keumkang |  | 135,909 | 80,014 | 12,791 | 21,552 | 132 | 85 | 39 | 8 |
| *Triticum aestivum* cv. Saekeumkang | MW889059 | 135,909 | 80,014 | 12,791 | 21,552 | 132 | 85 | 39 | 8 |
| *Triticum aestivum* cv. Sooan | MW889060 | 135,909 | 80,014 | 12,791 | 21,552 | 132 | 85 | 39 | 8 |
| *Triticum aestivum* cv. Baegjoong | MW889054 | 135,909 | 80,014 | 12,791 | 21,552 | 132 | 85 | 39 | 8 |
| *Triticum aestivum* cv. Baekkang | MW889055 | 135,900 | 80,005 | 12,791 | 21,552 | 132 | 85 | 39 | 8 |
| *Triticum aestivum* cv. Goso | MW889056 | 135,909 | 80,014 | 12,791 | 21,552 | 132 | 85 | 39 | 8 |
| *Triticum aestivum* cv. Jokyoung | MW889057 | 135,909 | 80,014 | 12,791 | 21,552 | 132 | 85 | 39 | 8 |
| *Triticum aestivum* cv. Chinese Spring | LC622404 | 135,899 | 80,004 | 12,791 | 21,552 | 132 | 85 | 39 | 8 |
| *Triticum aestivum* cv. Chinese Spring | MH051715 | 135,905 | 80,010 | 12,791 | 21,552 | 132 | 85 | 39 | 8 |
| *Triticum aestivum carthlicoides* | LC621350 | 135,898 | 80,003 | 12,791 | 21,552 | 132 | 85 | 39 | 8 |
| *Triticum aestivum* subsp. *tibeticum* | KX631429 | 135,897 | 80,002 | 12,791 | 21,552 | 132 | 85 | 39 | 8 |
| *Triticum aestivum* cv. spleta | KJ614403 | 135,919 | 80,045 | 12,792 | 21,541 | 132 | 85 | 39 | 8 |
| *Triticum aestivum* cv. Yannong | MW648991 | 135,900 | 80,005 | 12,791 | 21,552 | 132 | 85 | 39 | 8 |
| *Triticum aestivum* var. *ferrugineum* | LC377169 | 135,897 | 80,002 | 12,791 | 21,552 | 132 | 85 | 39 | 8 |
| *Triticum aestivum* var. *vavilovii* | LC621349 | 135,898 | 80,003 | 12,791 | 21,552 | 132 | 85 | 39 | 8 |
| *Triticum aestivum* var. *vavilovii* | LC625865 | 135,944 | 80,051 | 12,791 | 21,551 | 132 | 85 | 39 | 8 |
| *Triticum aestivum* | AB042240 | 134,545 | 80,348 | 12,791 | 20,703 | 132 | 85 | 39 | 8 |
| *Triticum aestivum* | KC912694 | 114,984 | - | - | - | 113 | 79 | 30 | 4 |
| *Triticum aestivum* | KJ592713 | 133,873 | 79,936 | 12,791 | 20,573 | 132 | 85 | 39 | 8 |
| *Triticum carthlicum* var. *fuliginosum* | LC375535 | 135,897 | 80,002 | 12,791 | 21,552 | 132 | 85 | 39 | 8 |
| *Triticum carthlicum* var. *rubiginosum* | LC376795 | 135,898 | 80,003 | 12,791 | 21,552 | 132 | 85 | 39 | 8 |
| *Triticum compactum* | LC623764 | 135,897 | 80,002 | 12,791 | 21,552 | 132 | 85 | 39 | 8 |
| *Triticum macha* var. *colchicum* | LC375536 | 135,899 | 80,004 | 12,791 | 21,552 | 132 | 85 | 39 | 8 |
| *Triticum macha* var. *megrelicum* | LC372826 | 135,899 | 80,004 | 12,791 | 21,552 | 132 | 85 | 39 | 8 |
| *Triticum macha* var. *palaeoimereticum* | LC375773 | 135,899 | 80,004 | 12,791 | 21,552 | 132 | 85 | 39 | 8 |
| *Triticum macha* | NC_025955 | 135,899 | 80,004 | 12,791 | 21,552 | 132 | 85 | 39 | 8 |
| *Triticum monococcum* | KY636171 | 116,399 | - | - | - | 113 | 79 | 30 | 4 |
| *Triticum monococcum* | CN052948 | 136,758 | 80,986 | 12,806 | 21,482 | 132 | 85 | 39 | 8 |
| *Triticum spelta* | LC625866 | 135,948 | 80,052 | 12,792 | 21,552 | 132 | 85 | 39 | 8 |
| *Triticum sphaerococcum* | LC623765 | 135,916 | 80,021 | 12,791 | 21,552 | 132 | 85 | 39 | 8 |
| *Triticum timopheevii* cv. TA0941 | KJ614407 | 136,074 | 80,179 | 12,789 | 21,553 | 132 | 85 | 39 | 8 |
| *Triticum timopheevii* cv. TA944 | KJ614409 | 136,124 | 80,229 | 12,789 | 21,553 | 132 | 85 | 39 | 8 |
| *Triticum timopheevii* cv. TA1485 | KJ614408 | 136,119 | 80,224 | 12,789 | 21,553 | 132 | 85 | 39 | 8 |
| *Triticum timopheevii* cv. Tim01 | NC024764 | 136,157 | 80,256 | 12,795 | 21,553 | 132 | 85 | 39 | 8 |
| *Triticum turgidum* cv. PI520121 | KJ614398 | 135,836 | 79,962 | 12,792 | 21,541 | 132 | 85 | 39 | 8 |
| *Triticum turgidum* cv. TA0060 | KJ614401 | 135,926 | 80,052 | 12,792 | 21,541 | 132 | 85 | 39 | 8 |
| *Triticum turgidum* cv. TA0073 | KJ614400 | 135,865 | 79,991 | 12,792 | 21,541 | 132 | 85 | 39 | 8 |
| *Triticum turgidum* cv. TA1133 | KJ614402 | 135,889 | 80,015 | 12,792 | 21,541 | 132 | 85 | 39 | 8 |
| *Triticum turgidum* cv. TA2801 | KJ614399 | 135,835 | 79,961 | 12,792 | 21,541 | 132 | 85 | 39 | 8 |
| *Triticum turgidum* subsp. *durum* cv. Langdon | KM352501 | 135,948 | 80,000 | 12,818 | 21,565 | 132 | 85 | 39 | 8 |
| *Triticum turgidum* subsp. *durum* | LC377262 | 135,898 | 80,003 | 12,791 | 21,552 | 132 | 85 | 39 | 8 |
| *Triticum turgidum* subsp. *paleocolchicum* | LC135898 | 135,898 | 80,003 | 12,791 | 21,552 | 132 | 85 | 39 | 8 |
| *Triticum urartu* | KJ174105 | 135,900 | 80,005 | 12,791 | 21,552 | 132 | 85 | 39 | 8 |
| *Triticum urartu* | KJ614411 | 136,865 | 80,947 | 12,824 | 21,547 | 132 | 85 | 39 | 8 |
| *Triticum urartu* | NC_021762 | 115,773 | - | - | - | 92 | 60 | 28 | 4 |
| *Triticum zhukovskyi* | LC655225 | 136,157 | 80,256 | 12,795 | 21,553 | 132 | 85 | 39 | 8 |
| *Aegilops bicornis* cv. Clae57 | NC_024831 | 137,074 | 81,002 | 12,775 | 21,542 | 132 | 85 | 39 | 8 |
| *Aegilops cylindrica* | NC_023096 | 113,490 | - | - | - | 111 | 77 | 30 | 4 |
| *Aegilops geniculata* | NC_023097 | 113,893 | - | - | - | 114 | 79 | 31 | 4 |
| *Aegilops kotschyi* cv. TA1980 | NC_024832 | 136,868 | 81,008 | 12,776 | 21,542 | 132 | 85 | 39 | 8 |
| *Aegilops longissima* cv. TA1924 | NC_024830 | 136,875 | 81,013 | 12,778 | 21,542 | 132 | 85 | 39 | 8 |
| *Aegilops searsii* cv. TA1926 | KJ614413 | 136,870 | 81,004 | 12,782 | 21,542 | 132 | 85 | 39 | 8 |
| *Aegilops sharonensis* cv. TA1996 | KJ614417 | 136,875 | 81,012 | 12,779 | 21,542 | 132 | 85 | 39 | 8 |
| *Aegilops speltoides* var. *aucheri* | OR936055 | 135,666 | 79,775 | 12,787 | 21,552 | 132 | 85 | 39 | 8 |
| *Aegilops speltoides* var. *ligustica* cv. AE918 | KJ614404 | 135,660 | 79,767 | 12,787 | 21,553 | 132 | 85 | 39 | 8 |
| *Aegilops speltoides* var. *speltoides* cv. PI487232 | KJ614406 | 135,652 | 79,766 | 12,780 | 21,553 | 132 | 85 | 39 | 8 |
| *Aegilops speltoides* | JQ740834 | 114,112 | - | - | - | 111 | 77 | 29 | 5 |
| *Aegilops tauschii* | NC_022133 | 114,112 | - | - | - | 114 | 80 | 30 | 4 |
| *Aegilops tauschii* | MN223978 | 136,009 | 80,142 | 12,771 | 21,548 | 132 | 85 | 39 | 8 |
| *Aegilops umbellulata* | MG958547 | 136,743 | 80,988 | 12,779 | 21,488 | 132 | 85 | 39 | 8 |

**Table S5.** Distribution information summary by species according to the long repeat of *Triticum*

| Repeat length | 30 | 33 | 35 | 51 | 52 | 79 | 89 | 111 | 136 | 140 | 145 | 174 | 183 | 286 |
| --- | --- | --- | --- | --- | --- | --- | --- | --- | --- | --- | --- | --- | --- | --- |
| *T. aestivum* cv. Keumkang | 6 |  |  |  |  |  |  | 3 |  |  |  | 3 |  |  |
| *T. aestivum* cv. Saekeumkang | 6 |  |  |  |  |  |  | 3 |  |  |  | 3 |  |  |
| *T. aestivum* cv. Sooan | 6 |  |  |  |  |  |  | 3 |  |  |  | 3 |  |  |
| *T. aestivum* cv. Jokyoung | 6 |  |  |  |  |  |  | 3 |  |  |  | 3 |  |  |
| *T. aestivum* cv. Goso | 6 |  |  |  |  |  |  | 3 |  |  |  | 3 |  |  |
| *T. aestivum* cv. Baekkang | 6 |  |  |  |  |  |  | 3 |  |  |  | 3 |  |  |
| *T. aestivum* cv. Baegjoong | 6 |  |  |  |  |  |  | 3 |  |  |  | 3 |  |  |
| *T. aestivum* cv. spleta | 6 |  |  |  |  |  |  |  |  |  |  |  |  | 3 |
| *T. aestivum* cv. Yannong | 6 |  |  |  |  |  |  |  |  |  |  |  |  | 3 |
| *T. aestivum* cv. Chinese Spring LC622404 | 6 |  |  |  |  |  |  | 3 |  |  |  | 3 |  |  |
| *T. aestivum* cv. Chinese Spring MH051715 | 6 |  |  |  |  |  |  | 3 |  |  |  | 3 |  |  |
| *T. aestivum* subsp. *carthlicoides* | 6 |  |  |  |  |  |  | 3 |  |  |  | 3 |  |  |
| *T. aestivum* subsp. *tibeticum* | 6 |  |  |  |  |  |  |  |  |  |  |  |  | 3 |
| *T. aestivum* var. *ferrugineum* | 6 |  |  |  |  |  |  | 3 |  |  |  | 3 |  |  |
| *T. aestivum* var. *vavilovii* LC621349 | 6 |  |  |  |  |  |  | 3 |  |  |  | 3 |  |  |
| *T. aestivum* var. *vavilovii* LC625865 | 2 |  |  |  |  |  |  |  |  |  |  |  |  | 3 |
| *T. aestivum* AB042240 | 2 |  | 4 |  |  |  | 3 |  | 3 |  |  |  |  |  |
| *T. aestivum* KC912694 | 4 |  |  | 2 |  |  |  | 2 |  |  |  | 3 |  |  |
| *T. aestivum* KJ592713 | 6 |  |  |  | 4 |  |  |  |  | 3 | 3 |  |  |  |
| *T. carthlicum* var. *fuliginosum* | 6 |  |  |  |  |  |  | 3 |  |  |  | 3 |  |  |
| *T. carthlicum* var. *rubiginosum* | 6 |  |  |  |  |  |  | 3 |  |  |  | 3 |  |  |
| *T. compactum* | 6 |  |  |  |  |  |  | 3 |  |  |  | 3 |  |  |
| *T. macha* var. *colchicum* | 6 |  |  |  |  |  |  | 3 |  |  |  | 3 |  |  |
| *T. macha* var. *megrelicum* | 6 |  |  |  |  |  |  |  |  |  |  |  |  | 3 |
| *T. macha* var. *palaeoimereticum* | 6 |  |  |  |  |  |  | 3 |  |  |  | 3 |  |  |
| *T. macha* NC_025955 | 6 |  |  |  |  |  |  | 3 |  |  |  | 3 |  |  |
| *T. monococcum* cv. CN052948 | 6 | 2 |  |  |  |  |  |  |  |  |  |  |  | 3 |
| *T. monococcum* | 4 | 2 |  |  |  |  |  |  |  |  |  |  |  | 2 |
| *T. spelta* | 6 |  |  |  |  |  |  |  |  |  |  |  |  | 3 |
| *T. sphaerococcum* | 6 |  |  |  |  |  |  | 3 |  |  |  | 3 |  |  |
| *T. timopheevii* cv. TA0941 | 6 |  |  |  |  | 3 |  |  |  |  |  |  | 3 |  |
| *T. timopheevii* cv. TA944 | 6 |  |  |  |  | 3 |  |  |  |  |  |  | 3 |  |
| *T. timopheevii* cv. TA1485 | 6 |  |  |  |  |  |  |  |  |  |  |  |  |  |
| *T. timopheevii* cv. Tim01 | 6 |  |  |  | 2 | 3 |  |  |  |  |  |  | 3 |  |
| *T. turgidum* cv. PI520121 | 6 |  |  |  |  |  |  |  |  |  |  |  |  | 3 |
| *T. turgidum* cv. TA0060 | 6 |  |  |  |  |  |  |  |  |  |  |  |  | 3 |
| *T. turgidum* cv. TA0073 | 6 |  |  |  |  |  |  | 3 |  |  |  | 3 |  |  |
| *T. turgidum* cv. TA1133 | 6 |  |  |  |  |  |  |  |  |  |  |  |  | 3 |
| *T. turgidum* cv. TA2801 | 6 |  |  |  |  |  |  | 3 |  |  |  | 3 |  |  |
| *T. turgidum* subsp. *durum* cv. Langdon | 5 |  | 4 |  |  |  |  |  |  |  |  |  |  | 3 |
| *T. turgidum* subsp. *durum* | 6 |  |  |  |  |  |  | 3 |  |  |  | 3 |  |  |
| *T. turgidum* subsp. *paleocolchicum* | 6 |  |  |  |  |  |  | 3 |  |  |  | 3 |  |  |
| *T. urartu* KJ174105 | 6 |  |  |  |  |  |  | 3 |  |  |  | 3 |  |  |
| *T. urartu* NC_021762 | 4 |  |  |  |  |  |  |  |  |  |  |  |  |  |
| *T. zhukovskyi* | 6 |  |  |  | 2 | 3 |  |  |  |  |  |  | 3 |  |

**Table S6.** SSR region information of the species cp genome of the genus *Triticum*

| Species | Region | Mono- | Di- | Tri- | Tetra- | Penta- | Hexa- | Total |
| --- | --- | --- | --- | --- | --- | --- | --- | --- |
| *T. aestivum* cv. Keumkang | LSC |  |  | 22 | 5 | 4 | 2 | 33 |
|  | SSC |  |  | 1 |  |  |  | 1 |
|  | IR |  |  | 6 | 1 |  |  | 7 |
| *T. aestivum* cv. Saekeumkang | LSC |  |  | 22 | 5 | 4 | 2 | 33 |
|  | SSC |  |  | 1 |  |  |  | 1 |
|  | IR |  |  | 6 | 1 |  |  | 7 |
| *T. aestivum* cv. Sooan | LSC |  |  | 22 | 5 | 4 | 2 | 33 |
|  | SSC |  |  | 1 |  |  |  | 1 |
|  | IR |  |  | 6 | 1 |  |  | 7 |
| *T. aestivum* cv. Jokyoung | LSC |  |  | 22 | 5 | 3 | 2 | 32 |
|  | SSC |  |  | 1 |  |  |  | 1 |
|  | IR |  |  | 6 | 1 |  |  | 7 |
| *T. aestivum* cv. Goso | LSC |  |  | 22 | 5 | 4 | 2 | 33 |
|  | SSC |  |  | 1 |  |  |  | 1 |
|  | IR |  |  | 6 | 1 |  |  | 7 |
| *T. aestivum* cv. Baekkang | LSC |  |  | 22 | 5 | 3 | 2 | 32 |
|  | SSC |  |  | 1 |  |  |  | 1 |
|  | IR |  |  | 6 | 1 |  |  | 7 |
| *T. aestivum* cv. Baegjoong | LSC |  |  | 22 | 5 | 4 | 2 | 33 |
|  | SSC |  |  | 1 |  |  |  | 1 |
|  | IR |  |  | 6 | 1 |  |  | 7 |
| *T. aestivum* cv. spleta | LSC |  |  | 22 | 5 | 3 | 2 | 32 |
|  | SSC |  |  | 1 |  |  |  | 1 |
|  | IR |  |  | 7 | 1 |  |  | 7 |
| *T. aestivum* cv. Yannong | LSC |  |  | 22 | 5 | 3 | 2 | 32 |
|  | SSC |  |  | 1 |  |  |  | 1 |
|  | IR |  |  | 6 | 1 |  |  | 7 |
| *T. aestivum* cv. Chinese Spring LC622404 | LSC |  |  | 22 | 5 | 3 | 2 | 32 |
|  | SSC |  |  | 1 |  |  |  | 1 |
|  | IR |  |  | 6 | 1 |  |  | 7 |
| *T. aestivum* cv. Chinese Spring MH051715 | LSC |  |  | 22 | 5 | 3 | 2 | 32 |
|  | SSC |  |  | 1 |  |  |  | 1 |
|  | IR |  |  | 6 | 1 |  |  | 7 |
| *T. aestivum* subsp. *carthlicoides* | LSC |  |  | 22 | 5 | 3 | 2 | 32 |
|  | SSC |  |  | 1 |  |  |  | 1 |
|  | IR |  |  | 6 | 1 |  |  | 7 |
| *T. aestivum* subsp*. tibeticum* | LSC |  |  | 22 | 5 | 3 | 2 | 32 |
|  | SSC |  |  | 1 |  |  |  | 1 |
|  | IR |  |  | 6 | 1 |  |  | 7 |
| *T. aestivum* var. *ferrugineum* | LSC |  |  | 22 | 5 | 3 | 2 | 32 |
|  | SSC |  |  | 1 |  |  |  | 1 |
|  | IR |  |  | 6 | 1 |  |  | 7 |
| *T. aestivum* var. *vavilovii* LC621349 | LSC |  |  | 22 | 5 | 3 | 2 | 32 |
|  | SSC |  |  | 1 |  |  |  | 1 |
|  | IR |  |  | 6 | 1 |  |  | 7 |
| *T. aestivum* var. *vavilovii* LC625865 | LSC |  |  | 22 | 6 | 3 | 3 | 34 |
|  | SSC |  |  | 1 |  |  |  | 1 |
|  | IR |  |  | 6 | 1 |  |  | 7 |
| *T. aestivum* AB042240 | LSC |  |  | 24 | 5 | 3 | 1 | 33 |
|  | SSC |  |  | 1 |  |  |  | 1 |
|  | IR |  |  | 6 | 1 |  |  | 7 |
| *T. aestivum* KJ592713 | LSC |  |  | 22 | 5 | 3 | 2 | 32 |
|  | SSC |  |  | 1 |  |  |  | 1 |
|  | IR |  |  | 7 | 1 |  |  | 8 |
| *T. carthlicum* var. *fuliginosum* | LSC |  |  | 22 | 5 | 3 | 2 | 32 |
|  | SSC |  |  | 1 |  |  |  | 1 |
|  | IR |  |  | 6 | 1 |  |  | 7 |
| *T. carthlicum* var. *rubiginosum* | LSC |  |  | 22 | 5 | 3 | 2 | 32 |
|  | SSC |  |  | 1 |  |  |  | 1 |
|  | IR |  |  | 6 | 1 |  |  | 7 |
| *T. compactum* | LSC |  |  | 22 | 5 | 3 | 2 | 32 |
|  | SSC |  |  | 1 |  |  |  | 1 |
|  | IR |  |  | 6 | 1 |  |  | 7 |
| *T. macha* var. *colchicum* | LSC |  |  | 22 | 5 | 3 | 2 | 32 |
|  | SSC |  |  | 1 |  |  |  | 1 |
|  | IR |  |  | 6 | 1 |  |  | 7 |
| *T. macha* var. *megrelicum* | LSC |  |  | 22 | 5 | 3 | 2 | 32 |
|  | SSC |  |  | 1 |  |  |  | 1 |
|  | IR |  |  | 6 | 1 |  |  | 7 |
| *T. macha* var*. palaeoimereticum* | LSC |  |  | 22 | 5 | 3 | 2 | 32 |
|  | SSC |  |  | 1 |  |  |  | 1 |
|  | IR |  |  | 6 | 1 |  |  | 7 |
| *T. macha* NC_025955 | LSC |  |  | 22 | 5 | 3 | 2 | 32 |
|  | SSC |  |  | 1 |  |  |  | 1 |
|  | IR |  |  | 6 | 1 |  |  | 7 |
| *T. spelta* | LSC |  |  | 22 | 6 | 3 | 3 | 34 |
|  | SSC |  |  | 1 |  |  |  | 1 |
|  | IR |  |  | 6 | 1 |  |  | 7 |
| *T. sphaerococcum* | LSC |  |  | 22 | 5 | 3 | 2 | 32 |
|  | SSC |  |  | 1 |  |  |  | 1 |
|  | IR |  |  | 6 | 1 |  |  | 7 |
| *T. timopheevii* cv. TA0941 | LSC |  |  | 22 | 5 | 4 | 2 | 33 |
|  | SSC |  |  | 1 |  |  |  | 1 |
|  | IR |  |  | 6 | 1 |  |  | 7 |
| *T. timopheevii* cv. TA944 | LSC |  |  | 22 | 5 | 4 | 2 | 33 |
|  | SSC |  |  | 1 |  |  |  | 1 |
|  | IR |  |  | 6 | 1 |  |  | 7 |
| *T. timopheevii* cv. TA1485 | LSC |  |  | 22 | 5 | 4 | 2 | 33 |
|  | SSC |  |  | 1 |  |  |  | 1 |
|  | IR |  |  | 6 | 1 |  |  | 7 |
| *T. timopheevii* cv. Tim01 | LSC |  |  | 22 | 5 | 4 | 2 | 33 |
|  | SSC |  |  | 1 |  |  |  | 1 |
|  | IR |  |  | 6 | 1 |  |  | 7 |
| *T. turgidum* cv. PI520121 | LSC |  |  | 22 | 5 | 3 | 2 | 32 |
|  | SSC |  |  | 1 |  |  |  | 1 |
|  | IR |  |  | 7 | 1 |  |  | 8 |
| *T. turgidum* cv. TA0060 | LSC |  |  | 22 | 5 | 3 | 2 | 32 |
|  | SSC |  |  | 1 |  |  |  | 1 |
|  | IR |  |  | 7 | 1 |  |  | 8 |
| *T. turgidum* cv. TA0073 | LSC |  |  | 22 | 5 | 3 | 2 | 32 |
|  | SSC |  |  | 1 |  |  |  | 1 |
|  | IR |  |  | 7 | 1 |  |  | 8 |
| *T. turgidum* cv. TA1133 | LSC |  |  | 22 | 6 | 3 | 3 | 34 |
|  | SSC |  |  | 1 |  |  |  | 1 |
|  | IR |  |  | 7 | 1 |  |  | 8 |
| *T. turgidum* cv. TA2801 | LSC |  |  | 22 | 5 | 3 | 2 | 32 |
|  | SSC |  |  | 1 |  |  |  | 1 |
|  | IR |  |  | 7 | 1 |  |  | 8 |
| *T. turgidum* subsp. *durum cv.* Langdon | LSC |  |  | 22 | 5 | 3 | 2 | 32 |
|  | SSC |  |  | 1 |  |  |  | 1 |
|  | IR |  |  | 5 | 1 |  |  | 6 |
| *T. turgidum* subsp*. durum* | LSC |  |  | 22 | 5 | 3 | 2 | 32 |
|  | SSC |  |  | 1 |  |  |  | 1 |
|  | IR |  |  | 6 | 1 |  |  | 7 |
| *T. turgidum* subsp. *paleocolchicum* | LSC |  |  | 22 | 5 | 3 | 2 | 32 |
|  | SSC |  |  | 1 |  |  |  | 1 |
|  | IR |  |  | 6 | 1 |  |  | 7 |
| *T. urartu* KJ174105 | LSC |  |  | 22 | 5 | 3 | 2 | 32 |
|  | SSC |  |  | 1 |  |  |  | 1 |
|  | IR |  |  | 6 | 1 |  |  | 7 |
| *T. zhukovskyi* | LSC |  |  | 22 | 5 | 4 | 2 | 33 |
|  | SSC |  |  | 1 |  |  |  | 1 |
|  | IR |  |  | 6 | 1 |  |  | 7 |
| Total |  |  |  | 1197 (71.98%) | 249 (14.97%) | 133 (8.00%) | 84 (5.05%) |  |

**Figure S1**. Bayesian Inference (BI) and Maximum Likelihood (ML) phylogenetic tree of 17 specific barcode regions markers.


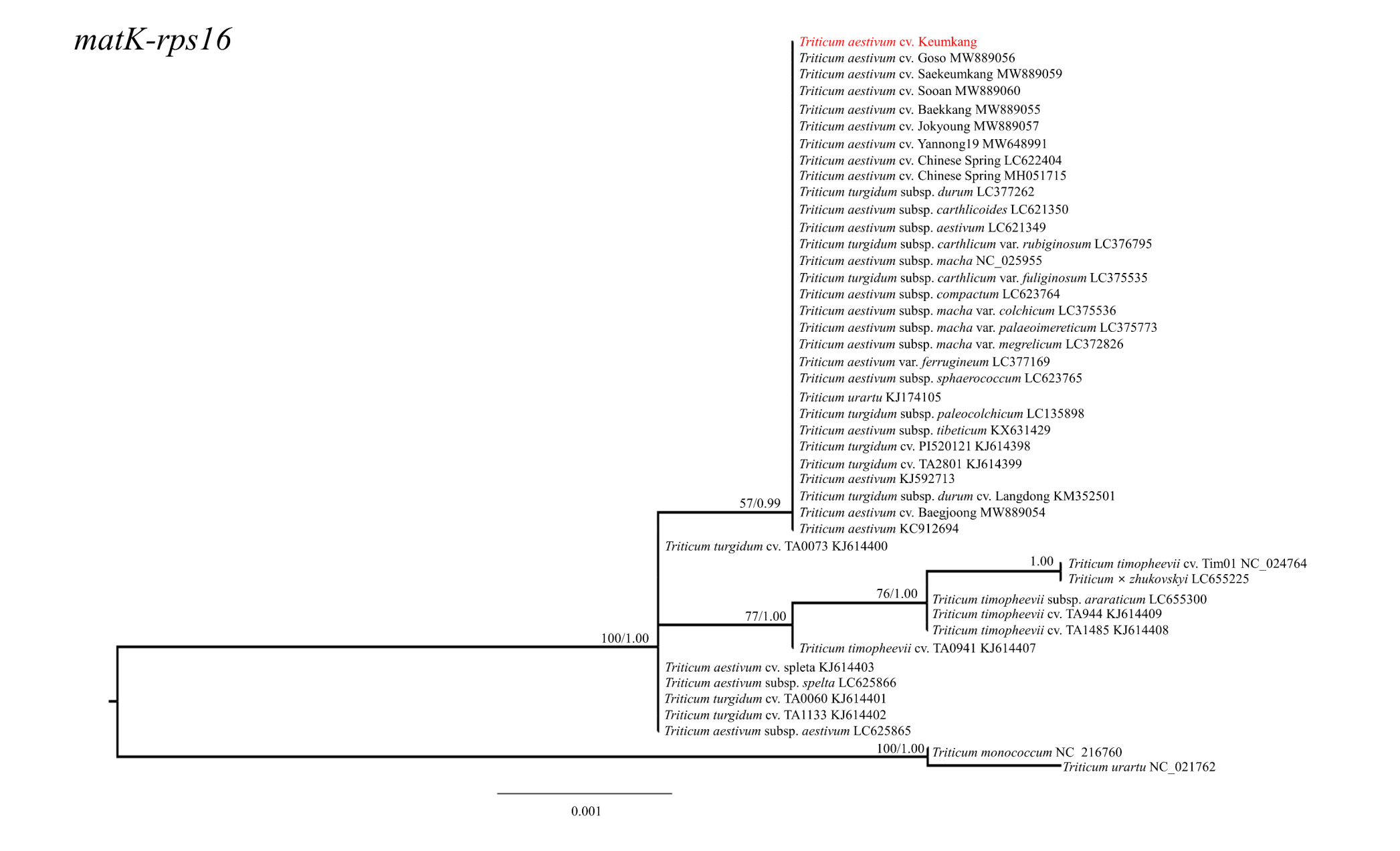


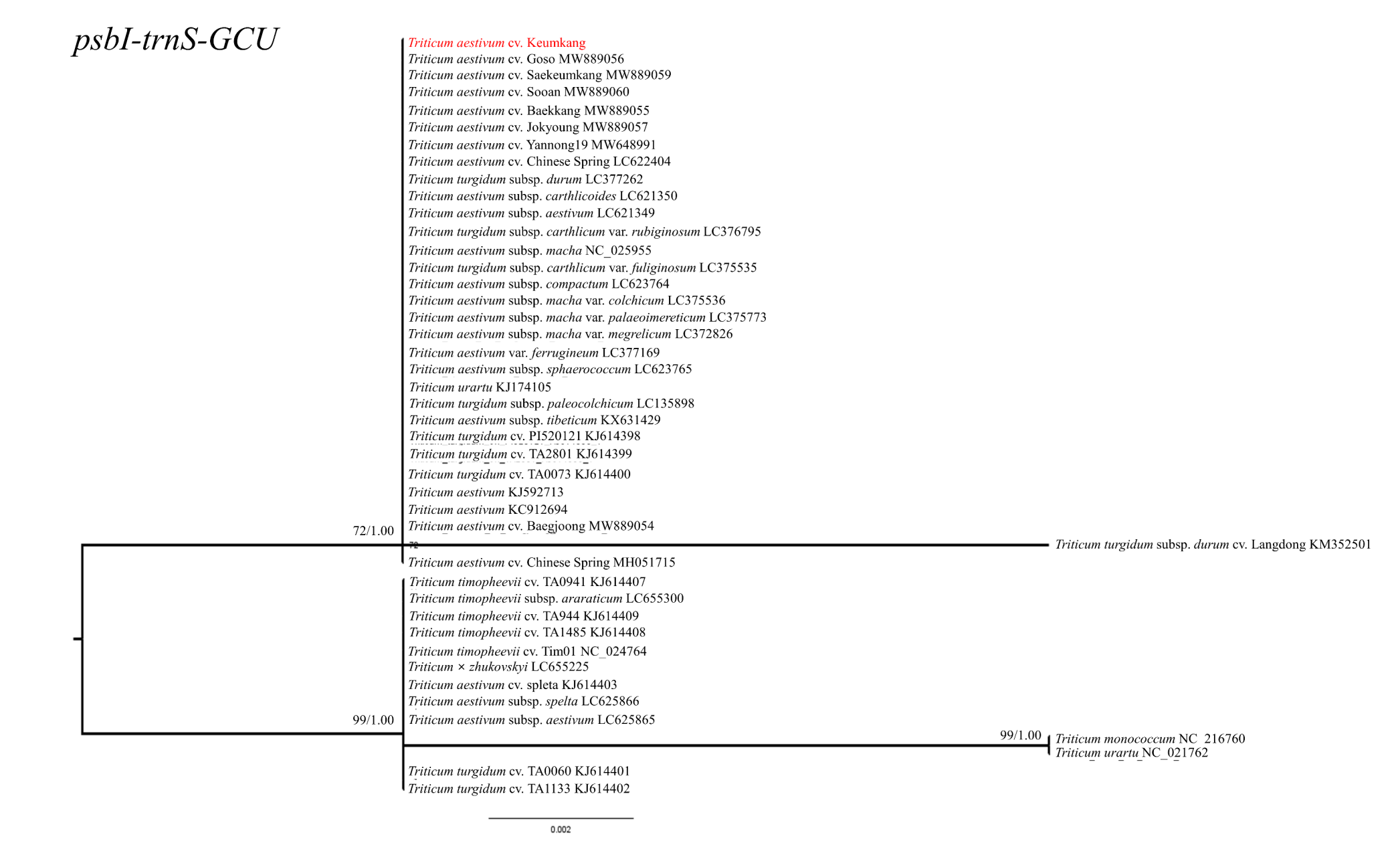


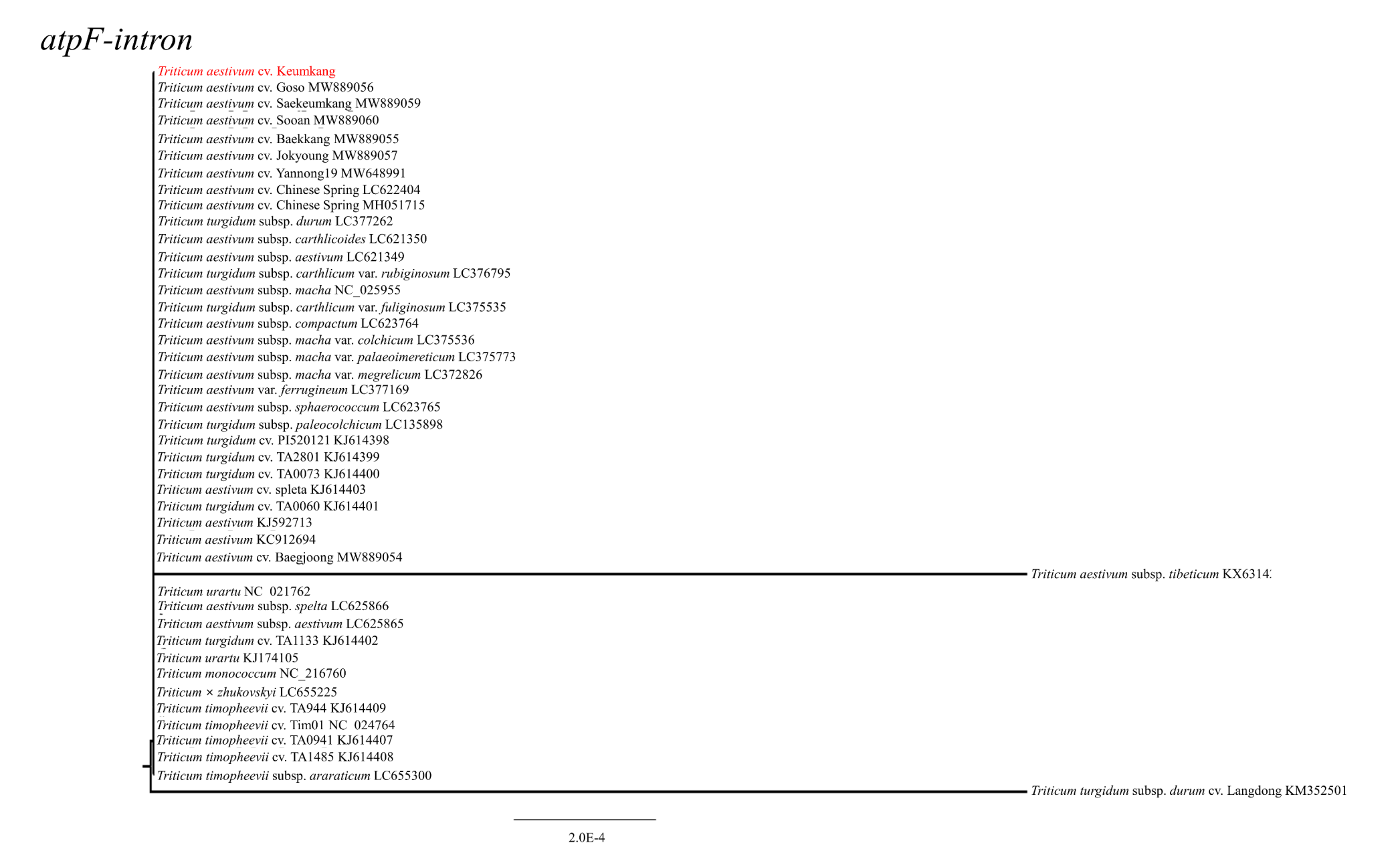


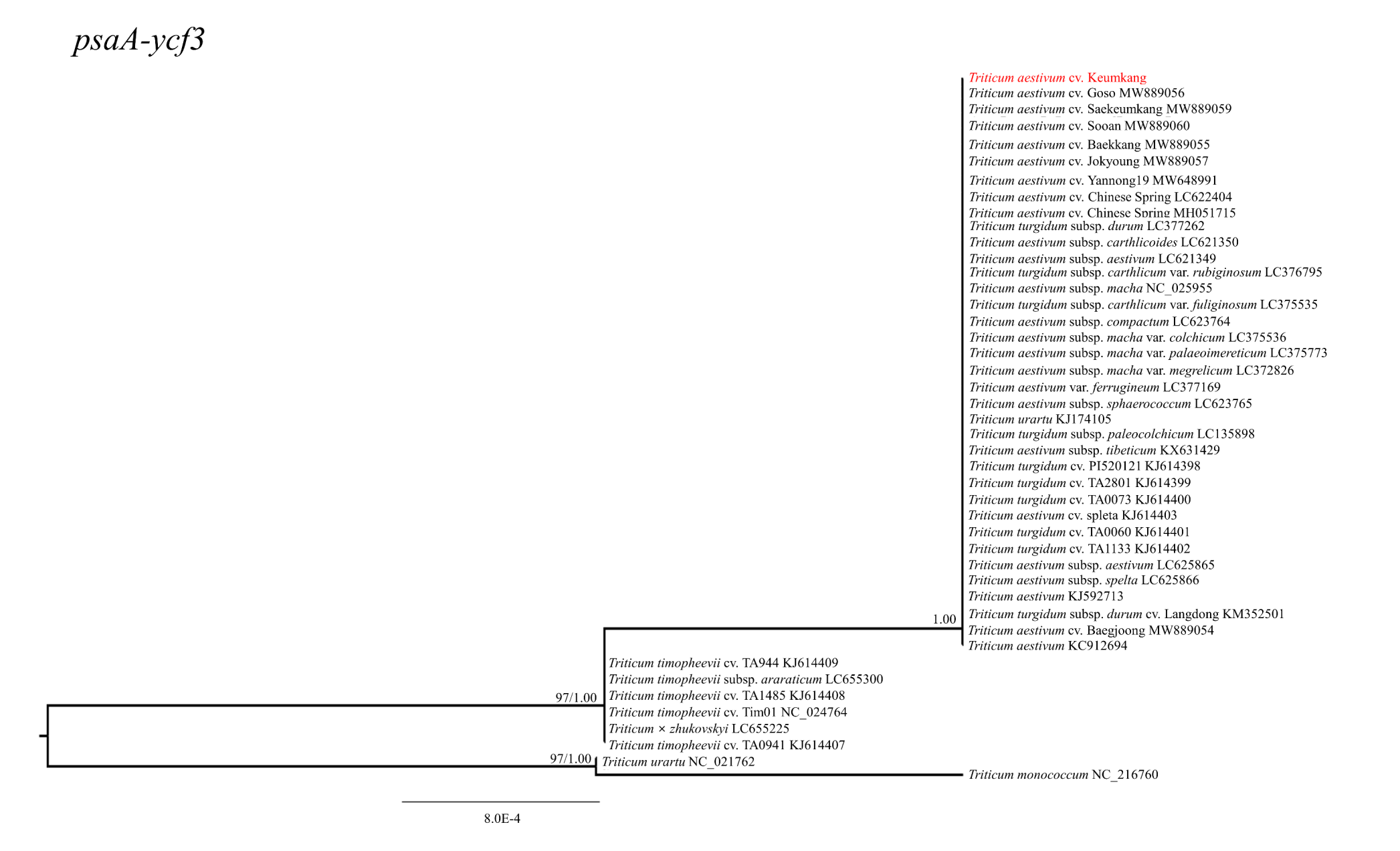


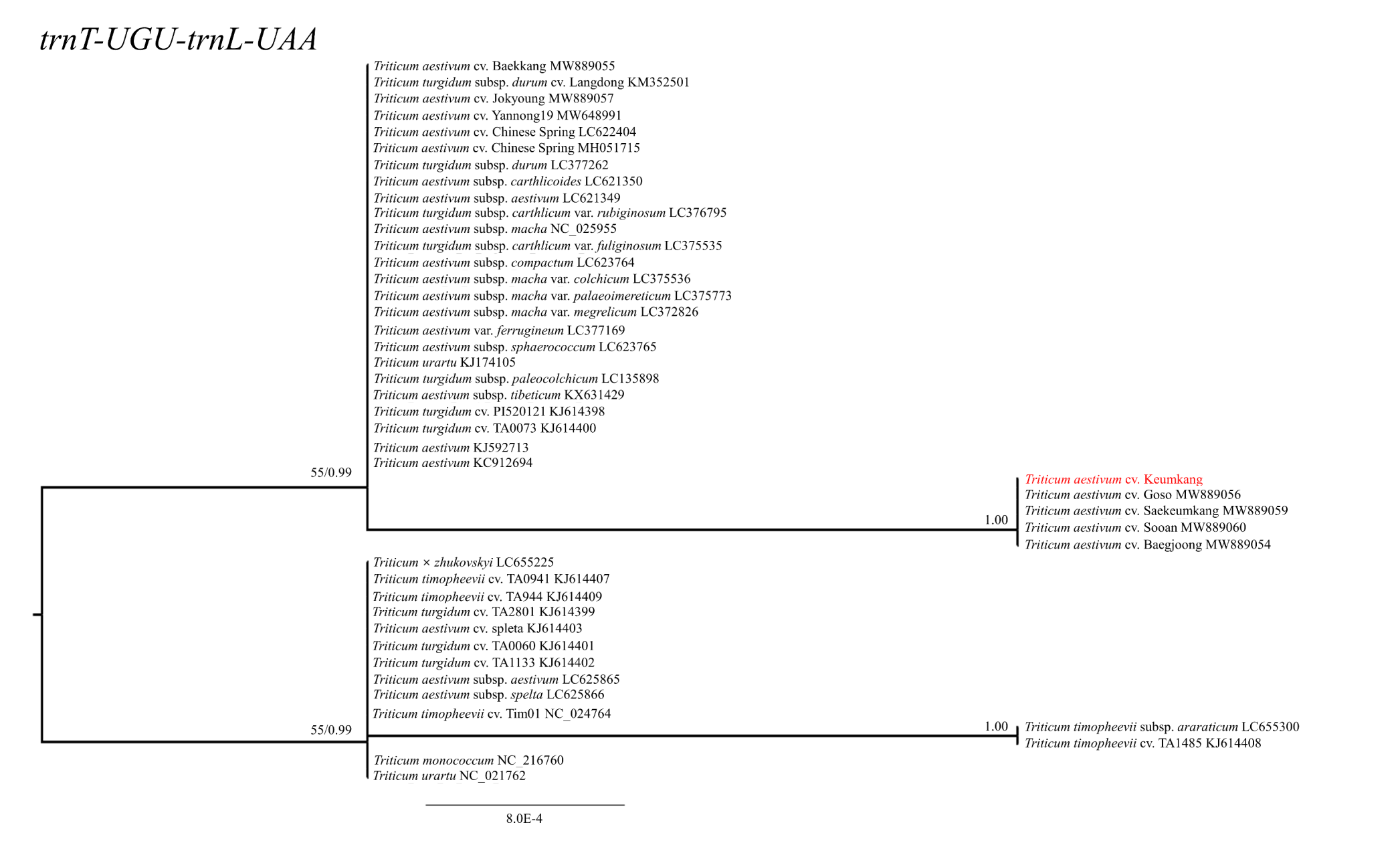


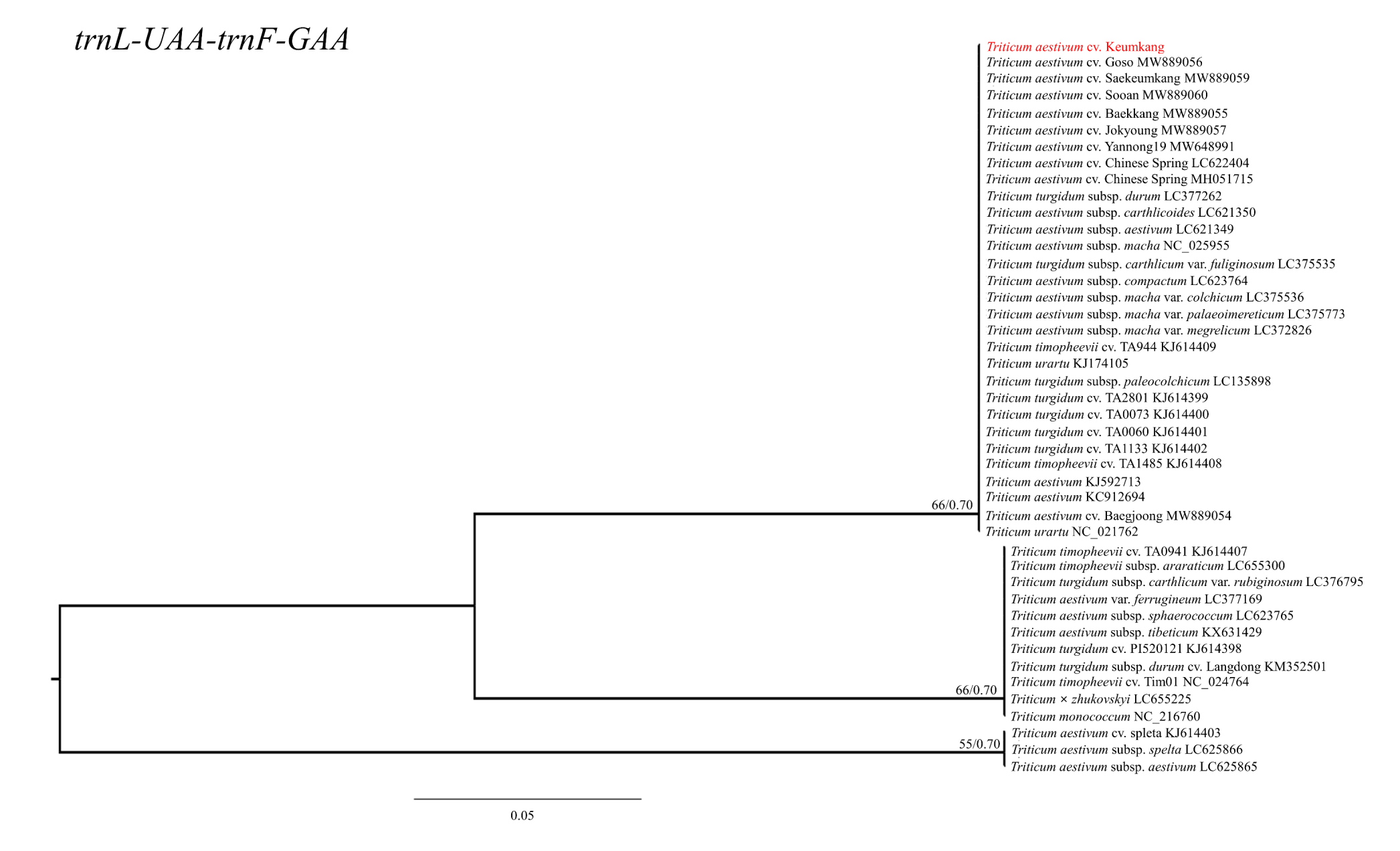


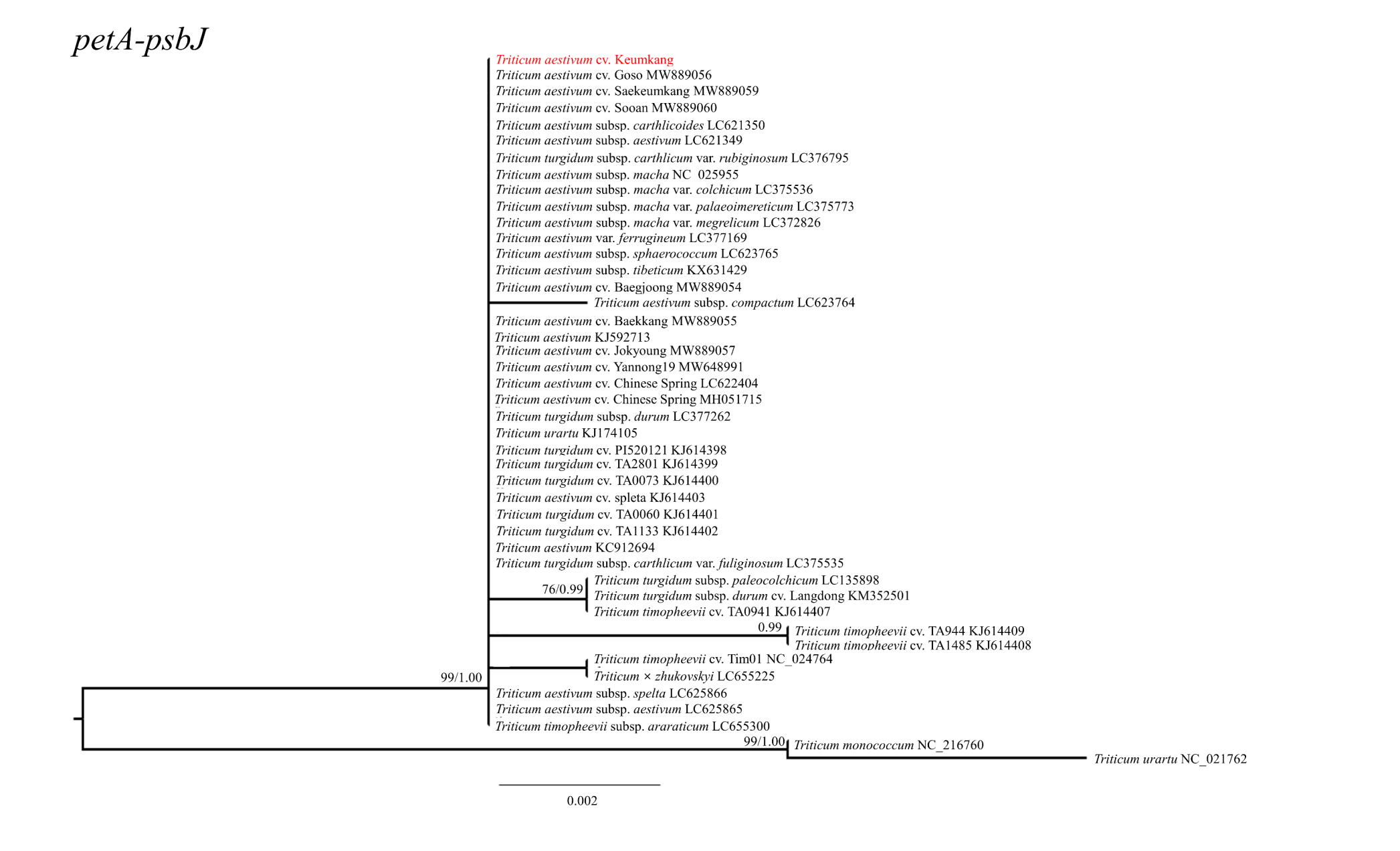


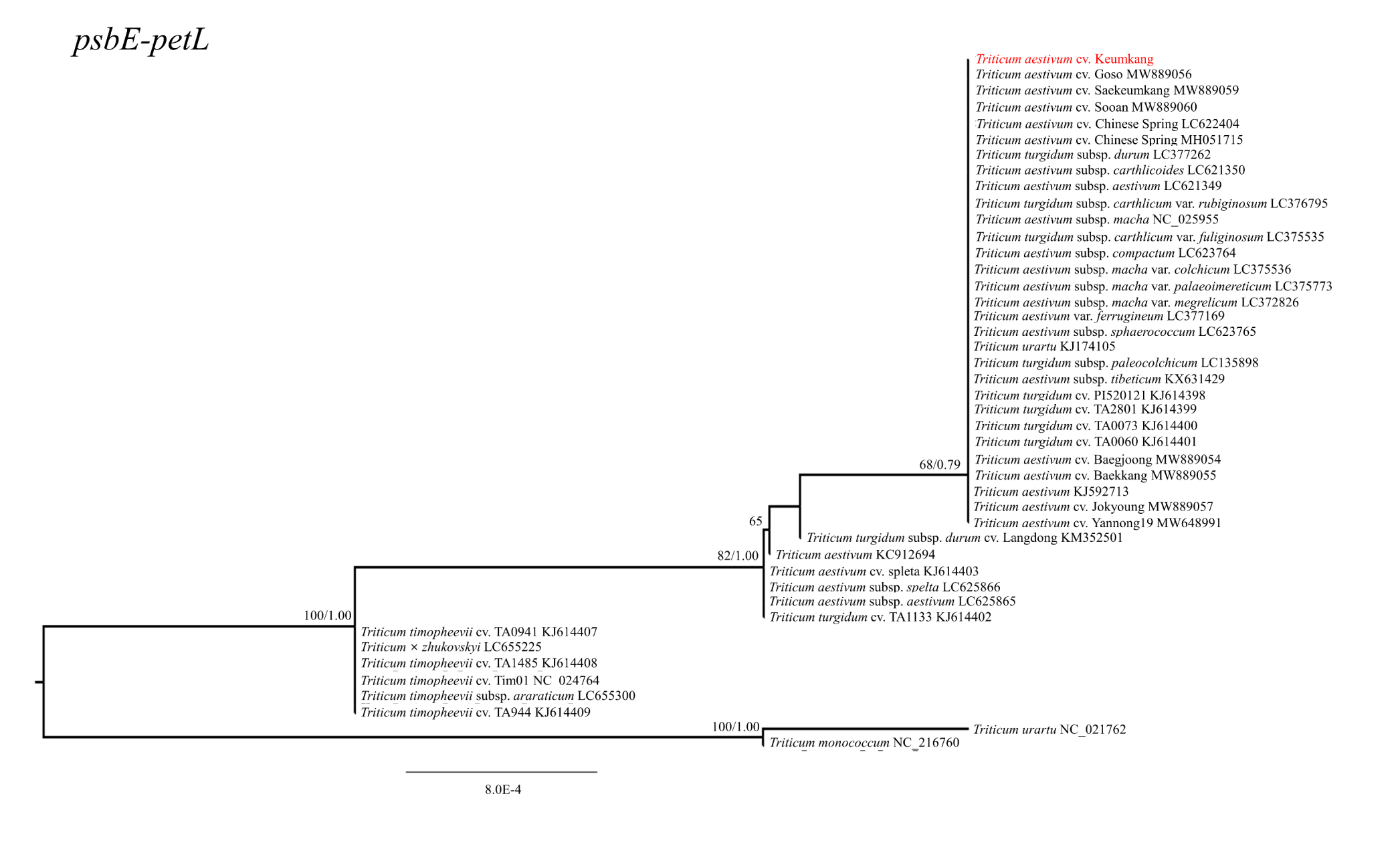


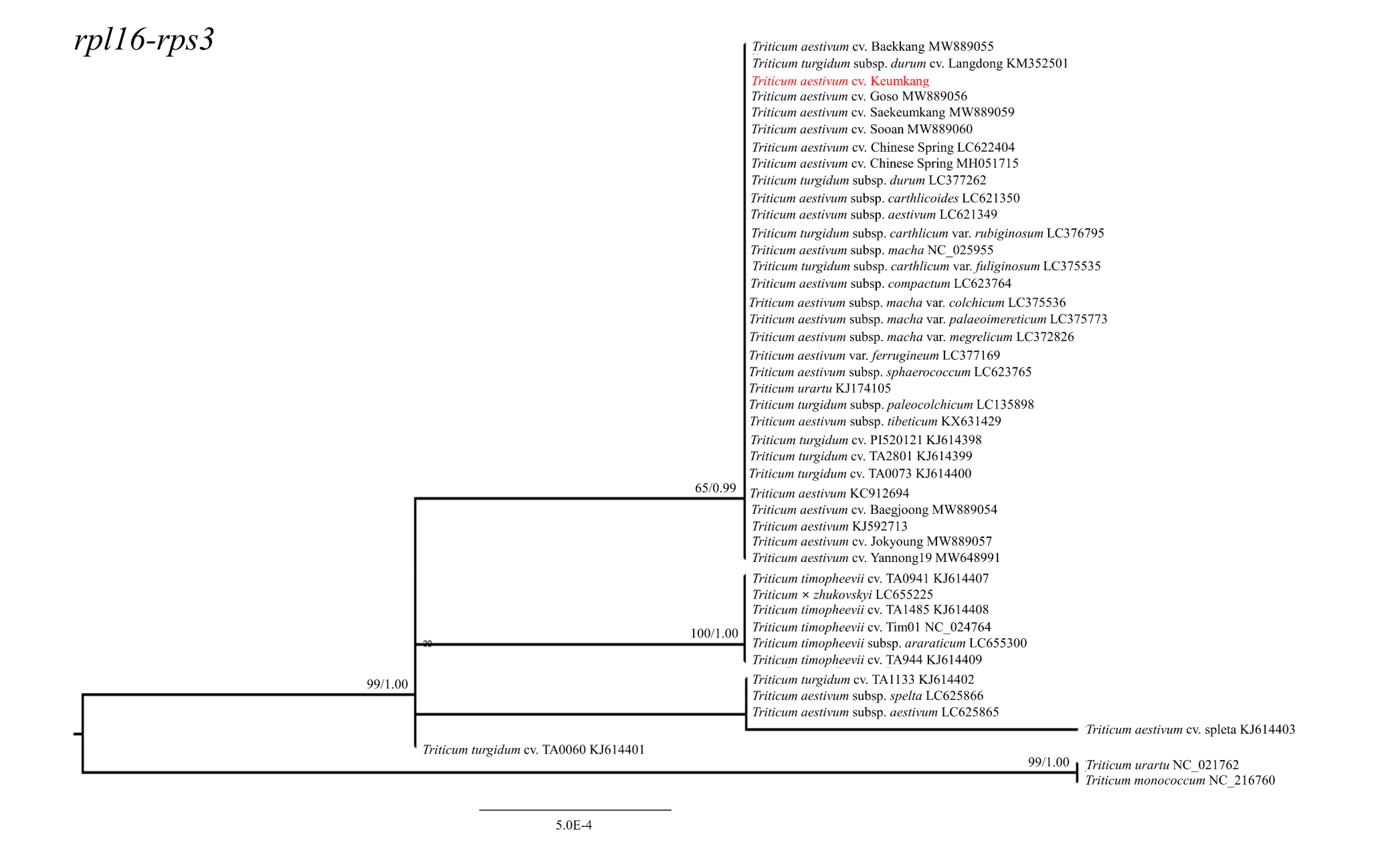


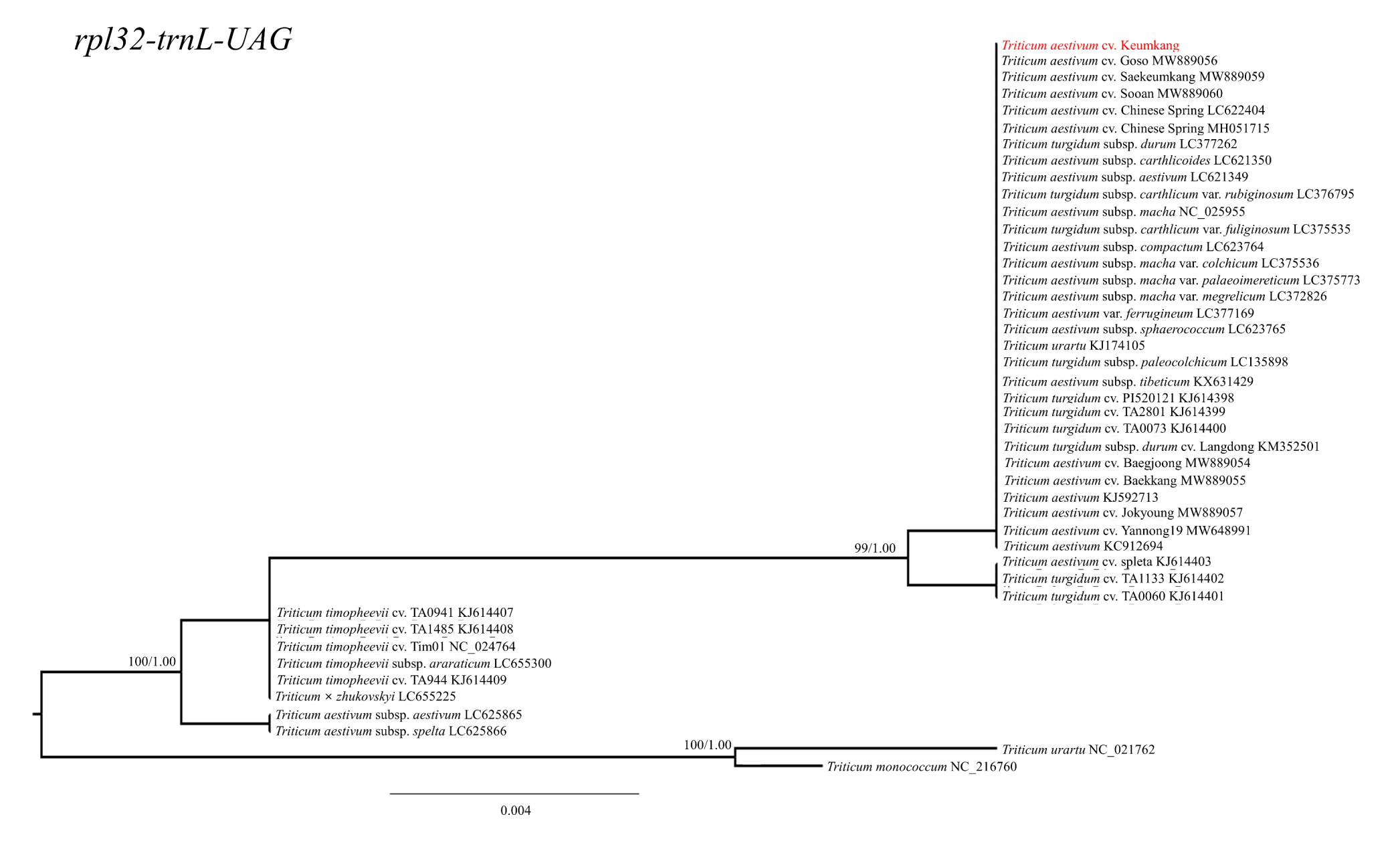


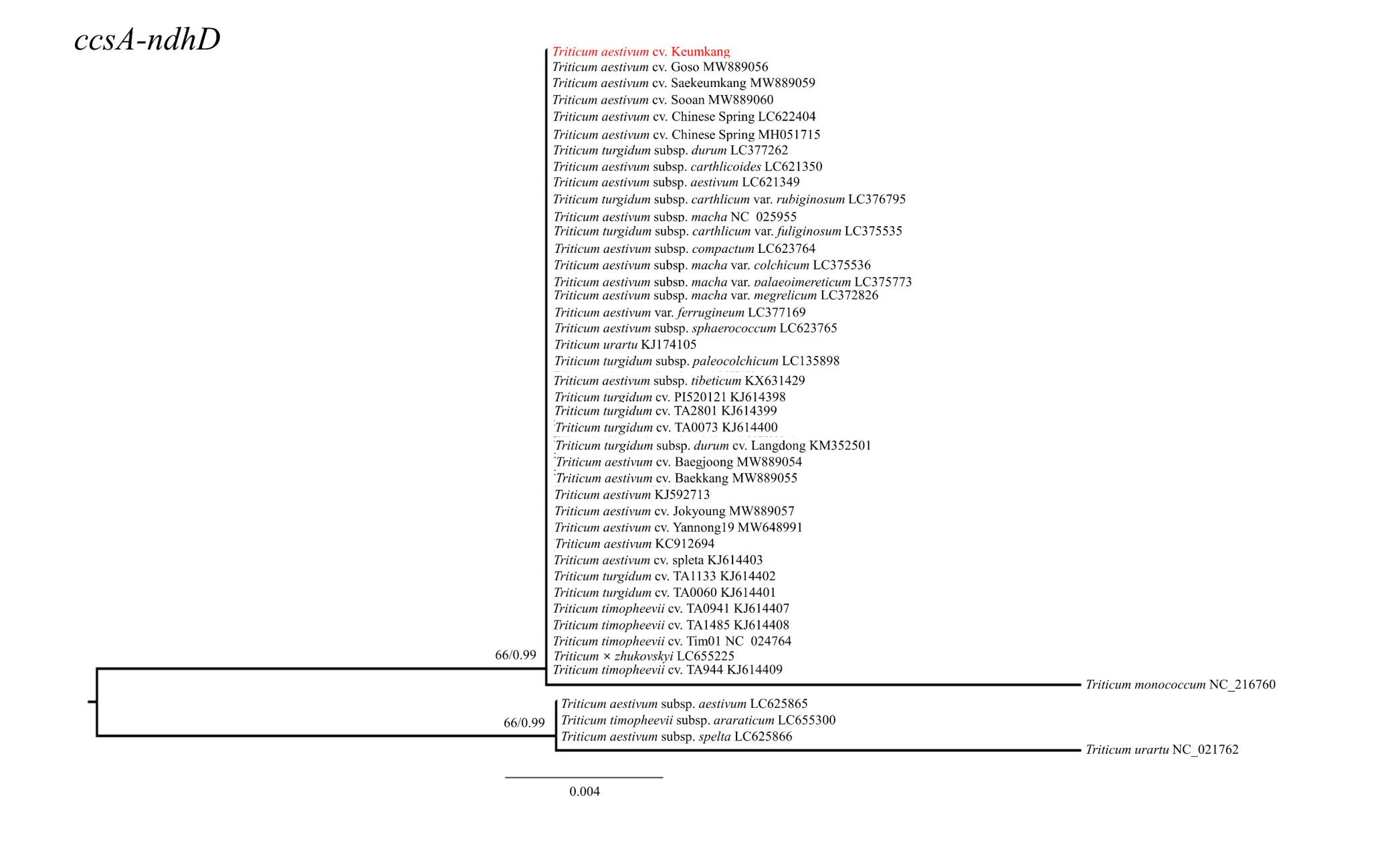


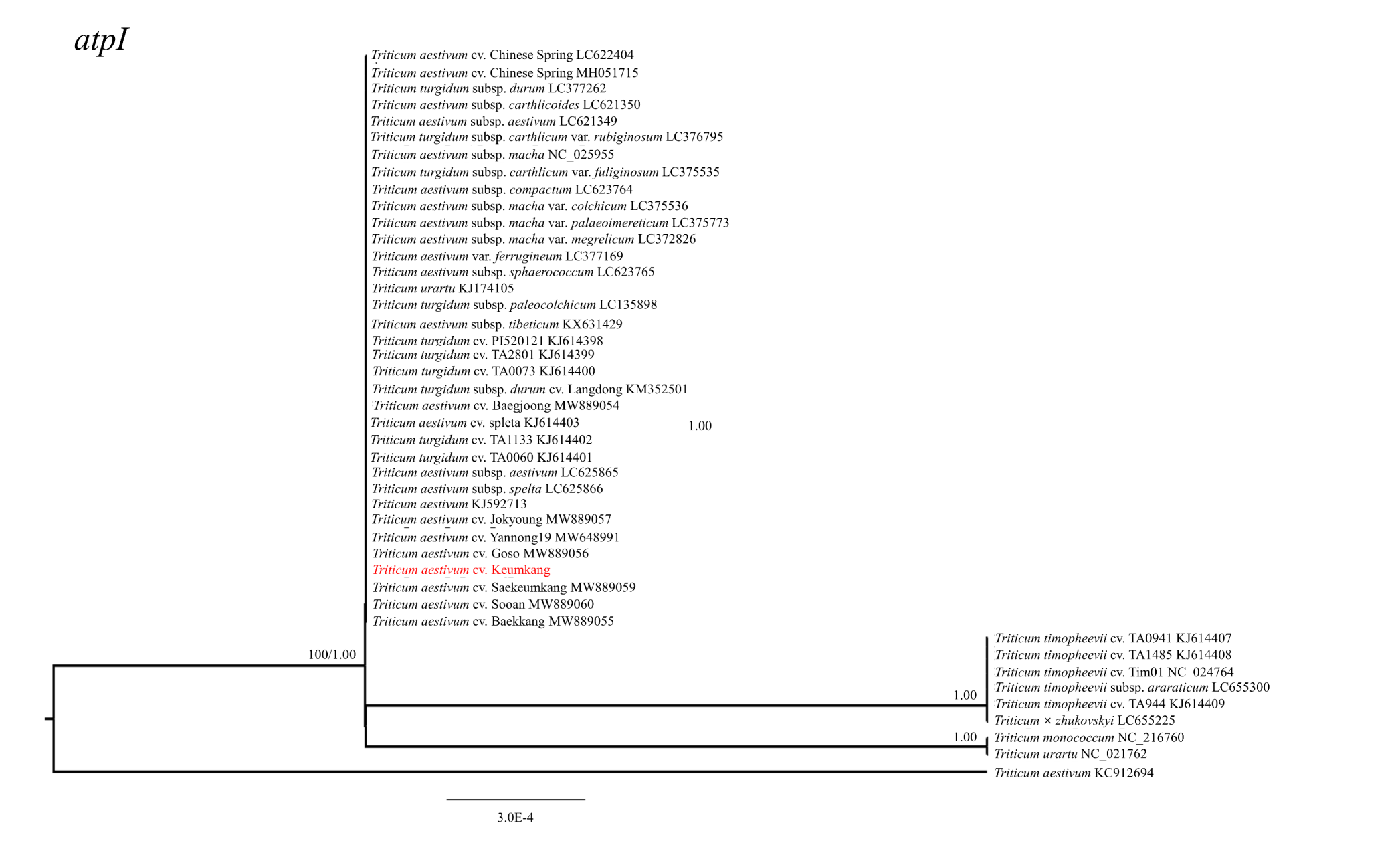


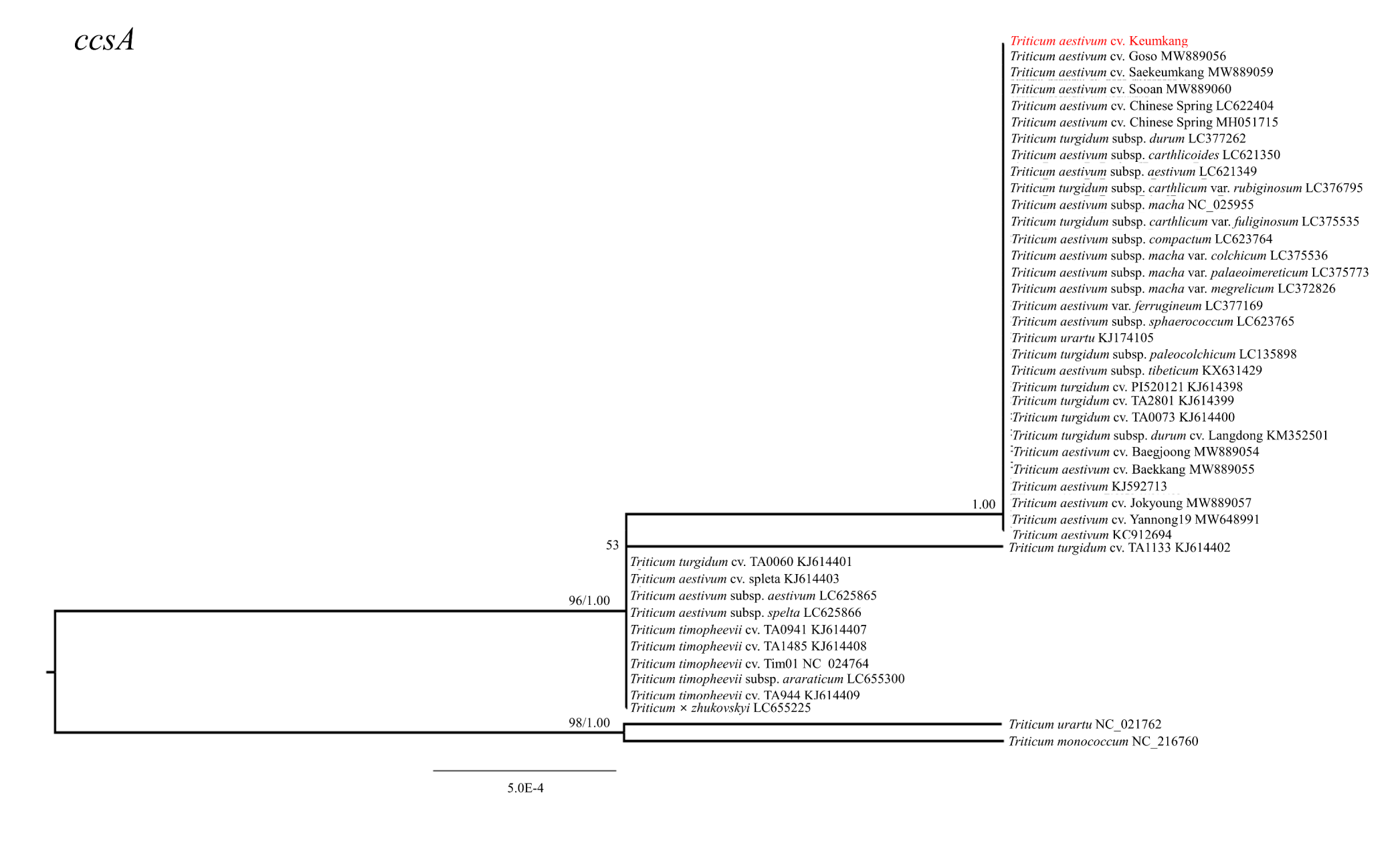


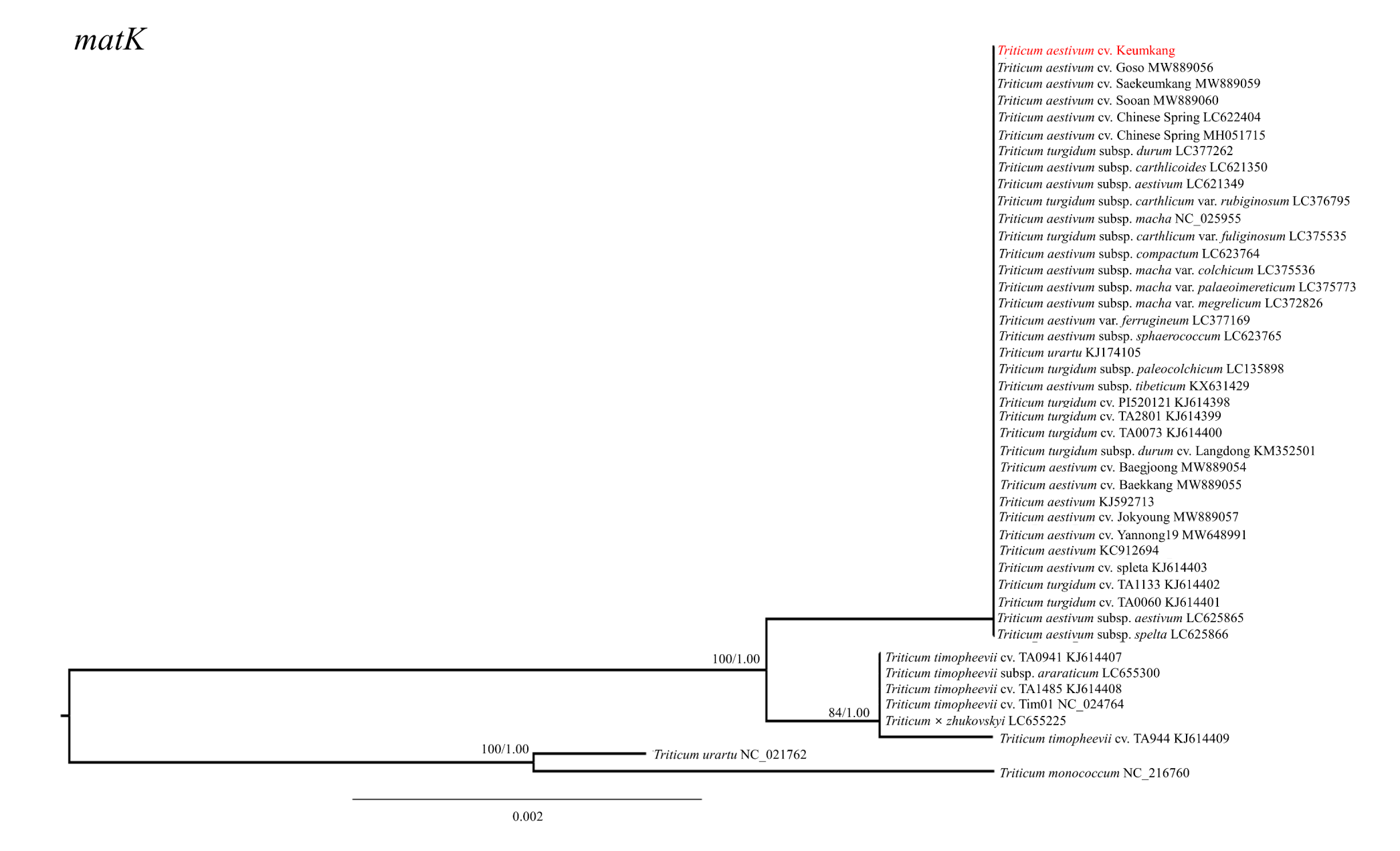


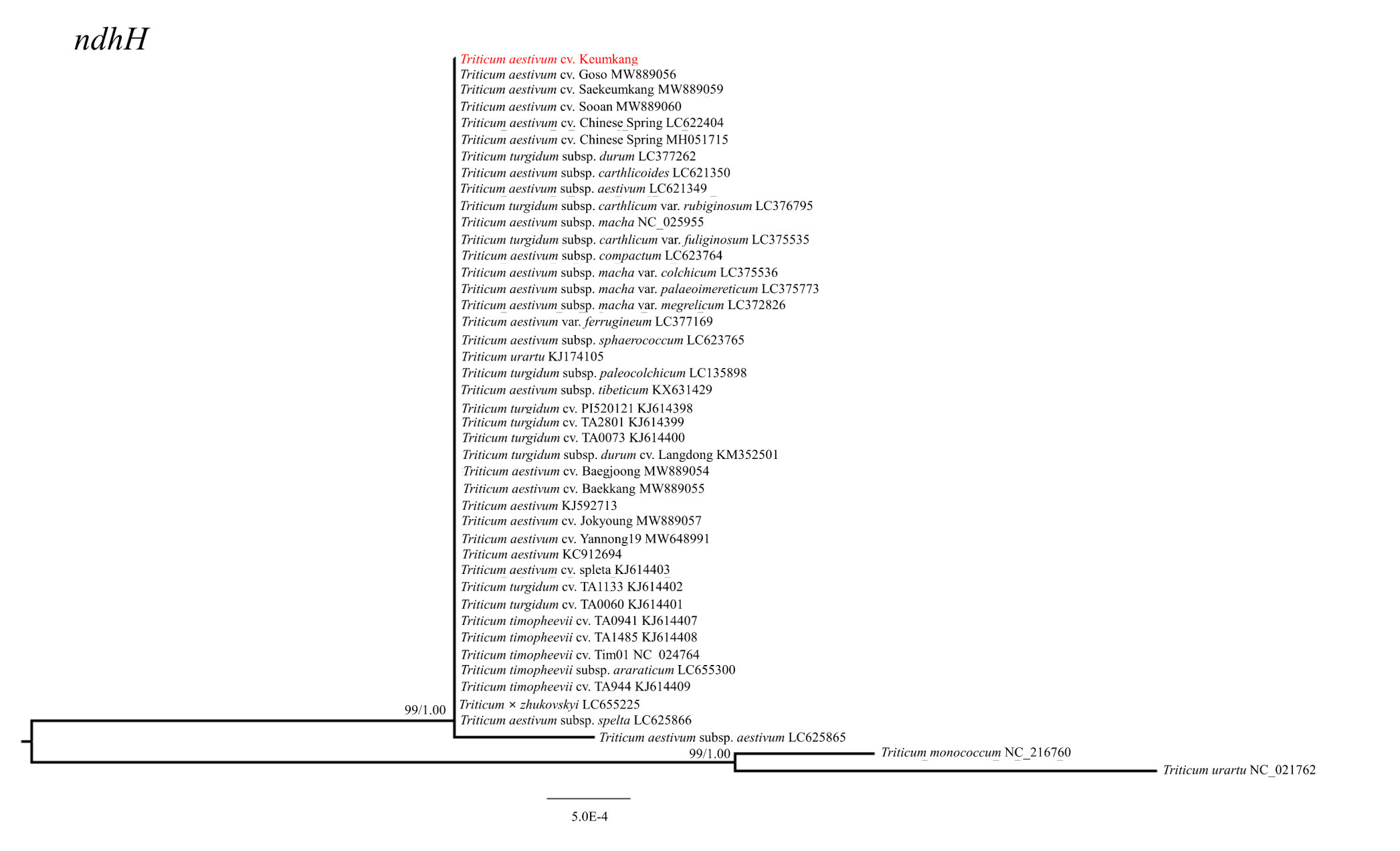


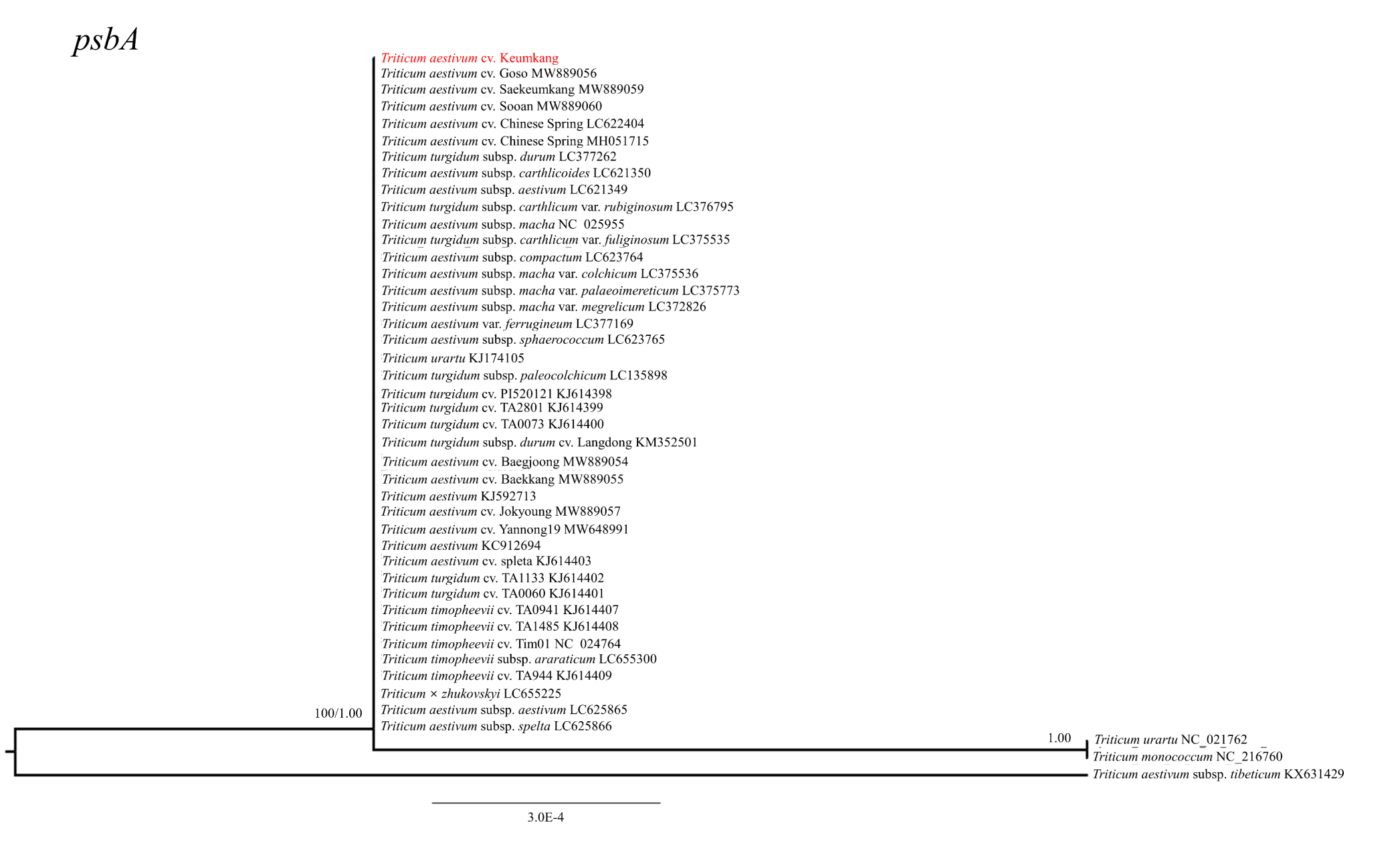


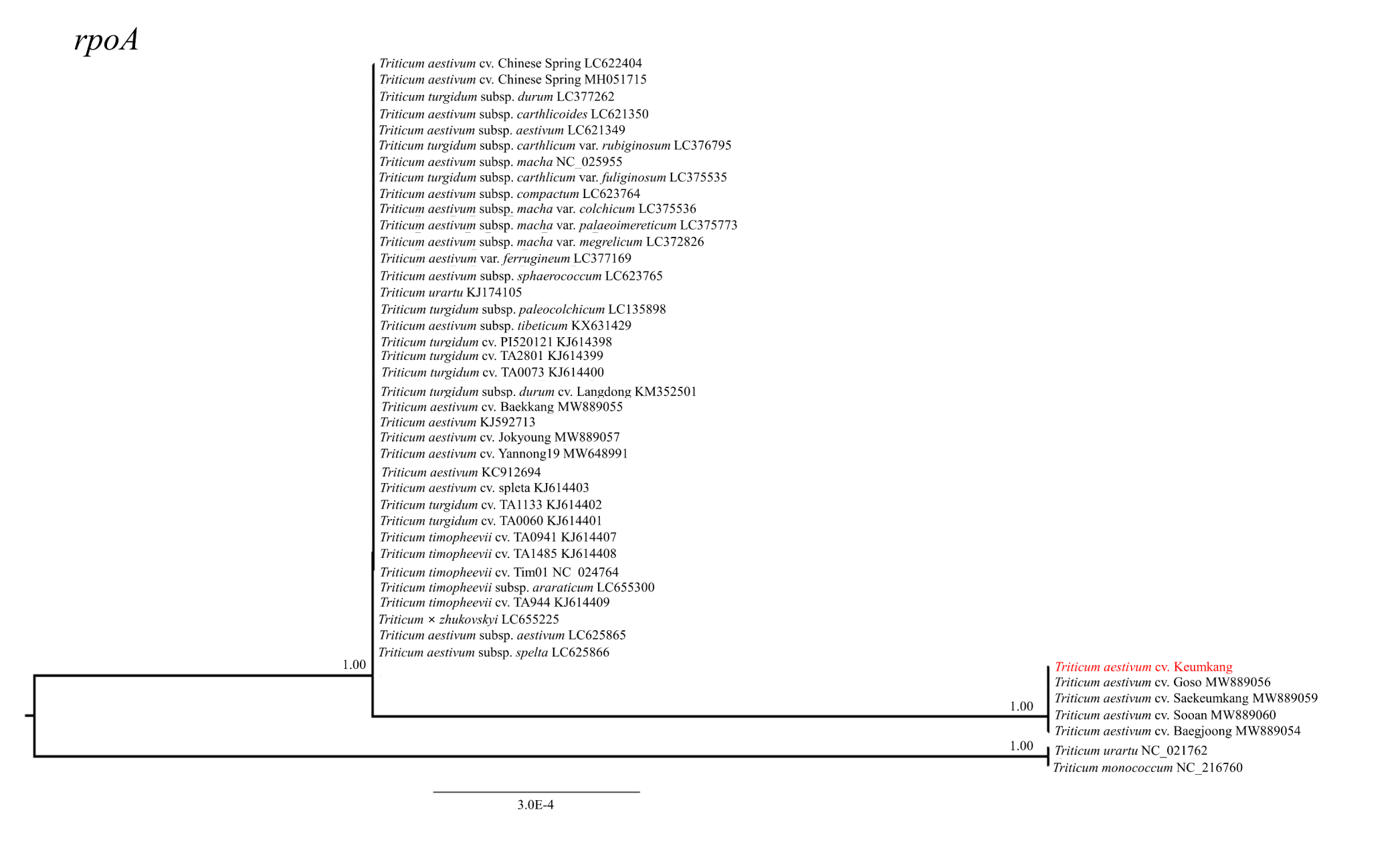


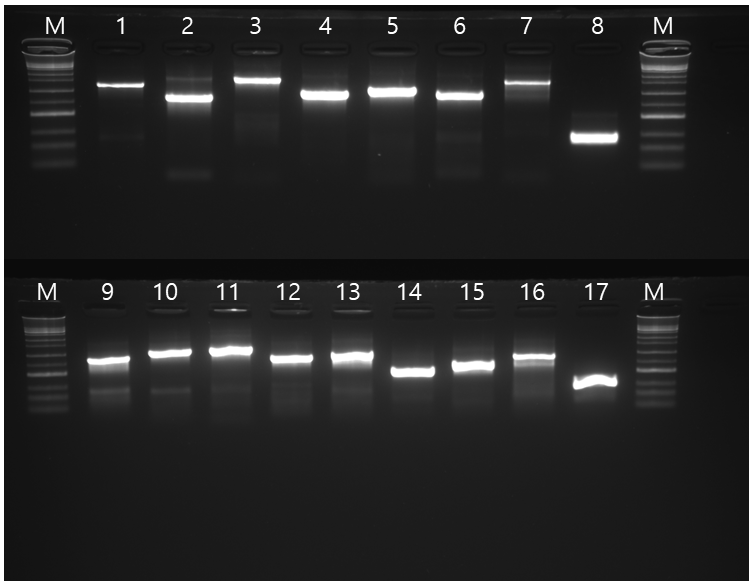


**Figure S2**. PCR amplification products of specific barcoding markers 1 to 17. M: DNA ladder, 1: *ccsA*, 2: *atpI*, 3: *matK*, 4: *ndhH*, 5: *psbA*, 6: *rpoA*, 7: *matK-rps16*, 8: *psbI-trnS-GCU*, 9: *atpF-intron*, 10: *psaA-ycf3*, 11: *trnT-UGU-trnL-UAA*, 12: *trnL-UAA-trnF-GAA*, 13: *petA-psbJ*, 14: *psbE-petL*, 15: *rpl16-rps3*, 16: *rpl32-trnL-UAG*, 17: *ccsA-ndhD*.


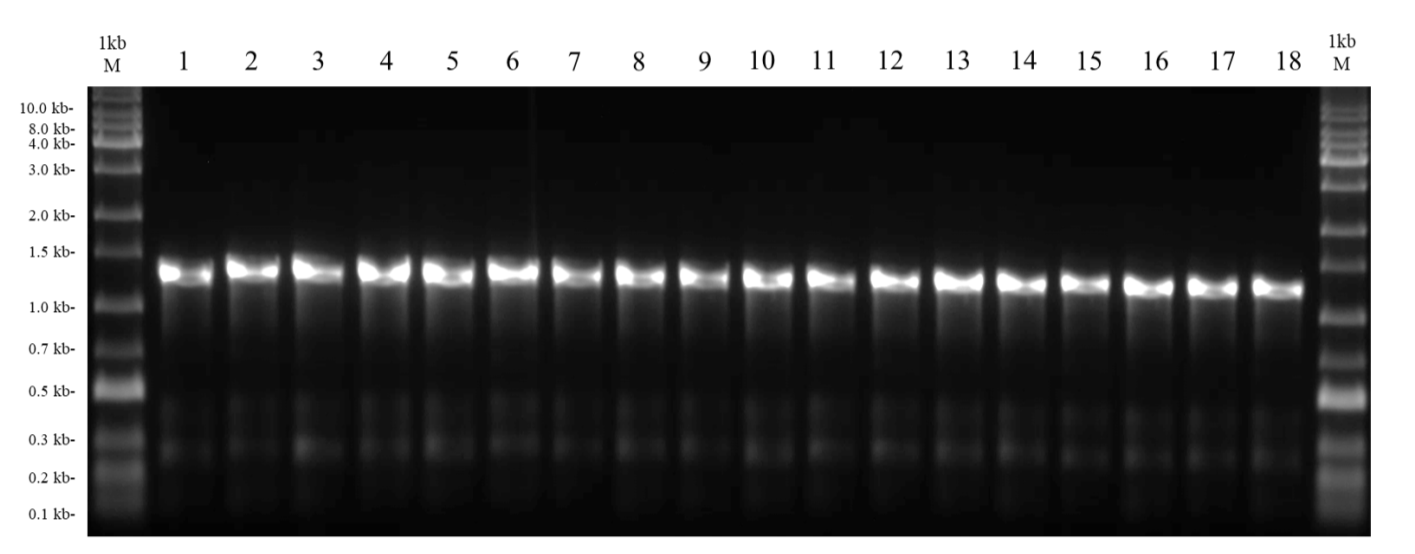


**Figure S3**. PCR amplification products of cultivar 1-18 for the specific barcoding marker *ccsA* gene. M: DNA ladder. (1: *Triticum aestivum* subsp. *aestivum*, IT No.:172226, UNK; 2: *Triticum urartu*, IT No.:177184, TUR; 3: *Triticum urartu*, IT No.:177185, ARM; 4: *Triticum aestivum* subsp. *aestivum*, IT No.:185166, UNK; 5: *Triticum urartu*, IT No.:202089, UNK; 6: *Aegilops biuncialis*, IT No.:204618, TUR; 7: *Triticum aestivum* subsp. *aestivum*, IT No.:267172, USA; 8: *Triticum turgidum* subsp. *durum*, IT No.:269514, MNG; 9: *Triticum turgidum* subsp. *durum*, IT No.:269515, MNG; 10: *Triticum aestivum* subsp. *aestivum*, IT No.:293620, CHN; 11: *Aegilops uniaristata*, IT No.:302223, GRC; 12: *Aegilops ventricosa*, IT No.:302224, FRA; 13: *Triticum aestivum* subsp. *aestivum*, IT No.:324662, TUR; 14: *Triticum turgidum* subsp. *durum*, IT No.:330427, CAN; 15: *Triticum urartu*, IT No.:330603, SYR; 16: *Triticum turgidum* subsp. *durum*, IT No.:340493, CAN; 17: *Triticum aestivum* subsp. *aestivum*, IT No.:341831, KOR; 18: *Aegilops cylindrica*, IT No.:352075, TJK).


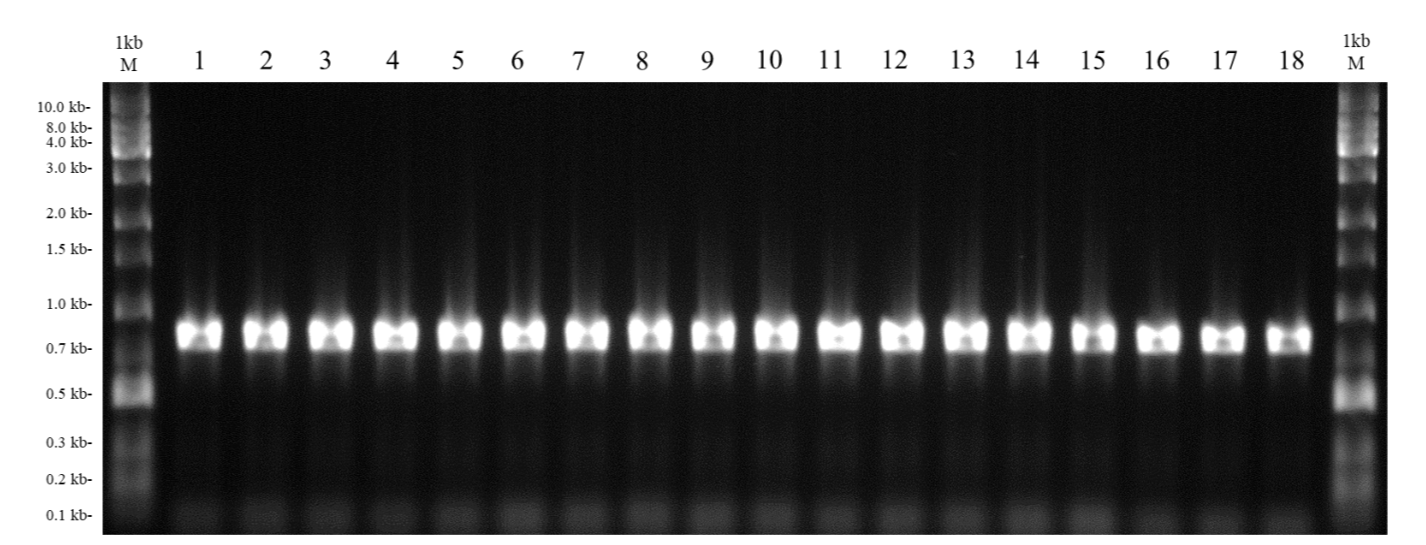


**Figure S4**. PCR amplification products of cultivar 1-18 for the specific barcoding marker *atpI* gene. M: DNA ladder. (1: *Triticum aestivum* subsp. *aestivum*, IT No.:172226, UNK; 2: *Triticum urartu*, IT No.:177184, TUR; 3: *Triticum urartu*, IT No.:177185, ARM; 4: *Triticum aestivum* subsp. *aestivum*, IT No.:185166, UNK; 5: *Triticum urartu*, IT No.:202089, UNK; 6: *Aegilops biuncialis*, IT No.:204618, TUR; 7: *Triticum aestivum* subsp. *aestivum*, IT No.:267172, USA; 8: *Triticum turgidum* subsp. *durum*, IT No.:269514, MNG; 9: *Triticum turgidum* subsp. *durum*, IT No.:269515, MNG; 10: *Triticum aestivum* subsp. *aestivum*, IT No.:293620, CHN; 11: *Aegilops uniaristata*, IT No.:302223, GRC; 12: *Aegilops ventricosa*, IT No.:302224, FRA; 13: *Triticum aestivum* subsp. *aestivum*, IT No.:324662, TUR; 14: *Triticum turgidum* subsp. *durum*, IT No.:330427, CAN; 15: *Triticum urartu*, IT No.:330603, SYR; 16: *Triticum turgidum* subsp. *durum*, IT No.:340493, CAN; 17: *Triticum aestivum* subsp. *aestivum*, IT No.:341831, KOR; 18: *Aegilops cylindrica*, IT No.:352075, TJK).


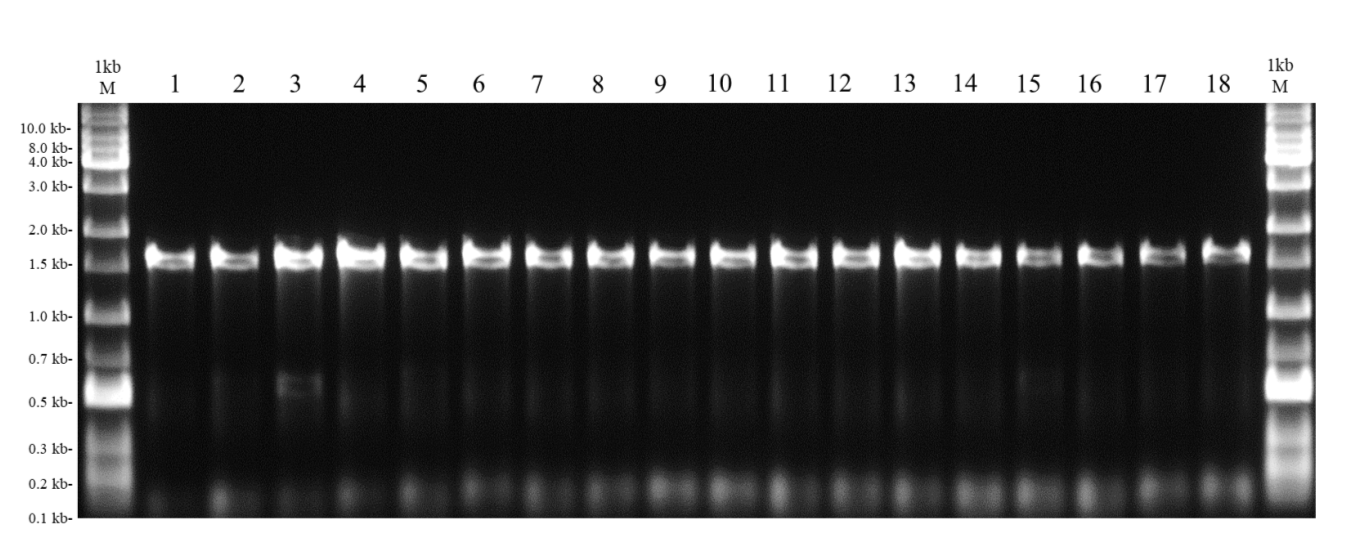


**Figure S5**. PCR amplification products of cultivar 1-18 for the specific barcoding marker *matK* gene. M: DNA ladder. (1: *Triticum aestivum* subsp. *aestivum*, IT No.:172226, UNK; 2: *Triticum urartu*, IT No.:177184, TUR; 3: *Triticum urartu*, IT No.:177185, ARM; 4: *Triticum aestivum* subsp. *aestivum*, IT No.:185166, UNK; 5: *Triticum urartu*, IT No.:202089, UNK; 6: *Aegilops biuncialis*, IT No.:204618, TUR; 7: *Triticum aestivum* subsp. *aestivum*, IT No.:267172, USA; 8: *Triticum turgidum* subsp. *durum*, IT No.:269514, MNG; 9: *Triticum turgidum* subsp. *durum*, IT No.:269515, MNG; 10: *Triticum aestivum* subsp. *aestivum*, IT No.:293620, CHN; 11: *Aegilops uniaristata*, IT No.:302223, GRC; 12: *Aegilops ventricosa*, IT No.:302224, FRA; 13: *Triticum aestivum* subsp. *aestivum*, IT No.:324662, TUR; 14: *Triticum turgidum* subsp. *durum*, IT No.:330427, CAN; 15: *Triticum urartu*, IT No.:330603, SYR; 16: *Triticum turgidum* subsp. *durum*, IT No.:340493, CAN; 17: *Triticum aestivum* subsp. *aestivum*, IT No.:341831, KOR; 18: *Aegilops cylindrica*, IT No.:352075, TJK).


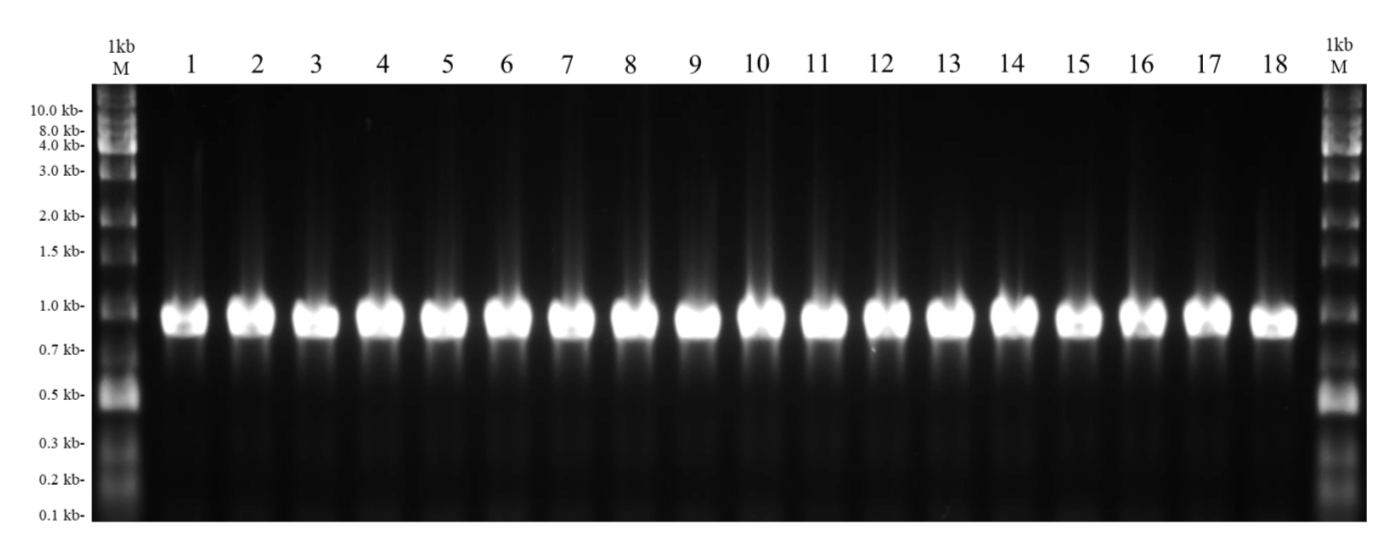


**Figure S6**. PCR amplification products of cultivar 1-18 for the specific barcoding marker *ndhH* gene. M: DNA ladder. (1: *Triticum aestivum* subsp. *aestivum*, IT No.:172226, UNK; 2: *Triticum urartu*, IT No.:177184, TUR; 3: *Triticum urartu*, IT No.:177185, ARM; 4: *Triticum aestivum* subsp. *aestivum*, IT No.:185166, UNK; 5: *Triticum urartu*, IT No.:202089, UNK; 6: *Aegilops biuncialis*, IT No.:204618, TUR; 7: *Triticum aestivum* subsp. *aestivum*, IT No.:267172, USA; 8: *Triticum turgidum* subsp. *durum*, IT No.:269514, MNG; 9: *Triticum turgidum* subsp. *durum*, IT No.:269515, MNG; 10: *Triticum aestivum* subsp. *aestivum*, IT No.:293620, CHN; 11: *Aegilops uniaristata*, IT No.:302223, GRC; 12: *Aegilops ventricosa*, IT No.:302224, FRA; 13: *Triticum aestivum* subsp. *aestivum*, IT No.:324662, TUR; 14: *Triticum turgidum* subsp. *durum*, IT No.:330427, CAN; 15: *Triticum urartu*, IT No.:330603, SYR; 16: *Triticum turgidum* subsp. *durum*, IT No.:340493, CAN; 17: *Triticum aestivum* subsp. *aestivum*, IT No.:341831, KOR; 18: *Aegilops cylindrica*, IT No.:352075, TJK).


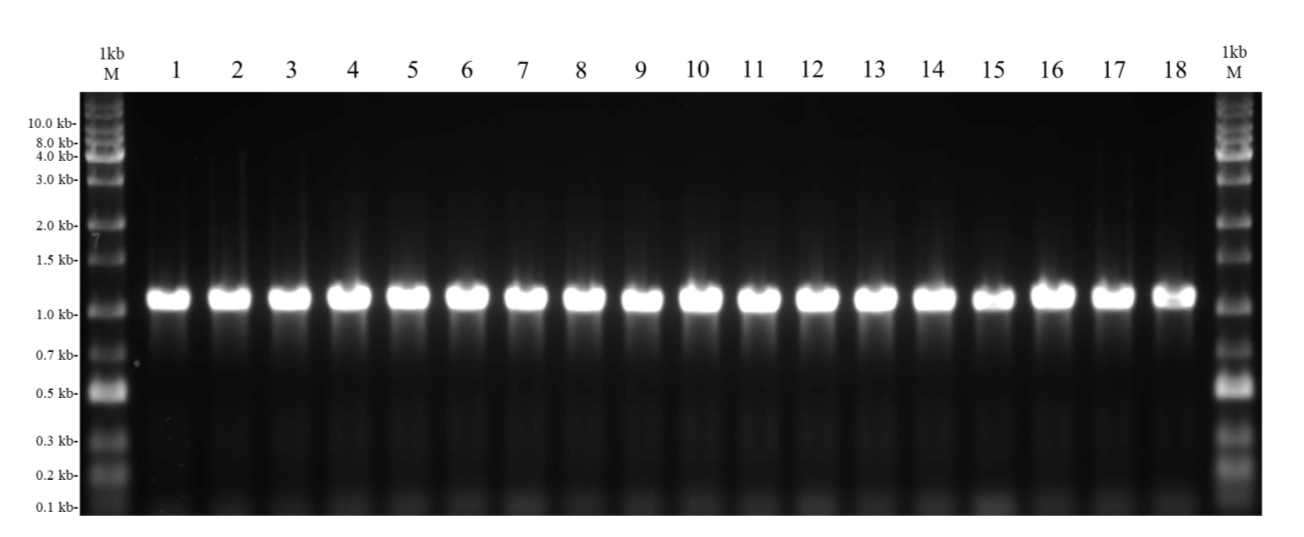


**Figure S7**. PCR amplification products of cultivar 1-18 for the specific barcoding marker *psbA* gene. M: DNA ladder. (1: *Triticum aestivum* subsp. *aestivum*, IT No.:172226, UNK; 2: *Triticum urartu*, IT No.:177184, TUR; 3: *Triticum urartu*, IT No.:177185, ARM; 4: *Triticum aestivum* subsp. *aestivum*, IT No.:185166, UNK; 5: *Triticum urartu*, IT No.:202089, UNK; 6: *Aegilops biuncialis*, IT No.:204618, TUR; 7: *Triticum aestivum* subsp. *aestivum*, IT No.:267172, USA; 8: *Triticum turgidum* subsp. *durum*, IT No.:269514, MNG; 9: *Triticum turgidum* subsp. *durum*, IT No.:269515, MNG; 10: *Triticum aestivum* subsp. *aestivum*, IT No.:293620, CHN; 11: *Aegilops uniaristata*, IT No.:302223, GRC; 12: *Aegilops ventricosa*, IT No.:302224, FRA; 13: *Triticum aestivum* subsp. *aestivum*, IT No.:324662, TUR; 14: *Triticum turgidum* subsp. *durum*, IT No.:330427, CAN; 15: *Triticum urartu*, IT No.:330603, SYR; 16: *Triticum turgidum* subsp. *durum*, IT No.:340493, CAN; 17: *Triticum aestivum* subsp. *aestivum*, IT No.:341831, KOR; 18: *Aegilops cylindrica*, IT No.:352075, TJK).


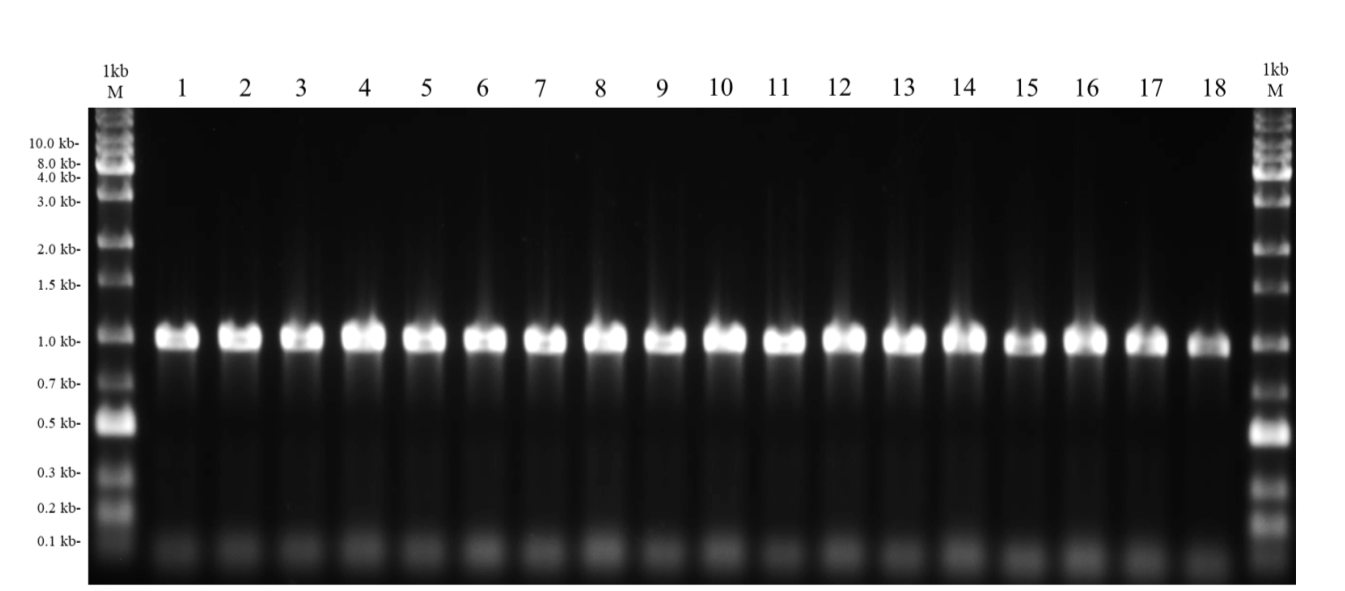


**Figure S8**. PCR amplification products of cultivar 1-18 for the specific barcoding marker *rpoA* gene. M: DNA ladder. (1: *Triticum aestivum* subsp. *aestivum*, IT No.:172226, UNK; 2: *Triticum urartu*, IT No.:177184, TUR; 3: *Triticum urartu*, IT No.:177185, ARM; 4: *Triticum aestivum* subsp. *aestivum*, IT No.:185166, UNK; 5: *Triticum urartu*, IT No.:202089, UNK; 6: *Aegilops biuncialis*, IT No.:204618, TUR; 7: *Triticum aestivum* subsp. *aestivum*, IT No.:267172, USA; 8: *Triticum turgidum* subsp. *durum*, IT No.:269514, MNG; 9: *Triticum turgidum* subsp. *durum*, IT No.:269515, MNG; 10: *Triticum aestivum* subsp. *aestivum*, IT No.:293620, CHN; 11: *Aegilops uniaristata*, IT No.:302223, GRC; 12: *Aegilops ventricosa*, IT No.:302224, FRA; 13: *Triticum aestivum* subsp. *aestivum*, IT No.:324662, TUR; 14: *Triticum turgidum* subsp. *durum*, IT No.:330427, CAN; 15: *Triticum urartu*, IT No.:330603, SYR; 16: *Triticum turgidum* subsp. *durum*, IT No.:340493, CAN; 17: *Triticum aestivum* subsp. *aestivum*, IT No.:341831, KOR; 18: *Aegilops cylindrica*, IT No.:352075, TJK).


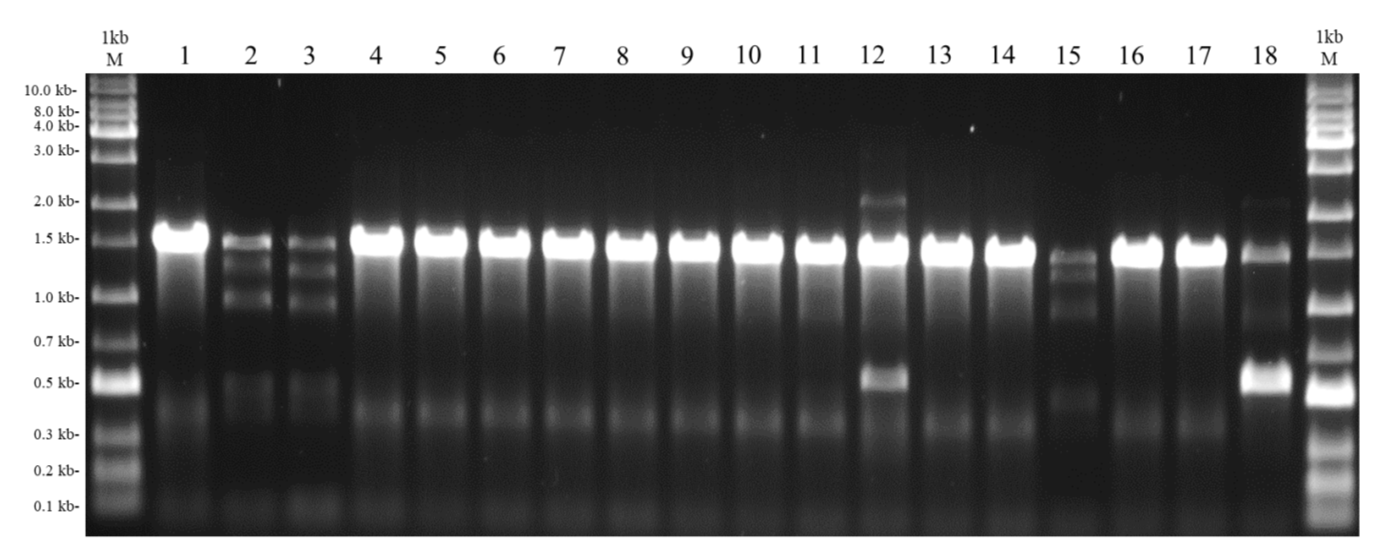


**Figure S9**. PCR amplification products of cultivar 1-18 for the specific barcoding marker *matK-rps16*. M: DNA ladder. (1: *Triticum aestivum* subsp. *aestivum*, IT No.:172226, UNK; 2: *Triticum urartu*, IT No.:177184, TUR; 3: *Triticum urartu*, IT No.:177185, ARM; 4: *Triticum aestivum* subsp. *aestivum*, IT No.:185166, UNK; 5: *Triticum urartu*, IT No.:202089, UNK; 6: *Aegilops biuncialis*, IT No.:204618, TUR; 7: *Triticum aestivum* subsp. *aestivum*, IT No.:267172, USA; 8: *Triticum turgidum* subsp. *durum*, IT No.:269514, MNG; 9: *Triticum turgidum* subsp. *durum*, IT No.:269515, MNG; 10: *Triticum aestivum* subsp. *aestivum*, IT No.:293620, CHN; 11: *Aegilops uniaristata*, IT No.:302223, GRC; 12: *Aegilops ventricosa*, IT No.:302224, FRA; 13: *Triticum aestivum* subsp. *aestivum*, IT No.:324662, TUR; 14: *Triticum turgidum* subsp. *durum*, IT No.:330427, CAN; 15: *Triticum urartu*, IT No.:330603, SYR; 16: *Triticum turgidum* subsp. *durum*, IT No.:340493, CAN; 17: *Triticum aestivum* subsp. *aestivum*, IT No.:341831, KOR; 18: *Aegilops cylindrica*, IT No.:352075, TJK).


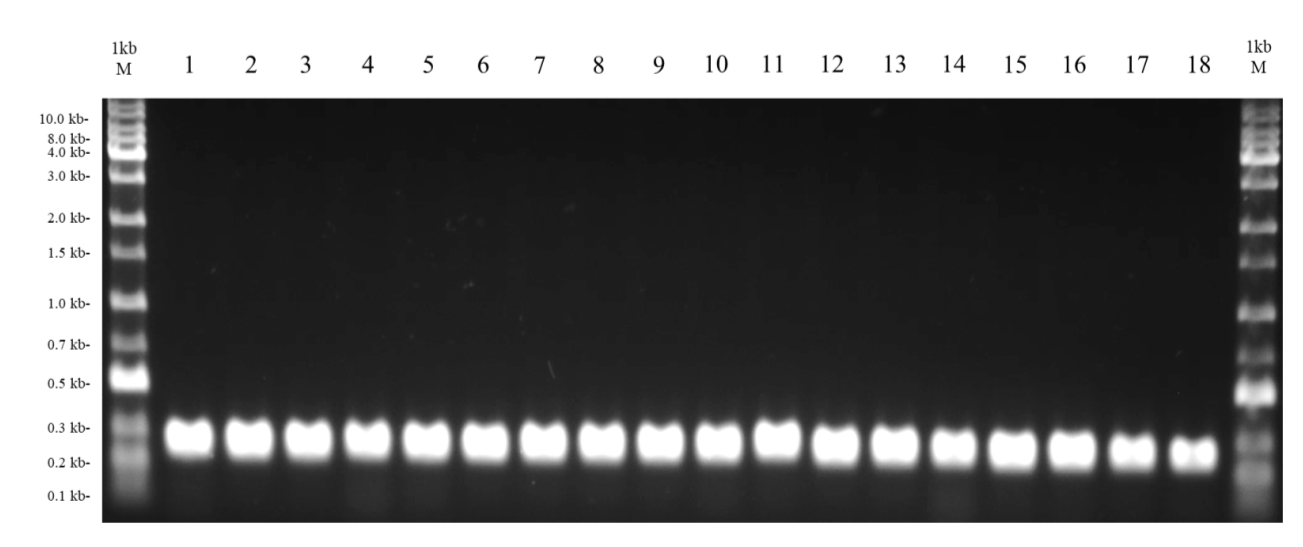


**Figure S10**. PCR amplification products of cultivar 1-18 for the specific barcoding marker *psbl-trnS-GCU*. M: DNA ladder. (1: *Triticum aestivum* subsp. *aestivum*, IT No.:172226, UNK; 2: *Triticum urartu*, IT No.:177184, TUR; 3: *Triticum urartu*, IT No.:177185, ARM; 4: *Triticum aestivum* subsp. *aestivum*, IT No.:185166, UNK; 5: *Triticum urartu*, IT No.:202089, UNK; 6: *Aegilops biuncialis*, IT No.:204618, TUR; 7: *Triticum aestivum* subsp. *aestivum*, IT No.:267172, USA; 8: *Triticum turgidum* subsp. *durum*, IT No.:269514, MNG; 9: *Triticum turgidum* subsp. *durum*, IT No.:269515, MNG; 10: *Triticum aestivum* subsp. *aestivum*, IT No.:293620, CHN; 11: *Aegilops uniaristata*, IT No.:302223, GRC; 12: *Aegilops ventricosa*, IT No.:302224, FRA; 13: *Triticum aestivum* subsp. *aestivum*, IT No.:324662, TUR; 14: *Triticum turgidum* subsp. *durum*, IT No.:330427, CAN; 15: *Triticum urartu*, IT No.:330603, SYR; 16: *Triticum turgidum* subsp. *durum*, IT No.:340493, CAN; 17: *Triticum aestivum* subsp. *aestivum*, IT No.:341831, KOR; 18: *Aegilops cylindrica*, IT No.:352075, TJK).


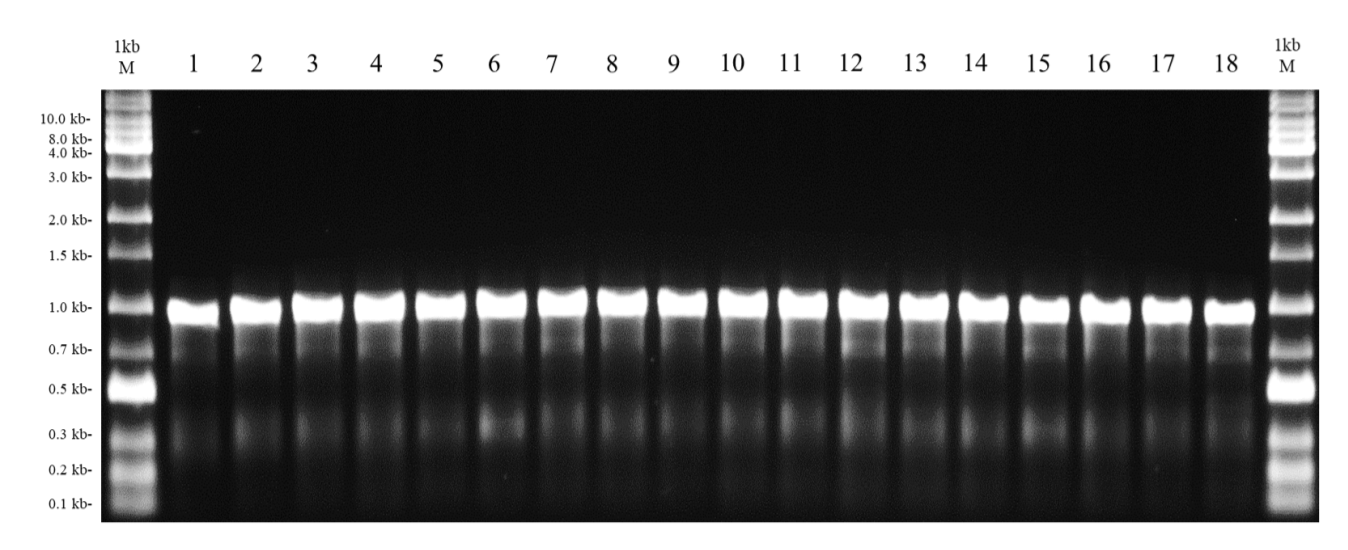


**Figure S11**. PCR amplification products of cultivar 1-18 for the specific barcoding marker *atpF-intron*. M: DNA ladder. (1: *Triticum aestivum* subsp. *aestivum*, IT No.:172226, UNK; 2: *Triticum urartu*, IT No.:177184, TUR; 3: *Triticum urartu*, IT No.:177185, ARM; 4: *Triticum aestivum* subsp. *aestivum*, IT No.:185166, UNK; 5: *Triticum urartu*, IT No.:202089, UNK; 6: *Aegilops biuncialis*, IT No.:204618, TUR; 7: *Triticum aestivum* subsp. *aestivum*, IT No.:267172, USA; 8: *Triticum turgidum* subsp. *durum*, IT No.:269514, MNG; 9: *Triticum turgidum* subsp. *durum*, IT No.:269515, MNG; 10: *Triticum aestivum* subsp. *aestivum*, IT No.:293620, CHN; 11: *Aegilops uniaristata*, IT No.:302223, GRC; 12: *Aegilops ventricosa*, IT No.:302224, FRA; 13: *Triticum aestivum* subsp. *aestivum*, IT No.:324662, TUR; 14: *Triticum turgidum* subsp. *durum*, IT No.:330427, CAN; 15: *Triticum urartu*, IT No.:330603, SYR; 16: *Triticum turgidum* subsp. *durum*, IT No.:340493, CAN; 17: *Triticum aestivum* subsp. *aestivum*, IT No.:341831, KOR; 18: *Aegilops cylindrica*, IT No.:352075, TJK).


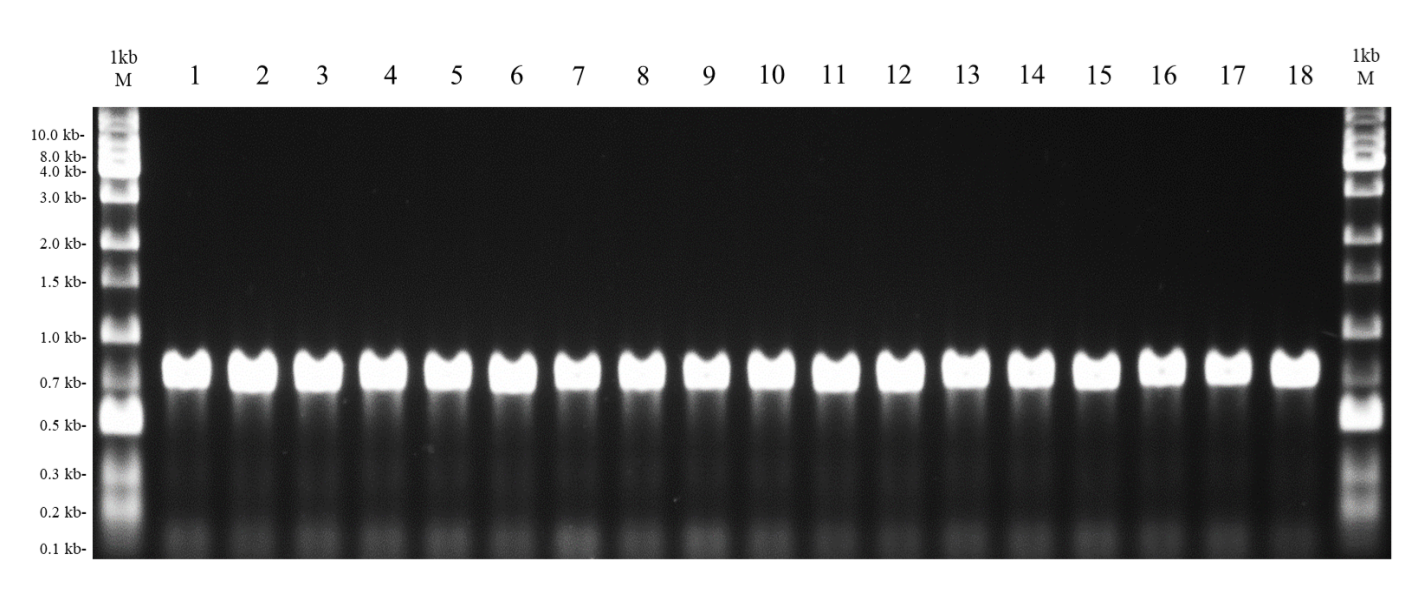


**Figure S12**. PCR amplification products of cultivar 1-18 for the specific barcoding marker *psaA-ycf3*. M: DNA ladder. (1: *Triticum aestivum* subsp. *aestivum*, IT No.:172226, UNK; 2: *Triticum urartu*, IT No.:177184, TUR; 3: *Triticum urartu*, IT No.:177185, ARM; 4: *Triticum aestivum* subsp. *aestivum*, IT No.:185166, UNK; 5: *Triticum urartu*, IT No.:202089, UNK; 6: *Aegilops biuncialis*, IT No.:204618, TUR; 7: *Triticum aestivum* subsp. *aestivum*, IT No.:267172, USA; 8: *Triticum turgidum* subsp. *durum*, IT No.:269514, MNG; 9: *Triticum turgidum* subsp. *durum*, IT No.:269515, MNG; 10: *Triticum aestivum* subsp. *aestivum*, IT No.:293620, CHN; 11: *Aegilops uniaristata*, IT No.:302223, GRC; 12: *Aegilops ventricosa*, IT No.:302224, FRA; 13: *Triticum aestivum* subsp. *aestivum*, IT No.:324662, TUR; 14: *Triticum turgidum* subsp. *durum*, IT No.:330427, CAN; 15: *Triticum urartu*, IT No.:330603, SYR; 16: *Triticum turgidum* subsp. *durum*, IT No.:340493, CAN; 17: *Triticum aestivum* subsp. *aestivum*, IT No.:341831, KOR; 18: *Aegilops cylindrica*, IT No.:352075, TJK).


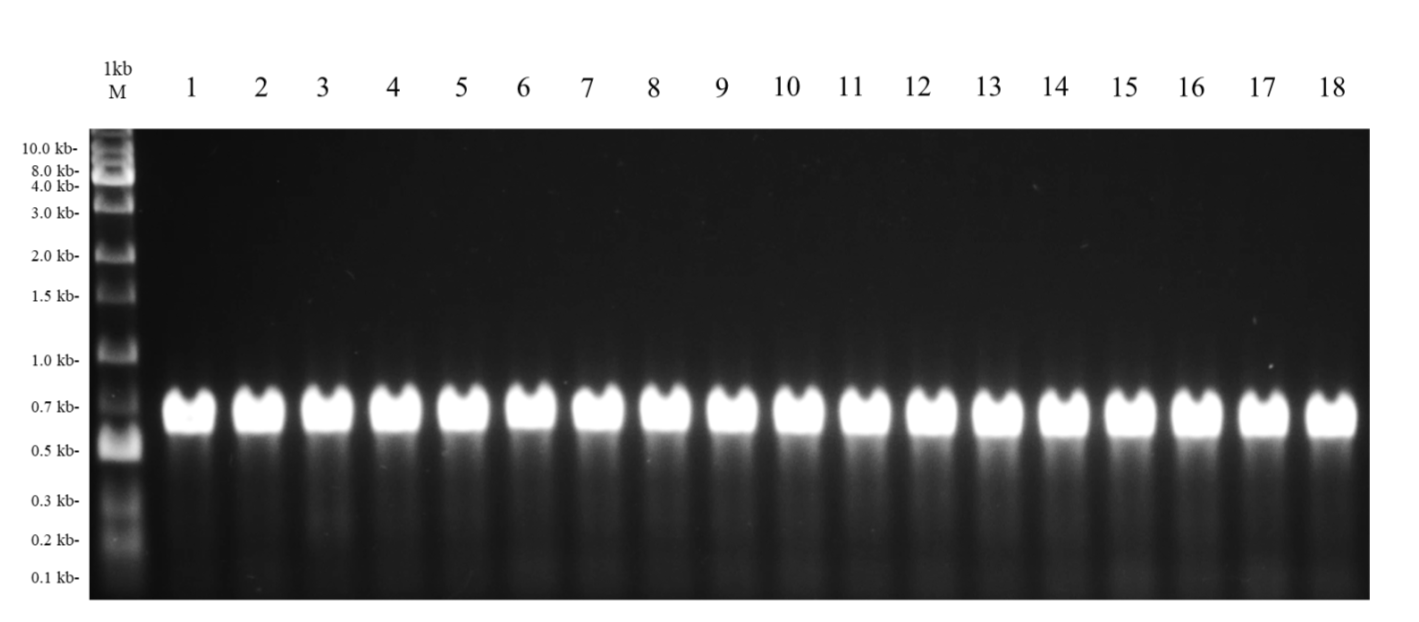


**Figure S13**. PCR amplification products of cultivar 1-18 for the specific barcoding marker *trnT-UGU-trnL-UAA*. M: DNA ladder. (1: *Triticum aestivum* subsp. *aestivum*, IT No.:172226, UNK; 2: *Triticum urartu*, IT No.:177184, TUR; 3: *Triticum urartu*, IT No.:177185, ARM; 4: *Triticum aestivum* subsp. *aestivum*, IT No.:185166, UNK; 5: *Triticum urartu*, IT No.:202089, UNK; 6: *Aegilops biuncialis*, IT No.:204618, TUR; 7: *Triticum aestivum* subsp. *aestivum*, IT No.:267172, USA; 8: *Triticum turgidum* subsp. *durum*, IT No.:269514, MNG; 9: *Triticum turgidum* subsp. *durum*, IT No.:269515, MNG; 10: *Triticum aestivum* subsp. *aestivum*, IT No.:293620, CHN; 11: *Aegilops uniaristata*, IT No.:302223, GRC; 12: *Aegilops ventricosa*, IT No.:302224, FRA; 13: *Triticum aestivum* subsp. *aestivum*, IT No.:324662, TUR; 14: *Triticum turgidum* subsp. *durum*, IT No.:330427, CAN; 15: *Triticum urartu*, IT No.:330603, SYR; 16: *Triticum turgidum* subsp. *durum*, IT No.:340493, CAN; 17: *Triticum aestivum* subsp. *aestivum*, IT No.:341831, KOR; 18: *Aegilops cylindrica*, IT No.:352075, TJK).


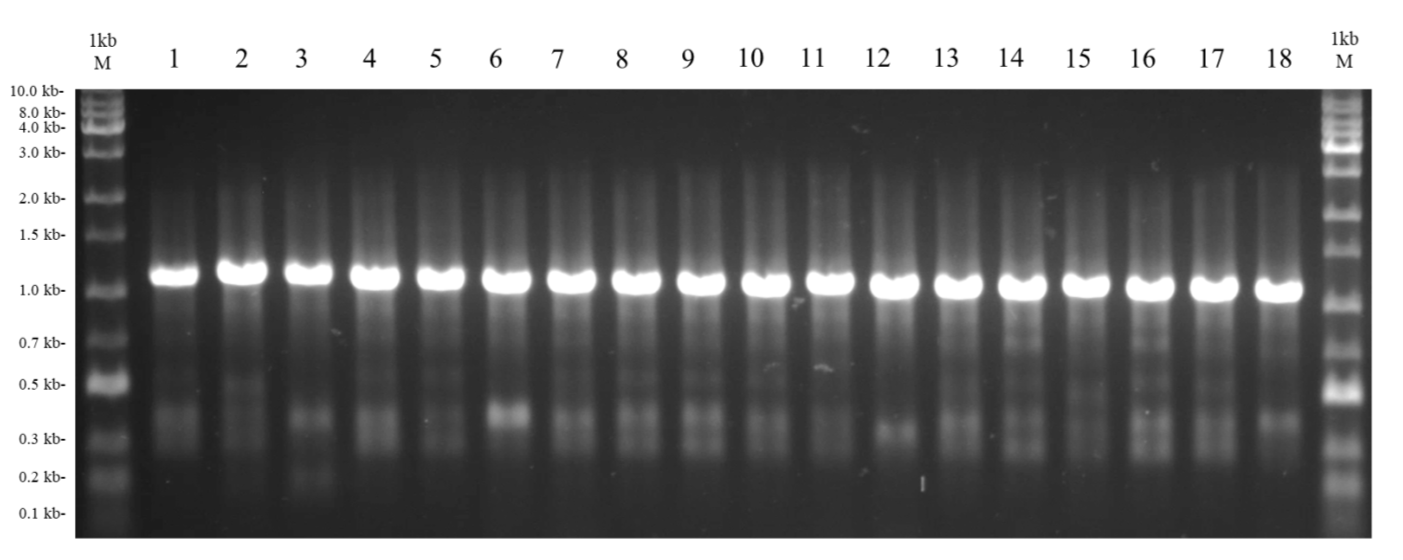


**Figure S14**. PCR amplification products of cultivar 1-18 for the specific barcoding marker *trnL-UAA-trnF-GAA*. M: DNA ladder. (1: *Triticum aestivum* subsp. *aestivum*, IT No.:172226, UNK; 2: *Triticum urartu*, IT No.:177184, TUR; 3: *Triticum urartu*, IT No.:177185, ARM; 4: *Triticum aestivum* subsp. *aestivum*, IT No.:185166, UNK; 5: *Triticum urartu*, IT No.:202089, UNK; 6: *Aegilops biuncialis*, IT No.:204618, TUR; 7: *Triticum aestivum* subsp. *aestivum*, IT No.:267172, USA; 8: *Triticum turgidum* subsp. *durum*, IT No.:269514, MNG; 9: *Triticum turgidum* subsp. *durum*, IT No.:269515, MNG; 10: *Triticum aestivum* subsp. *aestivum*, IT No.:293620, CHN; 11: *Aegilops uniaristata*, IT No.:302223, GRC; 12: *Aegilops ventricosa*, IT No.:302224, FRA; 13: *Triticum aestivum* subsp. *aestivum*, IT No.:324662, TUR; 14: *Triticum turgidum* subsp. *durum*, IT No.:330427, CAN; 15: *Triticum urartu*, IT No.:330603, SYR; 16: *Triticum turgidum* subsp. *durum*, IT No.:340493, CAN; 17: *Triticum aestivum* subsp. *aestivum*, IT No.:341831, KOR; 18: *Aegilops cylindrica*, IT No.:352075, TJK).


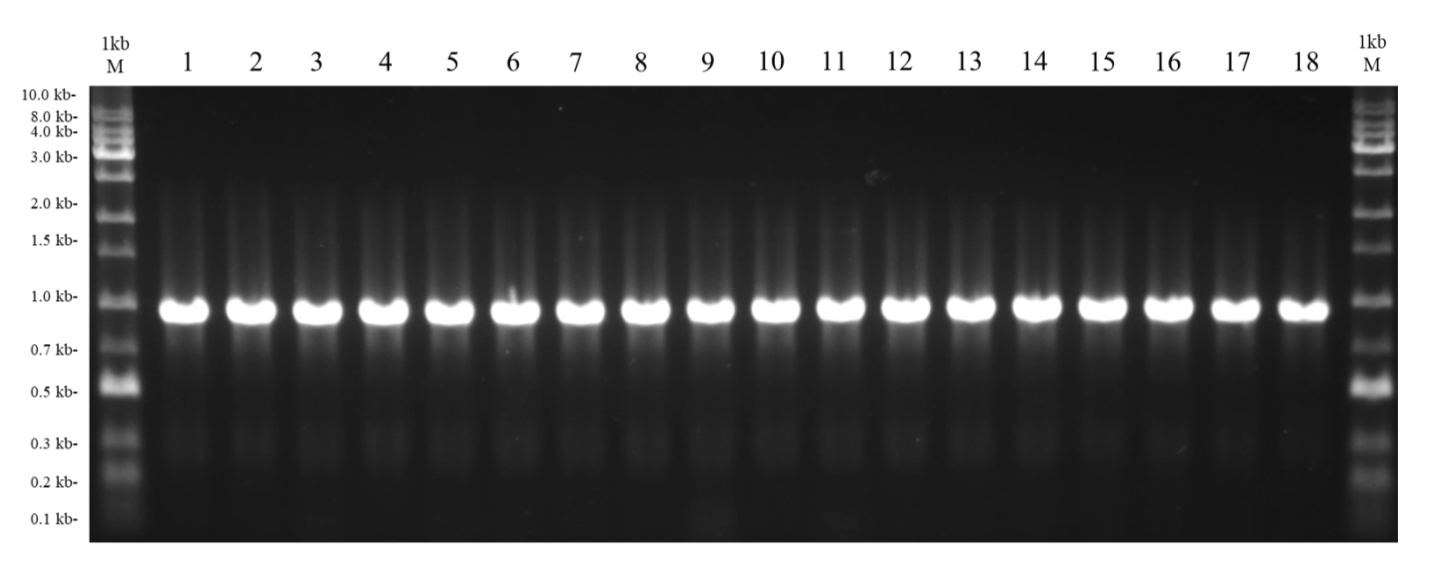


**Figure S15**. PCR amplification products of cultivar 1-18 for the specific barcoding marker *petA-psbJ*. M: DNA ladder. (1: *Triticum aestivum* subsp. *aestivum*, IT No.:172226, UNK; 2: *Triticum urartu*, IT No.:177184, TUR; 3: *Triticum urartu*, IT No.:177185, ARM; 4: *Triticum aestivum* subsp. *aestivum*, IT No.:185166, UNK; 5: *Triticum urartu*, IT No.:202089, UNK; 6: *Aegilops biuncialis*, IT No.:204618, TUR; 7: *Triticum aestivum* subsp. *aestivum*, IT No.:267172, USA; 8: *Triticum turgidum* subsp. *durum*, IT No.:269514, MNG; 9: *Triticum turgidum* subsp. *durum*, IT No.:269515, MNG; 10: *Triticum aestivum* subsp. *aestivum*, IT No.:293620, CHN; 11: *Aegilops uniaristata*, IT No.:302223, GRC; 12: *Aegilops ventricosa*, IT No.:302224, FRA; 13: *Triticum aestivum* subsp. *aestivum*, IT No.:324662, TUR; 14: *Triticum turgidum* subsp. *durum*, IT No.:330427, CAN; 15: *Triticum urartu*, IT No.:330603, SYR; 16: *Triticum turgidum* subsp. *durum*, IT No.:340493, CAN; 17: *Triticum aestivum* subsp. *aestivum*, IT No.:341831, KOR; 18: *Aegilops cylindrica*, IT No.:352075, TJK).

**
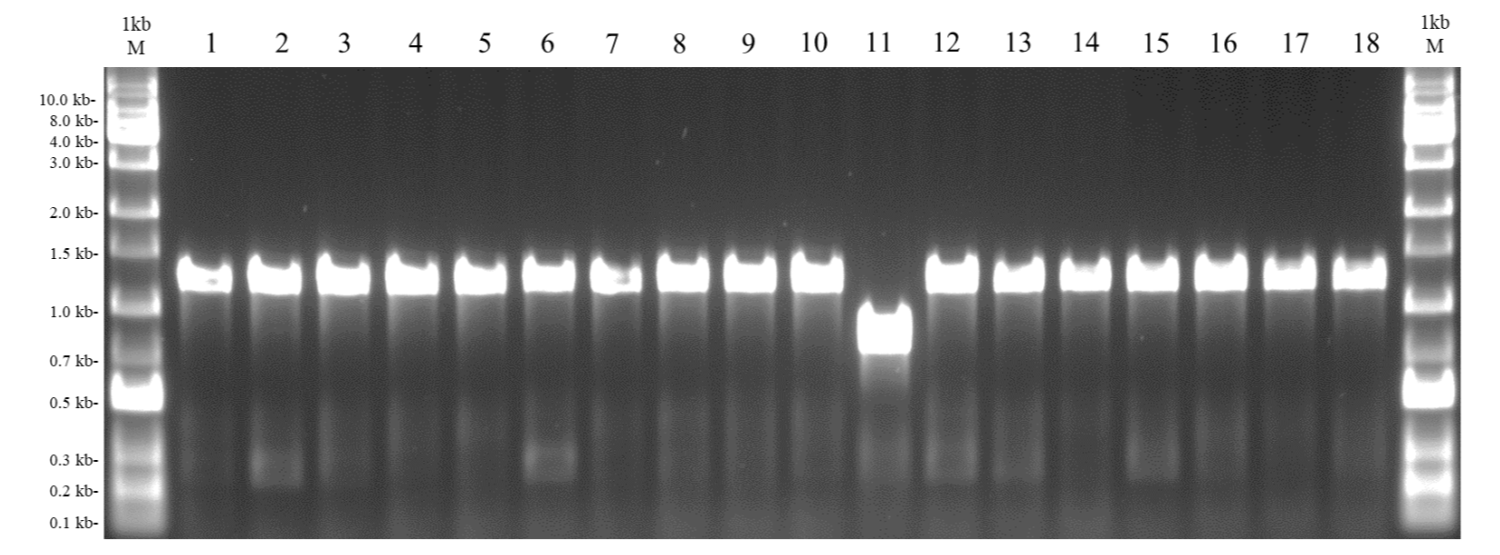
**

**Figure S16**. PCR amplification products of cultivar 1-18 for the specific barcoding marker *psbE-petL*. M: DNA ladder. (1: *Triticum aestivum* subsp. *aestivum*, IT No.:172226, UNK; 2: *Triticum urartu*, IT No.:177184, TUR; 3: *Triticum urartu*, IT No.:177185, ARM; 4: *Triticum aestivum* subsp. *aestivum*, IT No.:185166, UNK; 5: *Triticum urartu*, IT No.:202089, UNK; 6: *Aegilops biuncialis*, IT No.:204618, TUR; 7: *Triticum aestivum* subsp. *aestivum*, IT No.:267172, USA; 8: *Triticum turgidum* subsp. *durum*, IT No.:269514, MNG; 9: *Triticum turgidum* subsp. *durum*, IT No.:269515, MNG; 10: *Triticum aestivum* subsp. *aestivum*, IT No.:293620, CHN; 11: *Aegilops uniaristata*, IT No.:302223, GRC; 12: *Aegilops ventricosa*, IT No.:302224, FRA; 13: *Triticum aestivum* subsp. *aestivum*, IT No.:324662, TUR; 14: *Triticum turgidum* subsp. *durum*, IT No.:330427, CAN; 15: *Triticum urartu*, IT No.:330603, SYR; 16: *Triticum turgidum* subsp. *durum*, IT No.:340493, CAN; 17: *Triticum aestivum* subsp. *aestivum*, IT No.:341831, KOR; 18: *Aegilops cylindrica*, IT No.:352075, TJK).


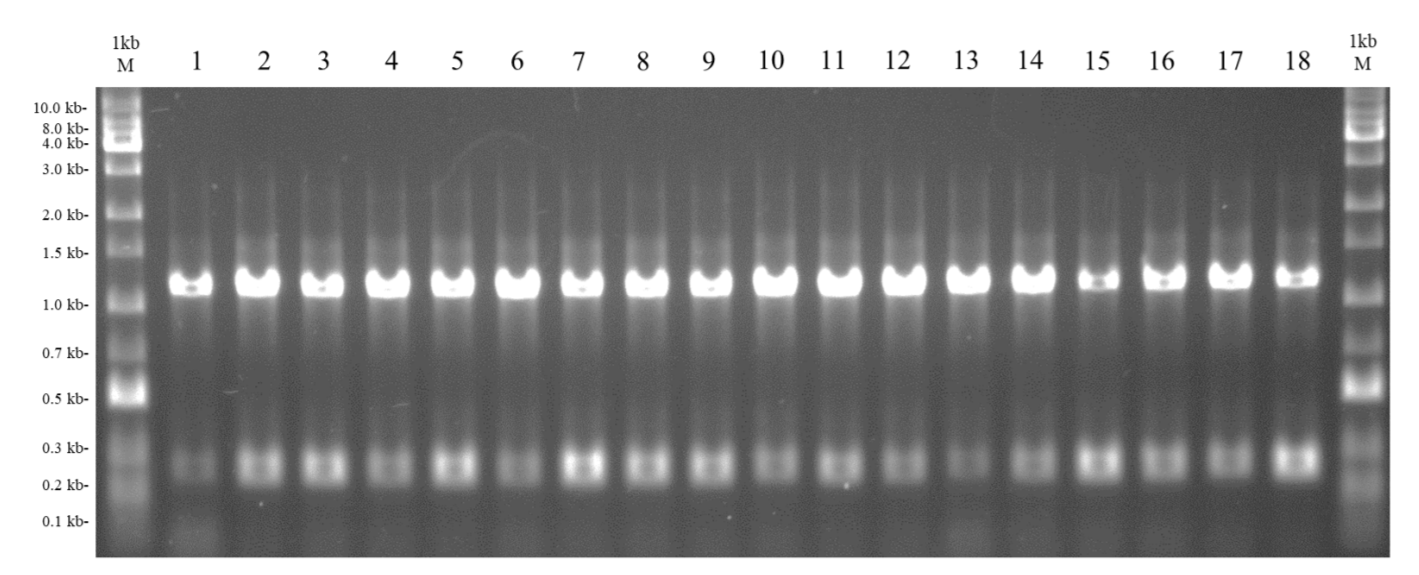


**Figure S17**. PCR amplification products of cultivar 1-18 for the specific barcoding marker *rpl16-rps3*. M: DNA ladder. (1: *Triticum aestivum* subsp. *aestivum*, IT No.:172226, UNK; 2: *Triticum urartu*, IT No.:177184, TUR; 3: *Triticum urartu*, IT No.:177185, ARM; 4: *Triticum aestivum* subsp. *aestivum*, IT No.:185166, UNK; 5: *Triticum urartu*, IT No.:202089, UNK; 6: *Aegilops biuncialis*, IT No.:204618, TUR; 7: *Triticum aestivum* subsp. *aestivum*, IT No.:267172, USA; 8: *Triticum turgidum* subsp. *durum*, IT No.:269514, MNG; 9: *Triticum turgidum* subsp. *durum*, IT No.:269515, MNG; 10: *Triticum aestivum* subsp. *aestivum*, IT No.:293620, CHN; 11: *Aegilops uniaristata*, IT No.:302223, GRC; 12: *Aegilops ventricosa*, IT No.:302224, FRA; 13: *Triticum aestivum* subsp. *aestivum*, IT No.:324662, TUR; 14: *Triticum turgidum* subsp. *durum*, IT No.:330427, CAN; 15: *Triticum urartu*, IT No.:330603, SYR; 16: *Triticum turgidum* subsp. *durum*, IT No.:340493, CAN; 17: *Triticum aestivum* subsp. *aestivum*, IT No.:341831, KOR; 18: *Aegilops cylindrica*, IT No.:352075, TJK).


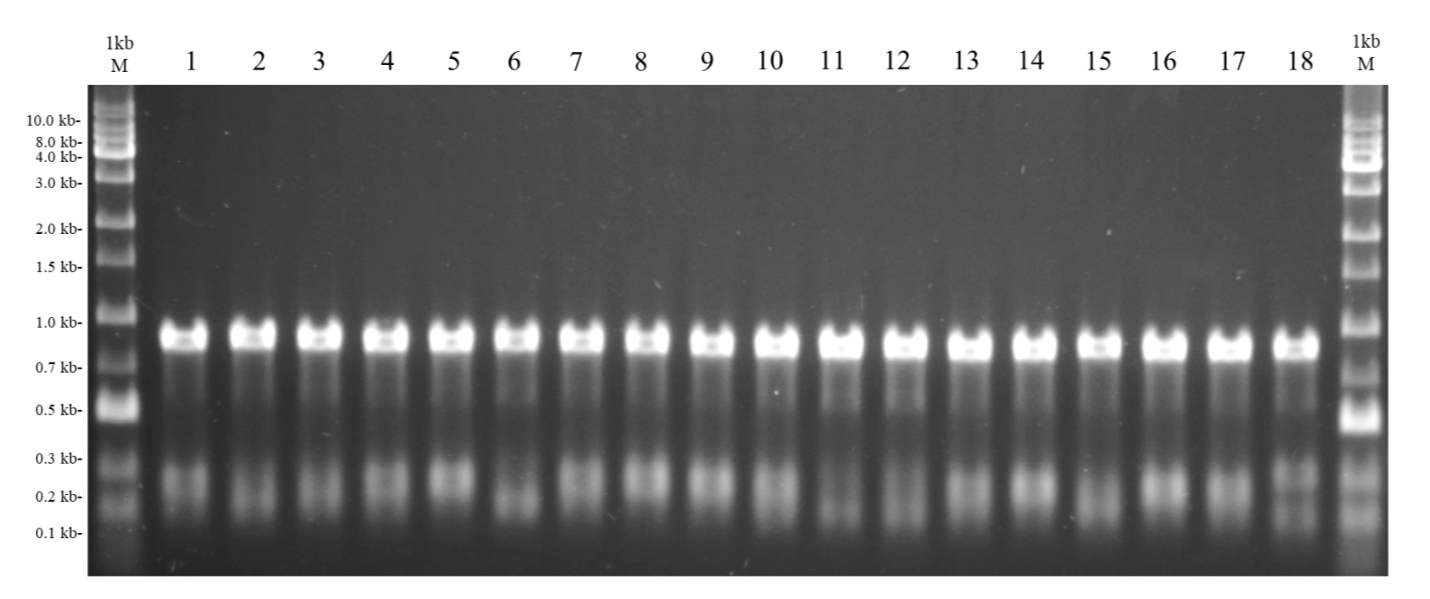


**Figure S18**. PCR amplification products of cultivar 1-18 for the specific barcoding marker *rpl32-trnL-UAG*. M: DNA ladder. (1: *Triticum aestivum* subsp. *aestivum*, IT No.:172226, UNK; 2: *Triticum urartu*, IT No.:177184, TUR; 3: *Triticum urartu*, IT No.:177185, ARM; 4: *Triticum aestivum* subsp. *aestivum*, IT No.:185166, UNK; 5: *Triticum urartu*, IT No.:202089, UNK; 6: *Aegilops biuncialis*, IT No.:204618, TUR; 7: *Triticum aestivum* subsp. *aestivum*, IT No.:267172, USA; 8: *Triticum turgidum* subsp. *durum*, IT No.:269514, MNG; 9: *Triticum turgidum* subsp. *durum*, IT No.:269515, MNG; 10: *Triticum aestivum* subsp. *aestivum*, IT No.:293620, CHN; 11: *Aegilops uniaristata*, IT No.:302223, GRC; 12: *Aegilops ventricosa*, IT No.:302224, FRA; 13: *Triticum aestivum* subsp. *aestivum*, IT No.:324662, TUR; 14: *Triticum turgidum* subsp. *durum*, IT No.:330427, CAN; 15: *Triticum urartu*, IT No.:330603, SYR; 16: *Triticum turgidum* subsp. *durum*, IT No.:340493, CAN; 17: *Triticum aestivum* subsp. *aestivum*, IT No.:341831, KOR; 18: *Aegilops cylindrica*, IT No.:352075, TJK).


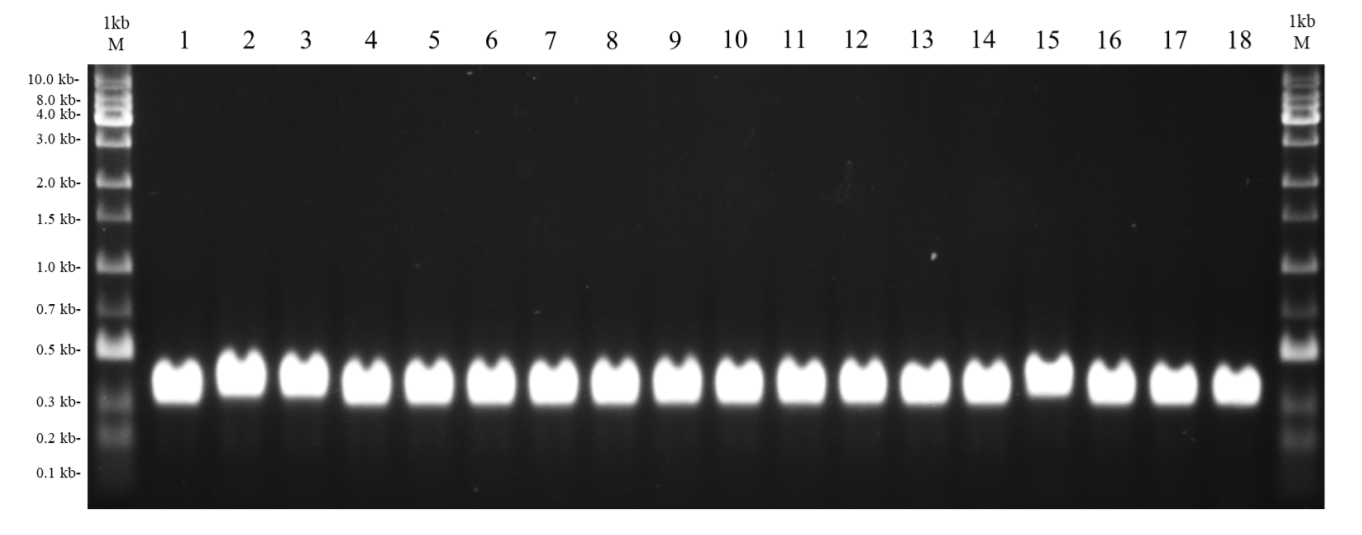


**Figure S19**. PCR amplification products of cultivar 1-18 for the specific barcoding marker *ccsA-ndhD*. M: DNA ladder. (1: *Triticum aestivum* subsp. *aestivum*, IT No.:172226, UNK; 2: *Triticum urartu*, IT No.:177184, TUR; 3: *Triticum urartu*, IT No.:177185, ARM; 4: *Triticum aestivum* subsp. *aestivum*, IT No.:185166, UNK; 5: *Triticum urartu*, IT No.:202089, UNK; 6: *Aegilops biuncialis*, IT No.:204618, TUR; 7: *Triticum aestivum* subsp. *aestivum*, IT No.:267172, USA; 8: *Triticum turgidum* subsp. *durum*, IT No.:269514, MNG; 9: *Triticum turgidum* subsp. *durum*, IT No.:269515, MNG; 10: *Triticum aestivum* subsp. *aestivum*, IT No.:293620, CHN; 11: *Aegilops uniaristata*, IT No.:302223, GRC; 12: *Aegilops ventricosa*, IT No.:302224, FRA; 13: *Triticum aestivum* subsp. *aestivum*, IT No.:324662, TUR; 14: *Triticum turgidum* subsp. *durum*, IT No.:330427, CAN; 15: *Triticum urartu*, IT No.:330603, SYR; 16: *Triticum turgidum* subsp. *durum*, IT No.:340493, CAN; 17: *Triticum aestivum* subsp. *aestivum*, IT No.:341831, KOR; 18: *Aegilops cylindrica*, IT No.:352075, TJK).
